# Supplementary material for: Cryo-EM led analysis of open and closed conformations of Chagas vaccine candidate TcPOP
Source: Nat Commun. 2025 Aug 5;16:7164. doi: 10.1038/s41467-025-62068-3 (PMC12325989; doi:10.1038/s41467-025-62068-3)
Supplement: Supplementary file 1 — Supplementary Information [file 41467_2025_62068_MOESM1_ESM.pdf]

Supplementary Information for

Cryo-EM led analysis of open and closed conformations of Chagas vaccine candidate TcPOP

*Sagar Batra, Francisco Olmo, Timothy J Ragan, Merve Kaplan, Valeria Calvaresi, Asger Meldgaard Frank, Claudia Lancey, Mahya Assadipapari, Cuifeng Ying, Weston B. Struwe, Emma Hesketh, John M. Kelly, Lea Barfod, and Ivan Campeotto**

The PDF file includes:

- Supplementary Figures S1 to S23
- Supplementary Tables S1 and S2

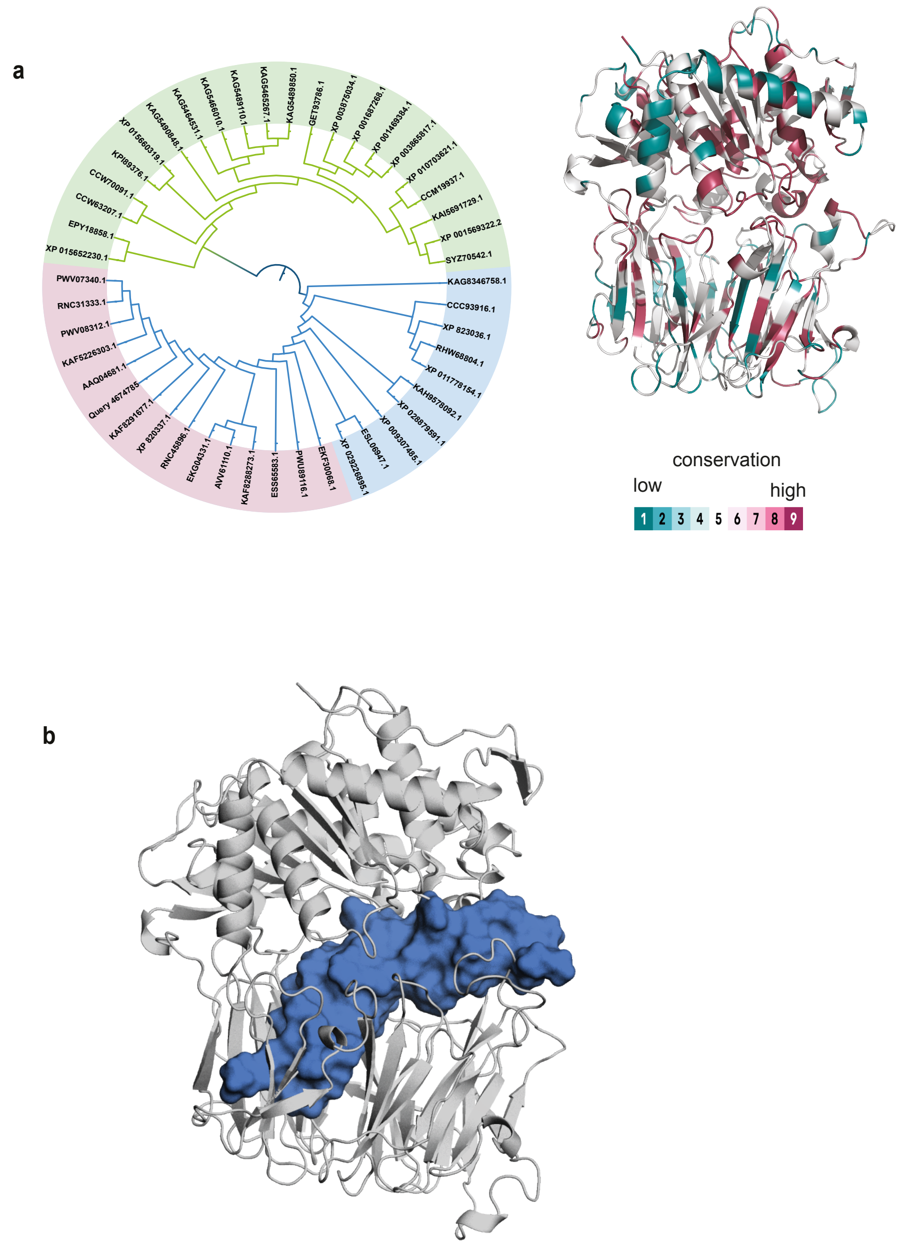

Supplementary Fig. S1 | Phylogenetic analysis of POPs | a, Phylogenetic analysis of POP sequence conservation across kinetoplastid parasite species using iTOL, coloured by species (*Trypanosoma cruzi* in red, another *Trypanosoma* sp. in blue and *Leishmania* sp. in green). Sequence conservation was mapped with CONSURF on the AlphaFold3 model of TcPOP, highlighting the conservation across the family in the two domains (ranging from cyan to purple, i.e., low to high conservation, respectively). **b**,

AlphaFold3 model of TcPOP complexed with collagen monomer (blue) extracted from PDB 1BKV. Cartoon representations were made in PyMOL.

a

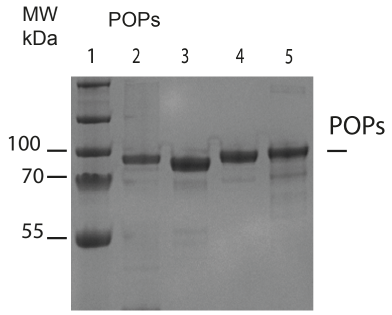

b

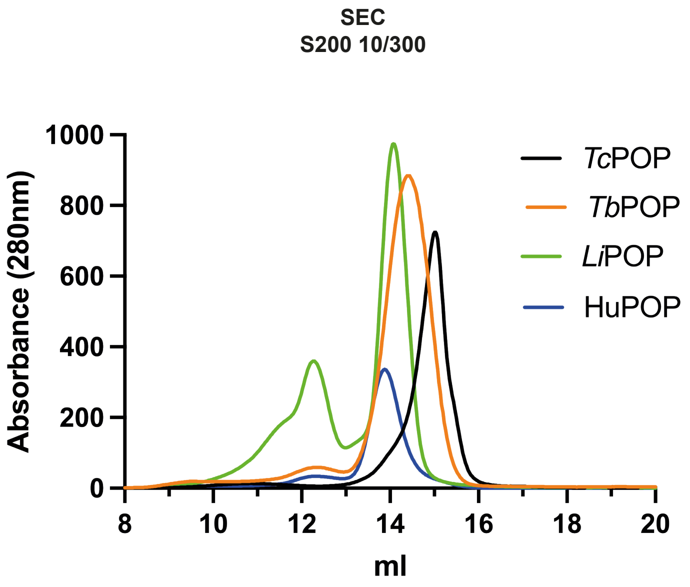

Supplementary Fig. S2 | Expression and purification of POPs. a, 12-4% SDS-PAGE of SEC purified POPs, 1=MW, 2=TcPOP, 3=TbPOP, 4=LiPOP, 5=HuPOP. Same gel as in Fig. 1 but showing extra lanes (3, 4 and 5). **b**, SEC analysis of individual POPs. Proteins were injected in a S200 10/300 column (Cytiva) and eluted as a single monodisperse peak at ~ 13mL of elution volume (Ve), with the exception of *Leishmania infantum* POP (LiPOP), where a dimer was also observed (green, ~ 12mL Ve).

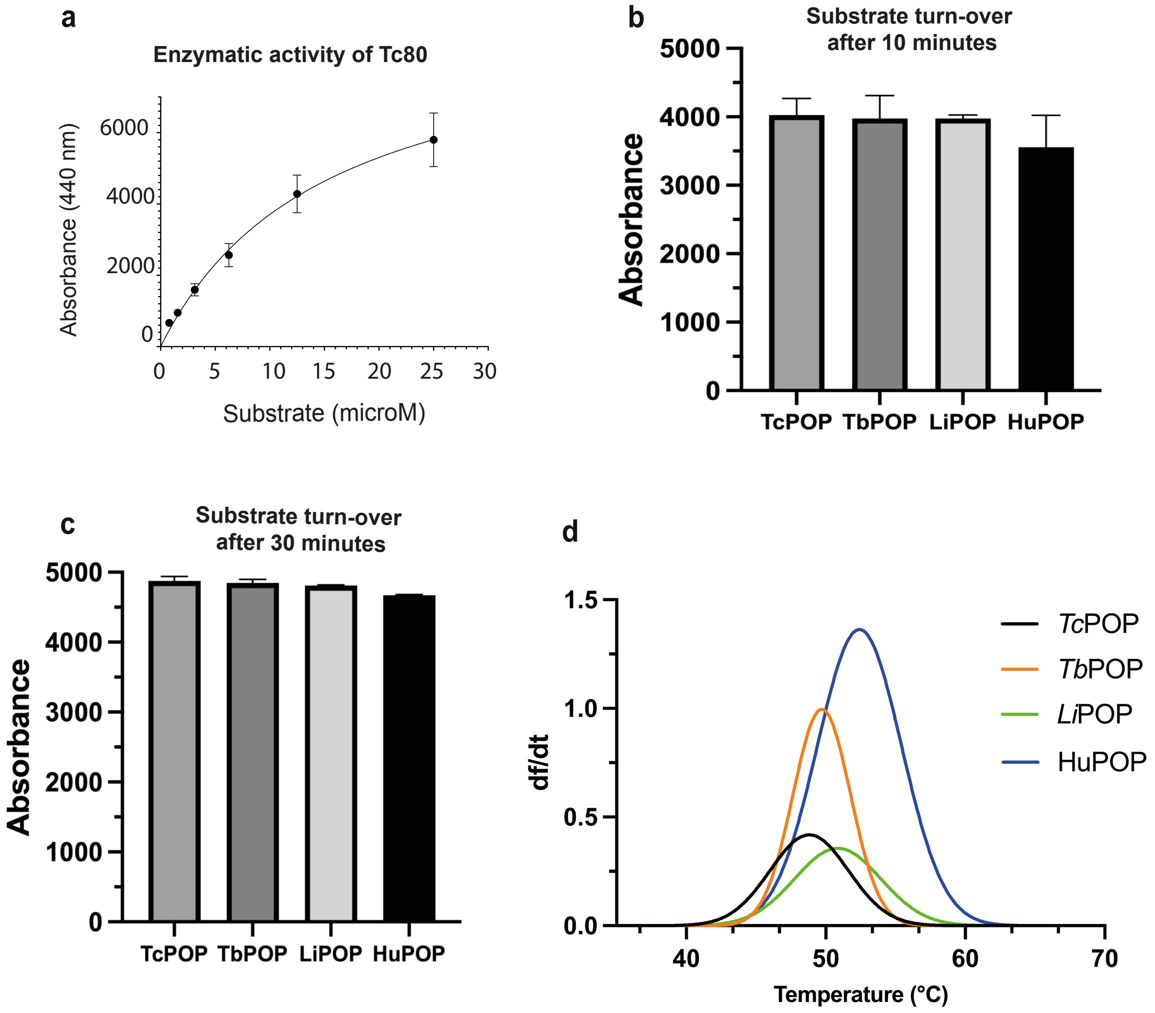

Supplementary Fig. S3 | Enzymatic activity of POPs. a, Digestion of substrate-mimicking peptide Gly-Pro-Leu-Gly-Pro-AMC by TcPOP, added at different concentrations at 25°C in 1 x PBS. The substrate concentration was therefore fixed to 25µM for end-point reactions by halting the reaction after **b,** 10 min or **c**, 30 min by addition of 100% EtOH. **d,** Heat stability of POPs measured by DSF. The assay was performed with a final protein concentration of 1 µM in a total volume of 20 µL. The temperature of the protein samples gradually increased from 10°C to 95°C at a rate of 5°C per minute, using the Rotor-Gene

Q (Qiagen). Experiments were performed in triplicate. The Data with error bars represents as standard error of the mean (SEM)

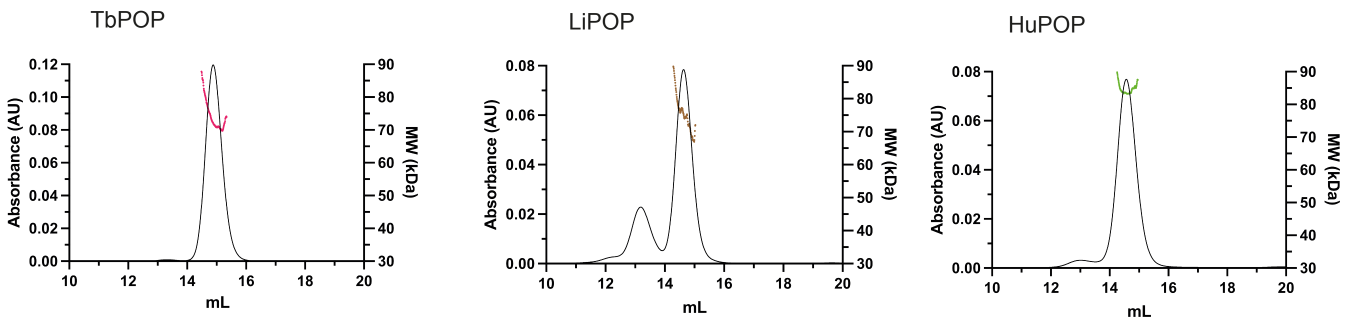

Supplementary Fig. S4 | SEC-MALS analysis. Protein samples were analysed usin g a S200 10/300 which confirmed the theoretical molecular mass values for TbPOP, LiPOP and HuPOP of 73.7 +/- 1.6%, 75.7 +/- 3.5% and 85.3 +/- 1.7% kDa respectively, corresponding to ~15 mL elution volume. The presence of a LiPOP dimer was confirmed by an upfront peak of 150.7 kDa +/- 6.1% at ~13ml elution volume.

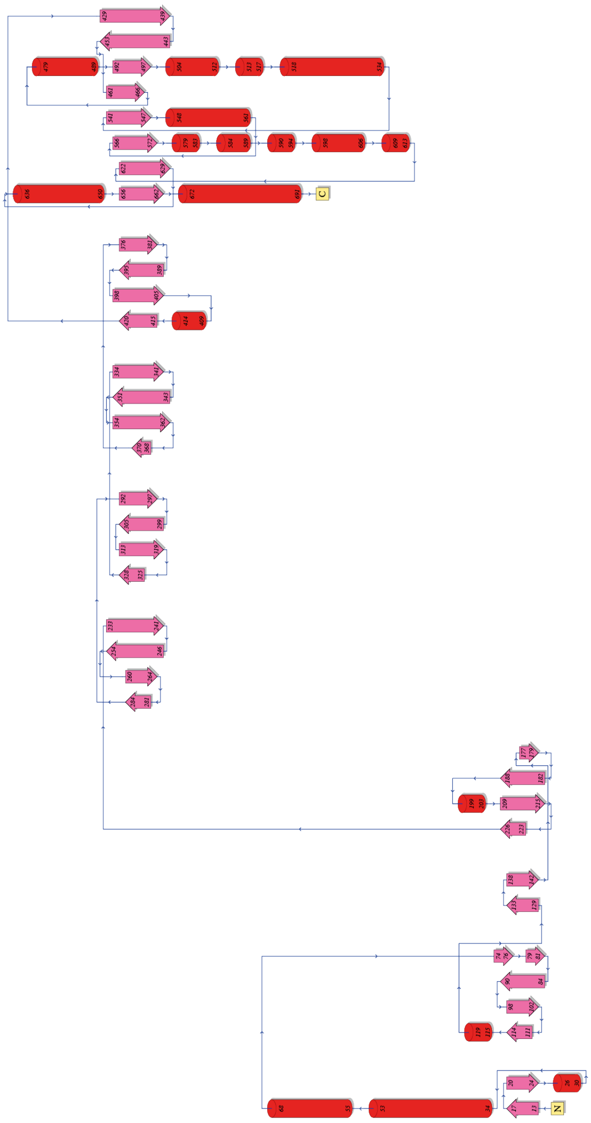

Supplementary Fig. S5 | Protein topology of TcPOP. The topology of TcPOP was generated using PDBsum. Alpha-helices are depicted as cylinders, whilst beta-strands are depicted as arrows. Amino-acid numbering is indicated according to sequence position.

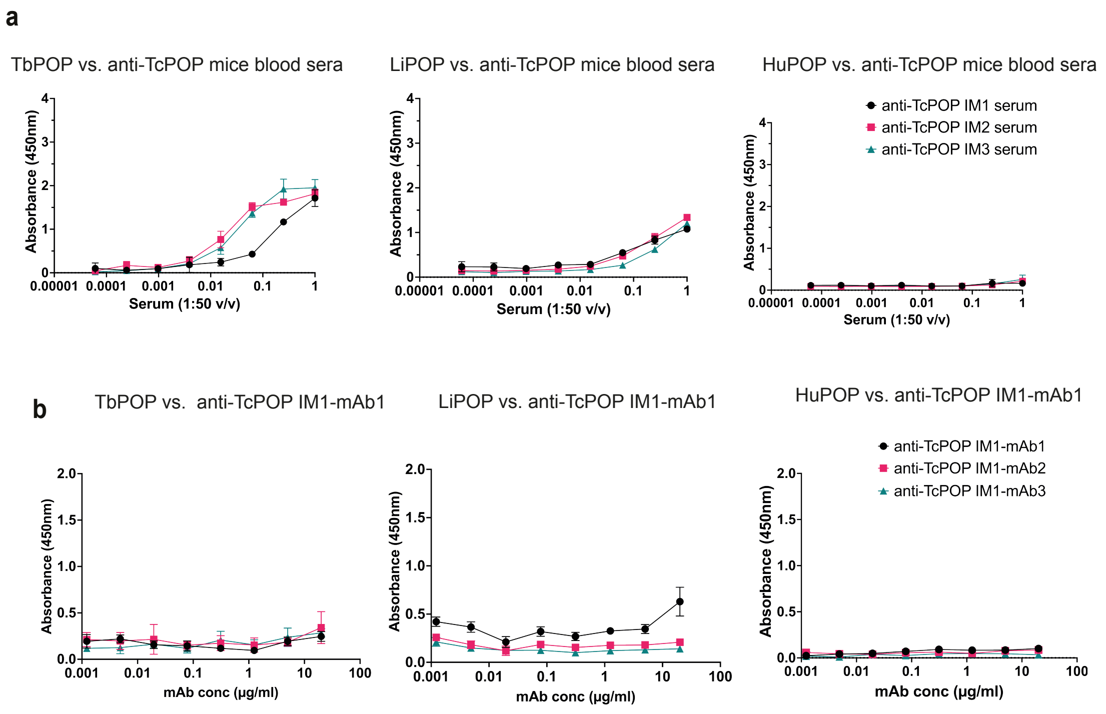

Supplementary Fig. S6 | ELISA testing of cross-reactivity of anti-TcPOP polyclonal and monoclonal responses against recombinant POPs. a, TcPOP was used to immunise mice and polyclonal serum (diluted 1:50) v/v was tested against recombinant TbPOP, LiPOP and HuPOP to assess cross-species reactivity. Buffer alone or BSA were used as controls. **b**, Three monoclonal IgG1 antibodies (IM1-mAbs 1-3) were isolated and purified from hybridomas and tested by ELISA in plates coated with: *T.i* POP (TbPOP) (B), *L. infantum* POP (LiPOP) (C) or *Homo sapiens* POP (HuPOP). Experiments were performed in triplicates. The Data with error bars represents as standard error of the mean (SEM).

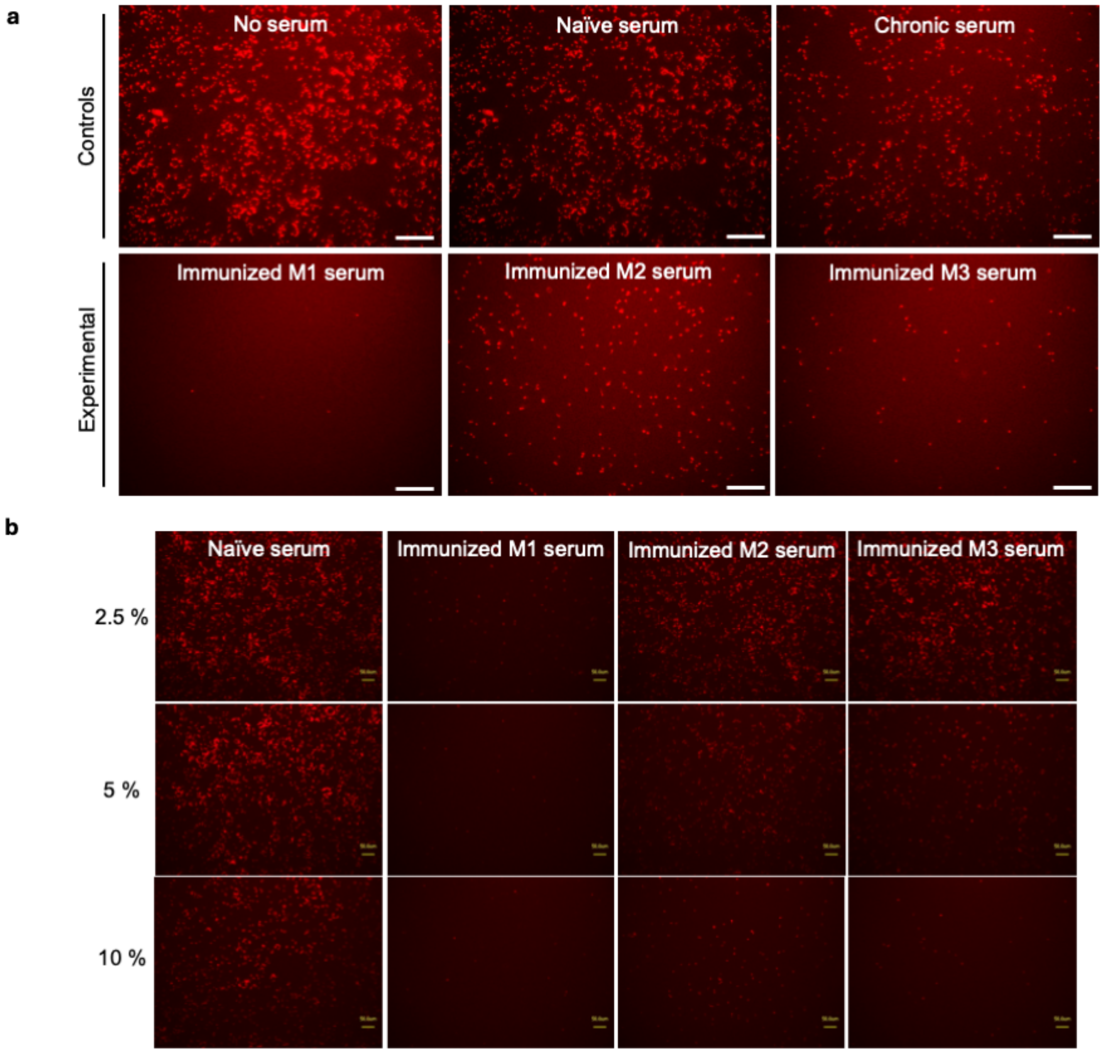

Supplementary Fig. S7 | Live antibody-mediated neutralization of trypomastigote infection of COLO-N680 cells using TcPOP antisera. a, Representative live-fluorescence images from a well containing COLO-N680 cells infected for 4 hrs with trypomastigotes. Prior to imaging, non-internalized parasites were removed by three PBS washes. Before infection, trypomastigotes were pre-incubated for 4 hrs with 10% TcPOP antisera in DMEM on an orbital shaker at 37°C (Experimental panels). Antisera were collected from three mice. Control groups included plain DMEM and DMEM containing 10% sera from naïve and chronically infected mice (>100 days post-infection). **b**, Representative live-fluorescence images, as above, from wells illustrating the effect of different sera concentrations on neutralizing trypomastigote invasion. Data were obtained from three wells per condition, representative of two independent experiments. Scale bars represent 100 μm for images in (a) and 50 μm for images in (b). Parasites (red).

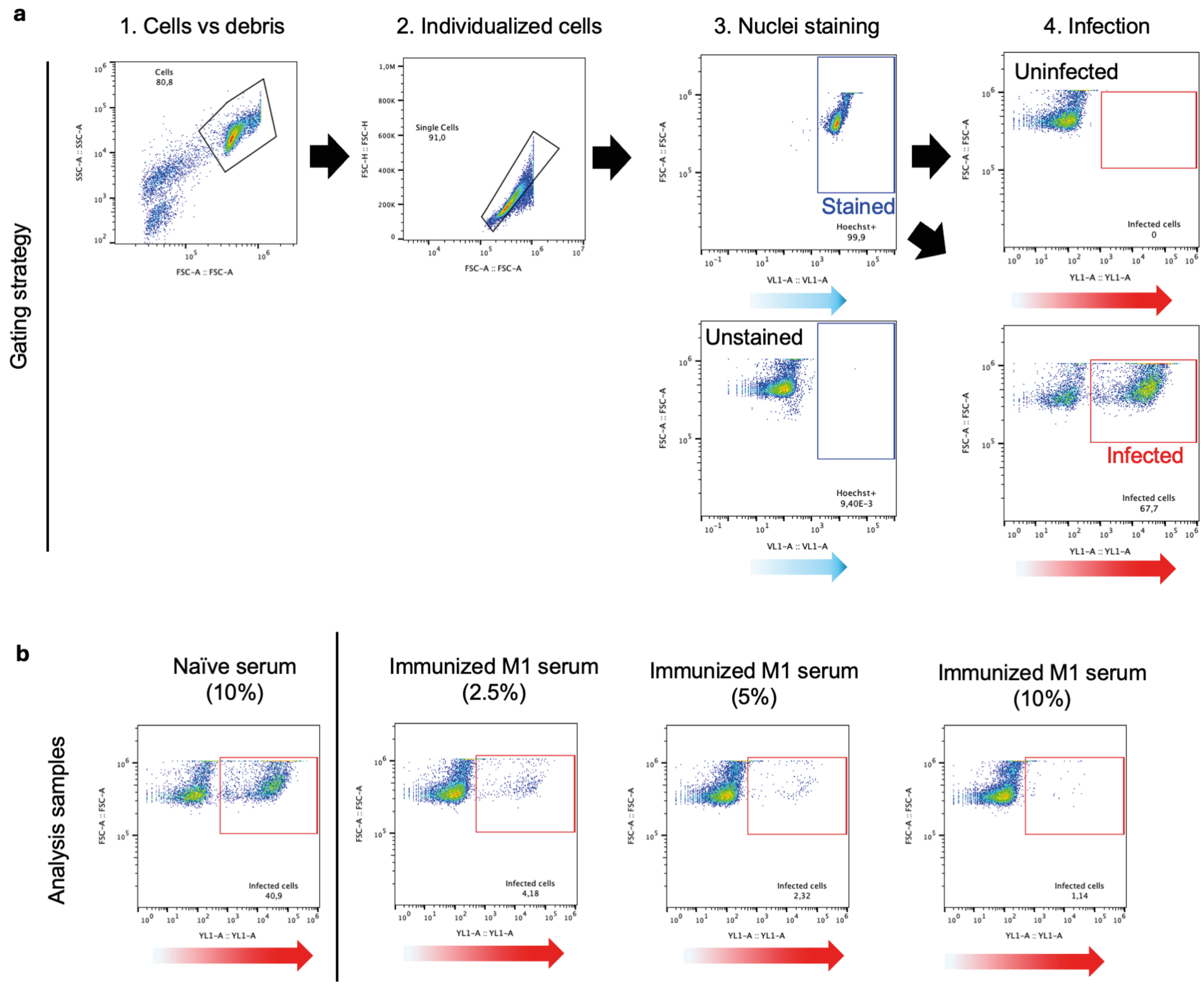

Supplementary Fig. S8 | Gating strategy for analyzing fluorescent *T. cruzi* CL-Luc:mScarlet infection of COLO-N680 cells. a, COLO-N680 cells, both infected and non-infected, were distinguished from debris and free parasites using side (S) and forward (F) scattering. Individual cells were then gated using forward scatter area (FSC-A) versus height (FSC-H). Cell gating was confirmed by DNA staining with 5 µg/ml of Hoechst 33342. Within the Hoechst-stained population, infected cells were identified by adjusting the population using a non-infected control. **b**, Representative plots of some analysed samples show infected cells gated within the red square window on the plot. Blue arrows indicate fluorescence intensity from violet laser excitation (blue emission, Hoechst), while red arrows indicate fluorescence intensity from yellow laser excitation (red emission, mScarlet).

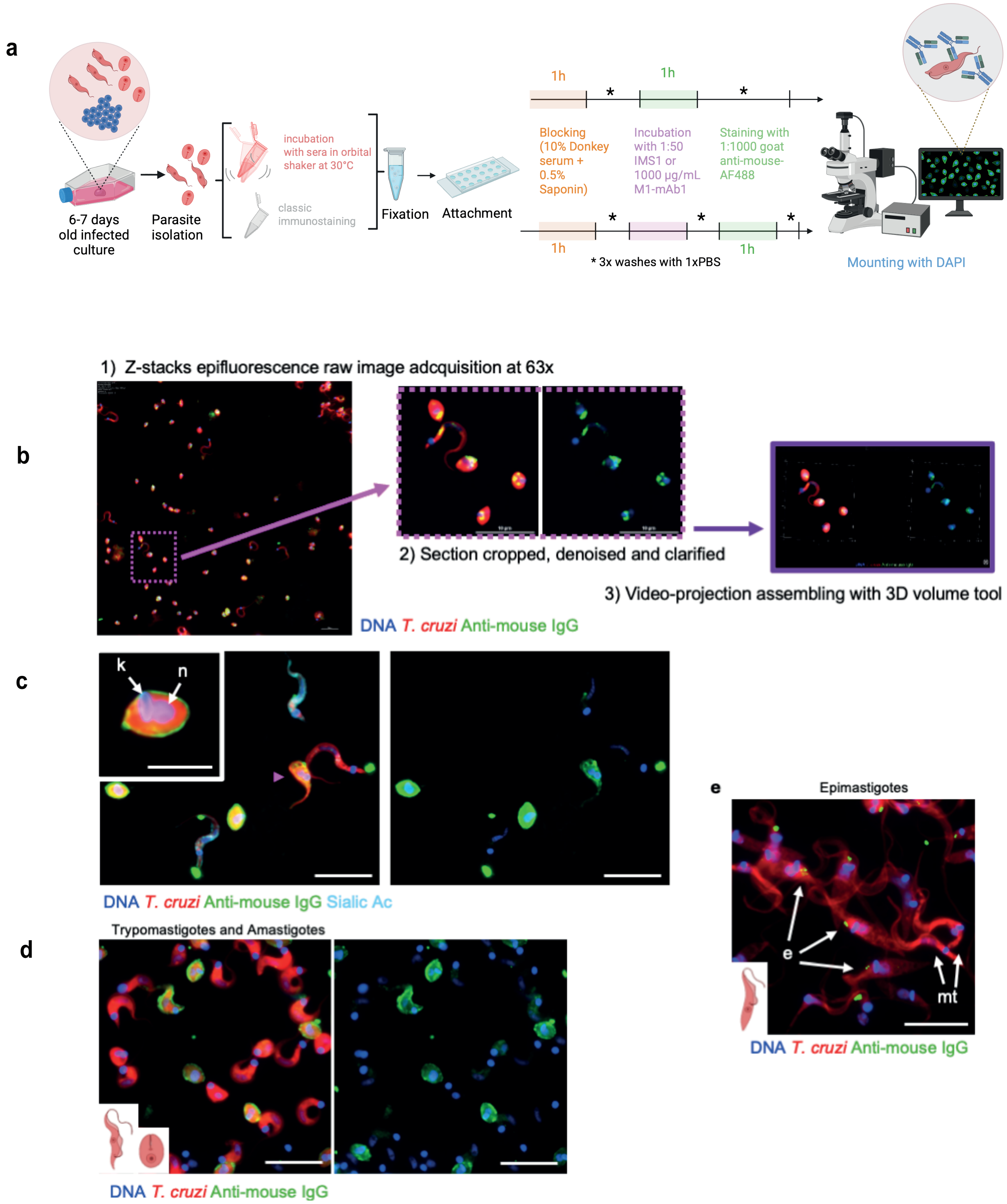

Supplementary Fig. S9 | Binding patterns of polyclonal antiserum from mouse 1 revealed by immunostaining of trypomastigotes and amastigotes. a, Schematic representation of the experimental approach for live binding and immunostaining assays. Image produced with Biorender (licence https://BioRender.com/w18l106). **b,** Generation of 3D projections from z-stack epifluorescence

images. Freshly isolated parasites were washed and maintained in DMEM for 1 hr before fixation. Subsequently, the parasites were immunostained as described in (a) and imaged using fluorescence microscopy, acquiring z-stack images with 0.3 μm slices across a 3 μm section. The images were then processed using NIS-Elements software (Nikon), and video projections were generated using the 3D volume tool (see supplementary video 2). **c**, Visualization of the binding pattern of antibodies present in 5% polyclonal antiserum from mouse 1. Live parasites were maintained in DMEM with 5% foetal bovine serum (FBS) for 48 hrs, during which endocytosis is known to be reduced in trypomastigotes and almost completely inhibited in amastigotes. After a 15-min incubation with IMS1, the image shows a trypomastigote (indicated by the pink arrowhead) that was swollen and disrupted, while trypomastigotes covered with sialic acid remained intact. Amastigotes showed homogeneous surface binding (see inset image, k = kinetoplast; n = nuclei). **d**, Illustration of endocytic competition between sera. To mitigate the lytic effect of the antiserum, freshly isolated parasites were maintained in 5% FBS, followed by the addition of 5% ISM1 for 15 min. As a result, all parasites appeared intact, with some amastigotes and trypomastigotes showing superficial binding of IgGs contained in the antiserum. **e**, Binding pattern of TcPOP antiserum in replicative and non-infective forms, specifically epimastigotes (e), correlating with the major site of endocytosis/exocytosis located at the anterior end of the parasite; and non-replicative and infective metacyclic trypomastigotes (mt) which showed no binding. Scale bars represent 10 μm for images in (b), (c), and (d), and 5 μm for the inset in (b).

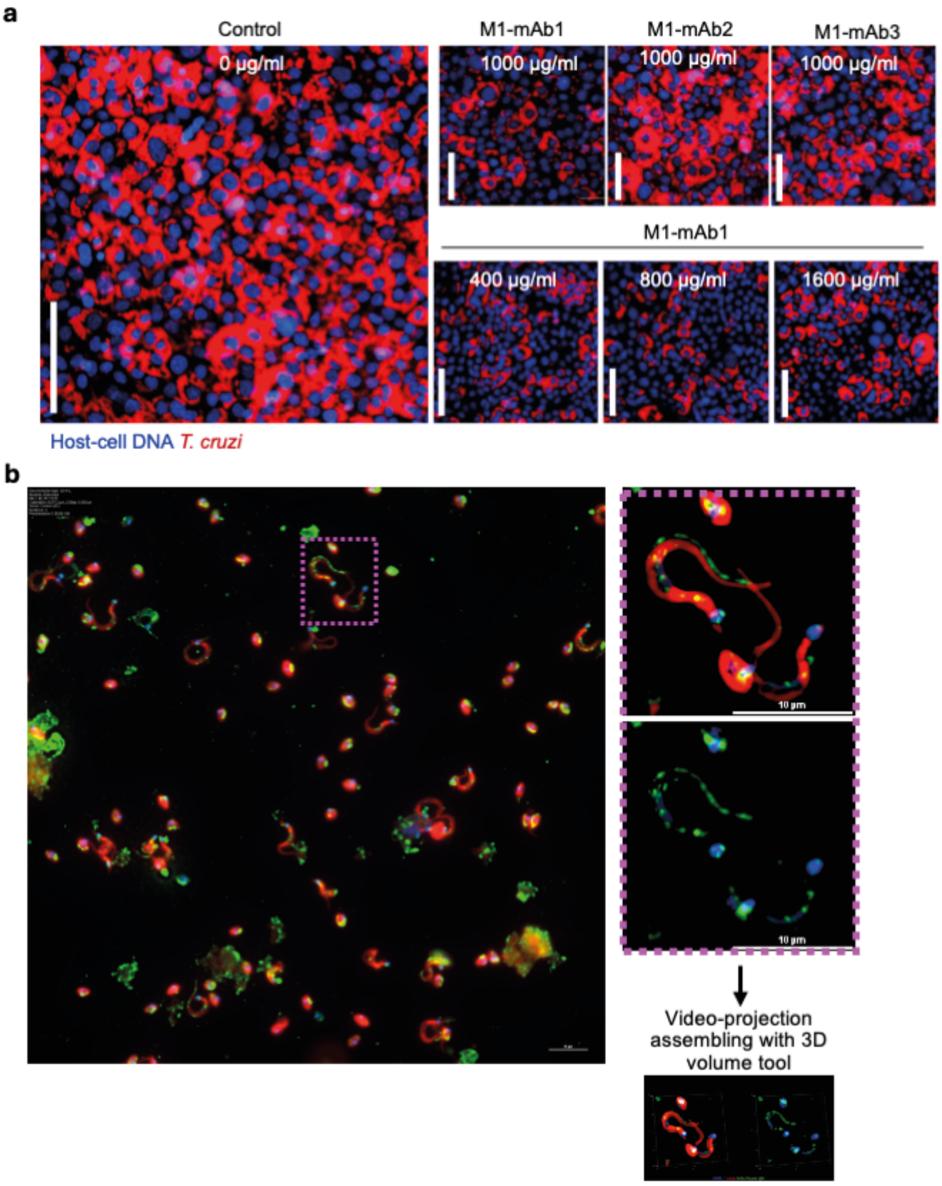

Supplementary Fig. S10 | Neutralizing and parasite binding activity of monoclonal antibodies. a, Representative images of infected cells after challenge with trypomastigotes that were previously incubated with different concentrations of the mAbs under study. The left panel shows the control assay with DMEM. The top right panels display the neutralizing activity of the three mAbs obtained from the spleen of mouse 1 (Fig. 3a), following incubation with trypomastigotes at 1000 µg/ml for 4 hrs. The bottom right panels demonstrate the inhibitory activity of mAb 1 at three different serial concentrations. **b**, Visualization of the binding pattern of mAb 1 when incubated in fixed and freshly isolated trypomastigotes and amastigotes following the immunostaining protocol shown in (Fig. S9a). The left panel presents a

representative image of a z-stack captured with fluorescence microscopy. The middle panels show a zoomed section of interest from the full field of view acquired with a 63x oil objective. The images were processed using NIS-Elements software (Nikon), and video projections were generated using the 3D volume tool (see supplementary video 3).

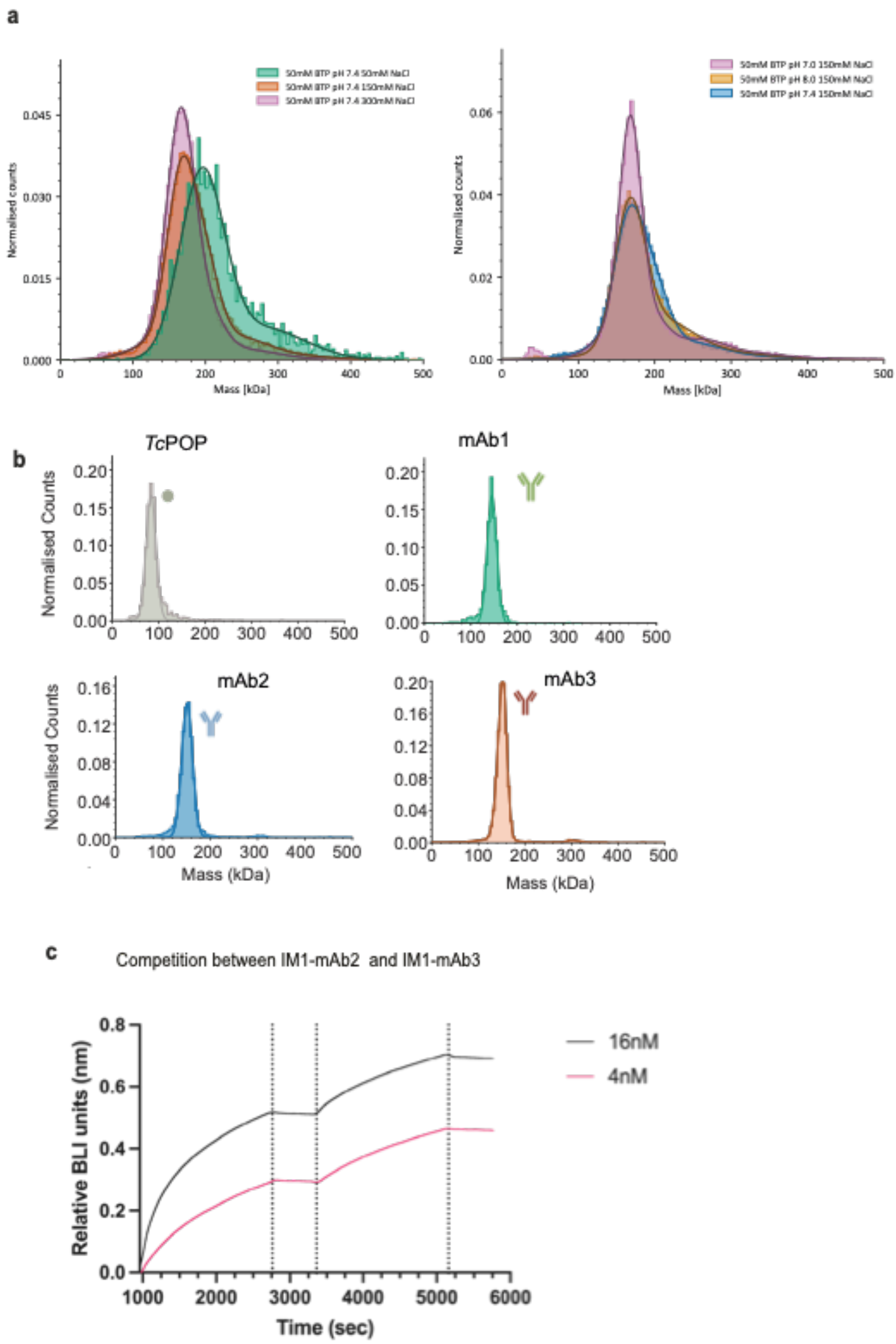

Supplementary Fig. S11 | Mass photometry measurements of TcPOP and anti-TcPOP mAbs. Mass photometry analysis of TcPOP and individual mAbs. **a**, Buffer conditions for symmetric distribution of particles were measured across different NaCl concentrations, followed by different pH, determining the conditions for optimal TcPOP stability. **b**, Mass photometry measurement of individual TcPOP and mAbs alone. **c**, Epitope binning experiments of IM1-mAb2 and IM1-mAb3 to TcPOP by biolayer interferometry (BLI). The association and dissociation of the response curves before and after addition of IM1-mAb3 to IM1-mAb2. The black and red lines represent the fitted curves at 16 nM and 4 nM concentrations.

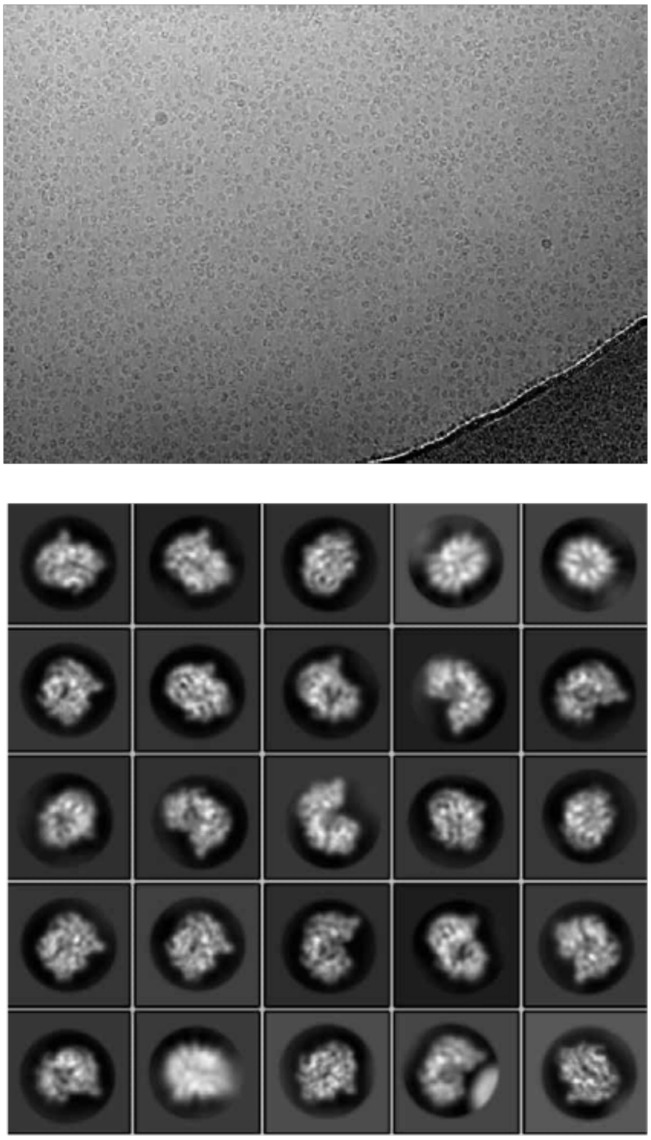

Supplementary Fig. S12 | 2D-classification of TcPOP particles. Extracted particles of TcPOP representing distinct multiple conformations from RELION5 obtained from a single grid.

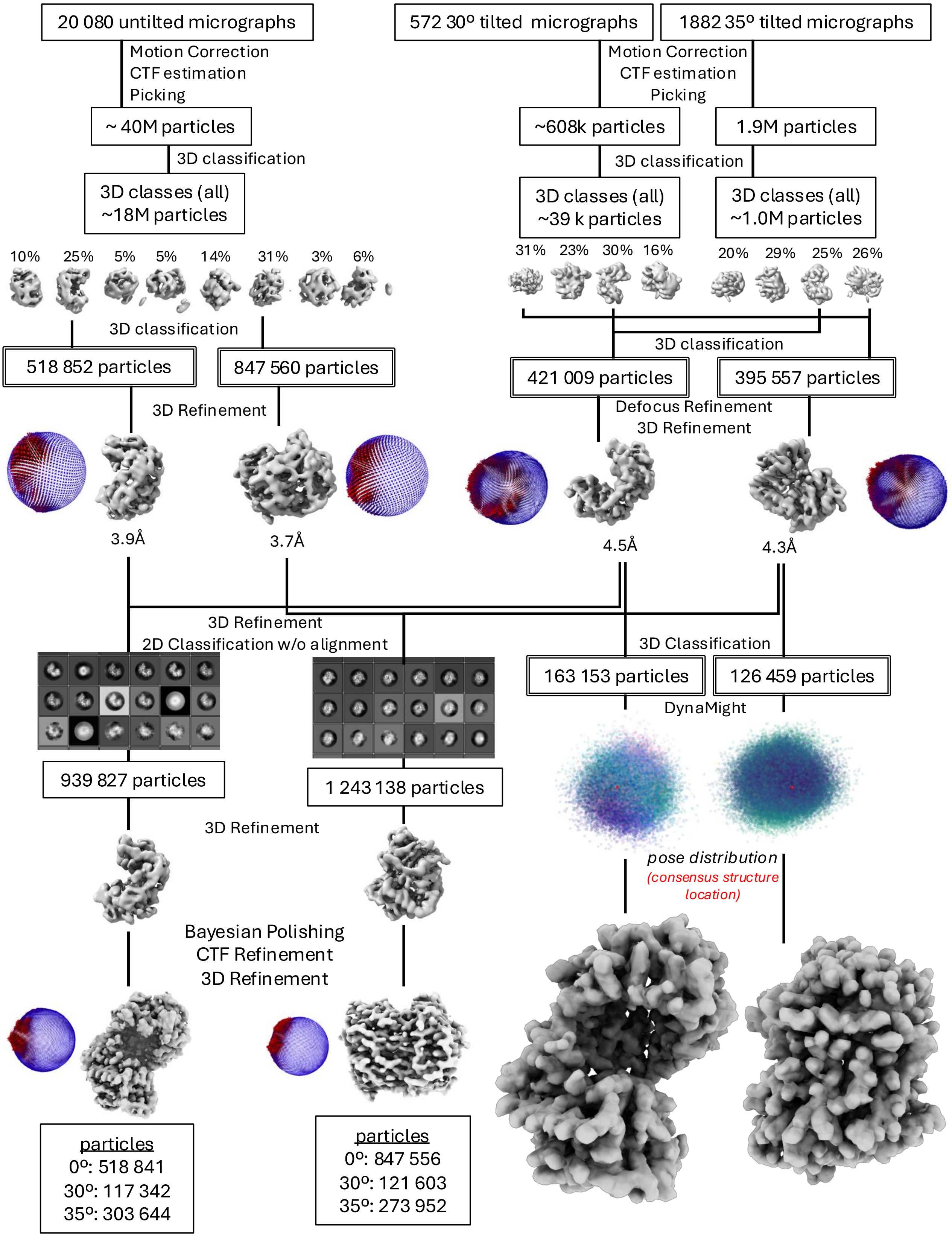

Supplementary Fig. S13 | Cryo-EM data processing workflow. The data collection strategies for untitled (left) and tilted (right) are shown step by step from data collection to electron density maps.

TcPOP open

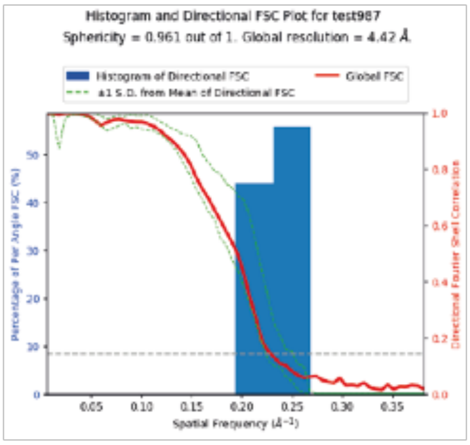

TcPOP closed

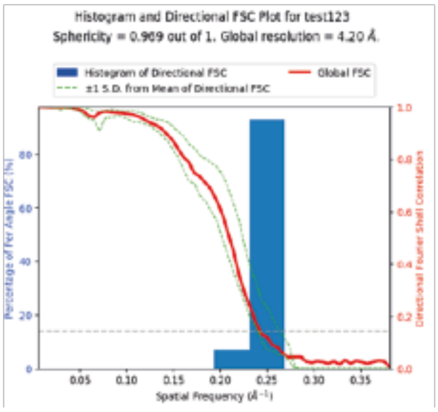

Supplementary Fig. S14 | FSC graphs of TcPOP open and TcPOP close datasets. FCS graphs calculated using the half-maps of respective closed and open conformations of TcPOP using EMBL-EBI FSC server.

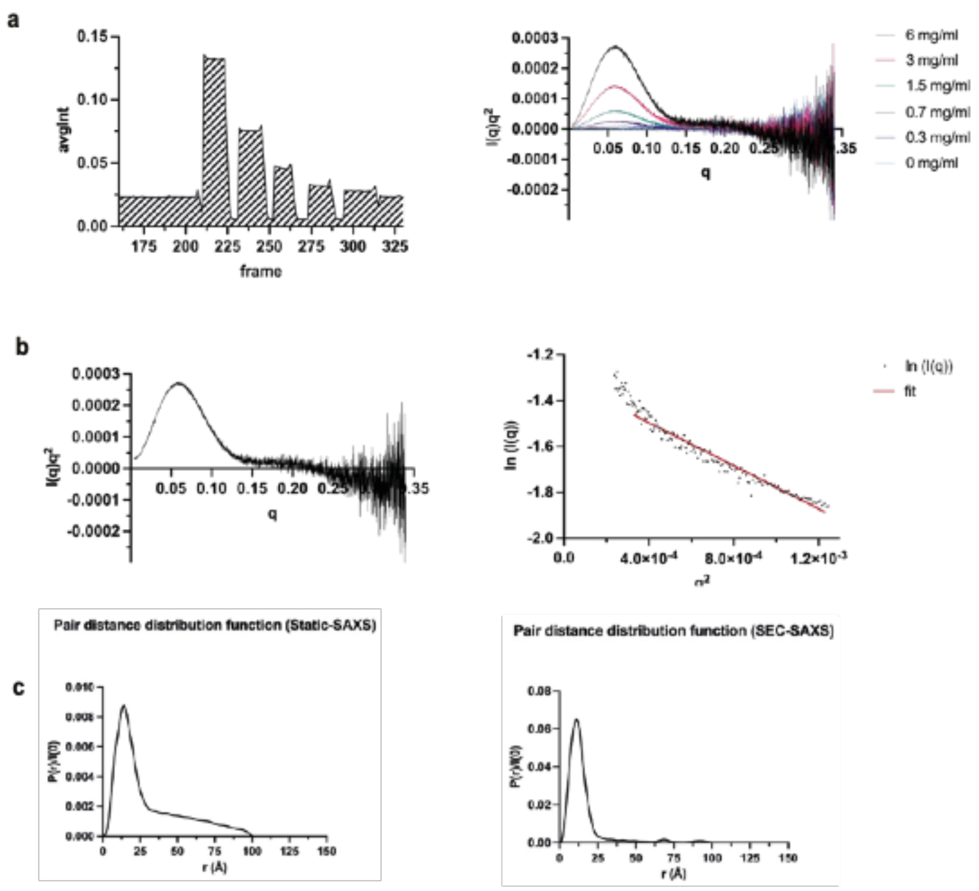

Beamline parameter	Value
Data acquisition / reduction software	GDA/DAWN
Wavelength	0.9464 Å
Energy	13.1 keV
Beam centre X	129.2 mm
Beam centre Y	19.12 mm
Sample to detector distance	3685.6 mm
Detector	EigerX 4M (Dectris)
Source	bending magnet
Flux	4x10^{12} photons/s
Q range	0.0045-0.34/Å
Q definition	4π*sin(θ)/λ
Intensity units	Per cm (absolute intensity scaled to water scatter at 0.0163)
Capillary diameter	1.5 mm
Default exposure temperature	15 °C (used unless otherwise specified per sample)
Exposure times (Seconds per frame)	1s for 21 frames (SAXS-Batch), 3s for 600 frames (SEC-SAXS)
Beam size at sample	1.0 x 0.25 mm
Beam size at detector (focus)	0.05 x 0.05 mm (FWHM)

Data	SEC-SAXS	Static SAXS
**SASDB entry**	SASDW33	SASDW23
Methods/software	BioXTAS Raw 2.3.0	BioXTAS Raw 2.3.0
**Guinier analysis**		
I(0) ± σ (SAXS)	0.03 (2.67e-5)	0.25 (9.14e-4)
Rg ± σ (Å)	30.46 (0.05)	37.62 (0.17)
qRg range	0.0045 - 0.04259	0.01807 - 0.03489
Pearson's R for fit	0.9821	0.95
**P(r) analysis**		
I(0) ± σ (SAXS, a.u.)	0.0263 (± 6.22E-05)	0.3658 (± 5.73E-04)
Rg ± σ (Å)	35.1738 (± 1.2991)	70.9772 (± 9.3054)
Dmax (Å)	189	274.1915
Q range (per Å)	0.0045 – 0.2741	0.0181 - 0.34
Chi² fit (χ2)	0.9461	0.2667

Supplementary Fig. S15 | Static SAXS of TcPOP. a, Static SAXS analysis of TcPOP at different concentrations, extrapolated to zero concentration. Samples were processed at varying concentrations, exposed to X-rays for 1 sec across 21 frames each, followed by the generation of Kratky plots. **b**, Kratky plot of TcPOP samples extrapolated to zero concentration, accompanied by the Guinier analysis of the extrapolated data, demonstrating the fit. **c** Pair distance distribution function for static-SAXS and SEC-SAXS data.

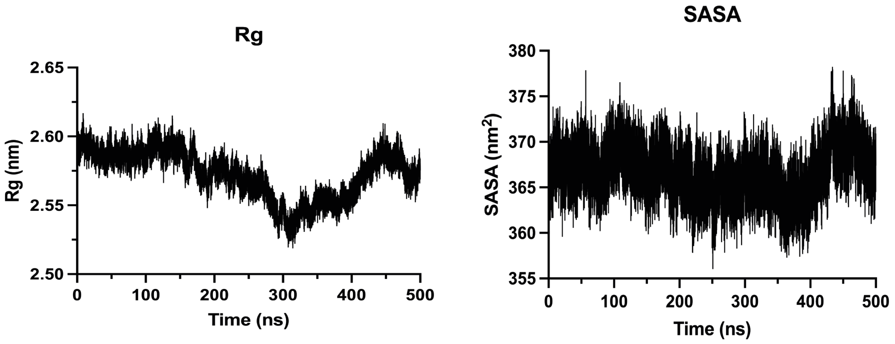

Supplementary Fig. S16 | Molecular Dynamics of TcPOP. Radius of gyration (Rg) and solvent accessible surface area (SASA) during the 500 ns simulation run performed with GROMACS.

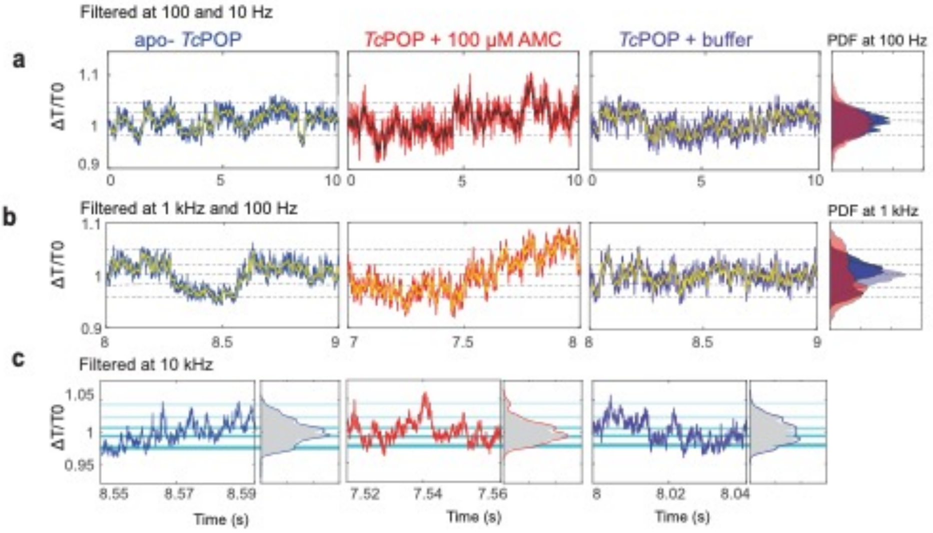

Supplementary Fig. S17 | Conformational dynamics of TcPOP in solution by optical tweezers. **a**, Time traces of transmission change (ΔT/T0) for a single TcPOP trapped in the nanoaperture before introducing substrate (blue traces), with 100 μM AMC peptide (red traces) and after introducing buffer (purple trace), along with their probability density functions (PDF). **b**, 1 sec magnified trace from panel A, and their corresponding PDFs. **c** 40 msec magnified traces of panel B with PDFs on the right of each trace. Coloured bands guide signal levels aligned with PDF peaks, each indicating a relative stable conformation.

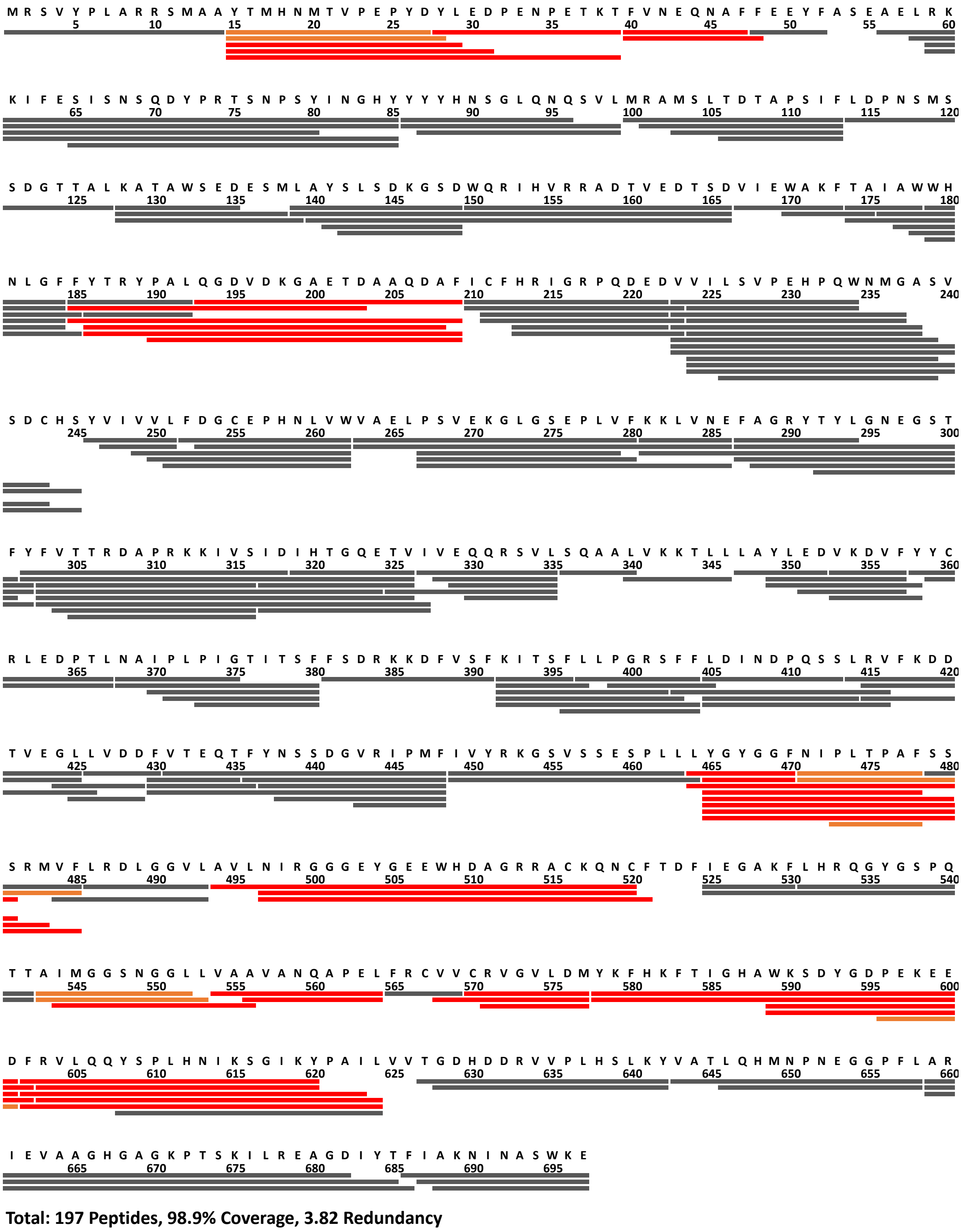

Supplementary Fig. S18 | Peptides whose HDX was followed are shown along the sequence of TcPOP. In grey: peptides exhibiting EX2 kinetics (unimodal envelope distributions); in red: peptides exhibiting EX1 or EXx kinetics (bimodal envelope distributions); in orange: peptides exhibiting EX2 kinetics (with an evident broad envelope distribution but not a defined separation between the low- and high-mass envelopes). Source data are provided as Source Data File.

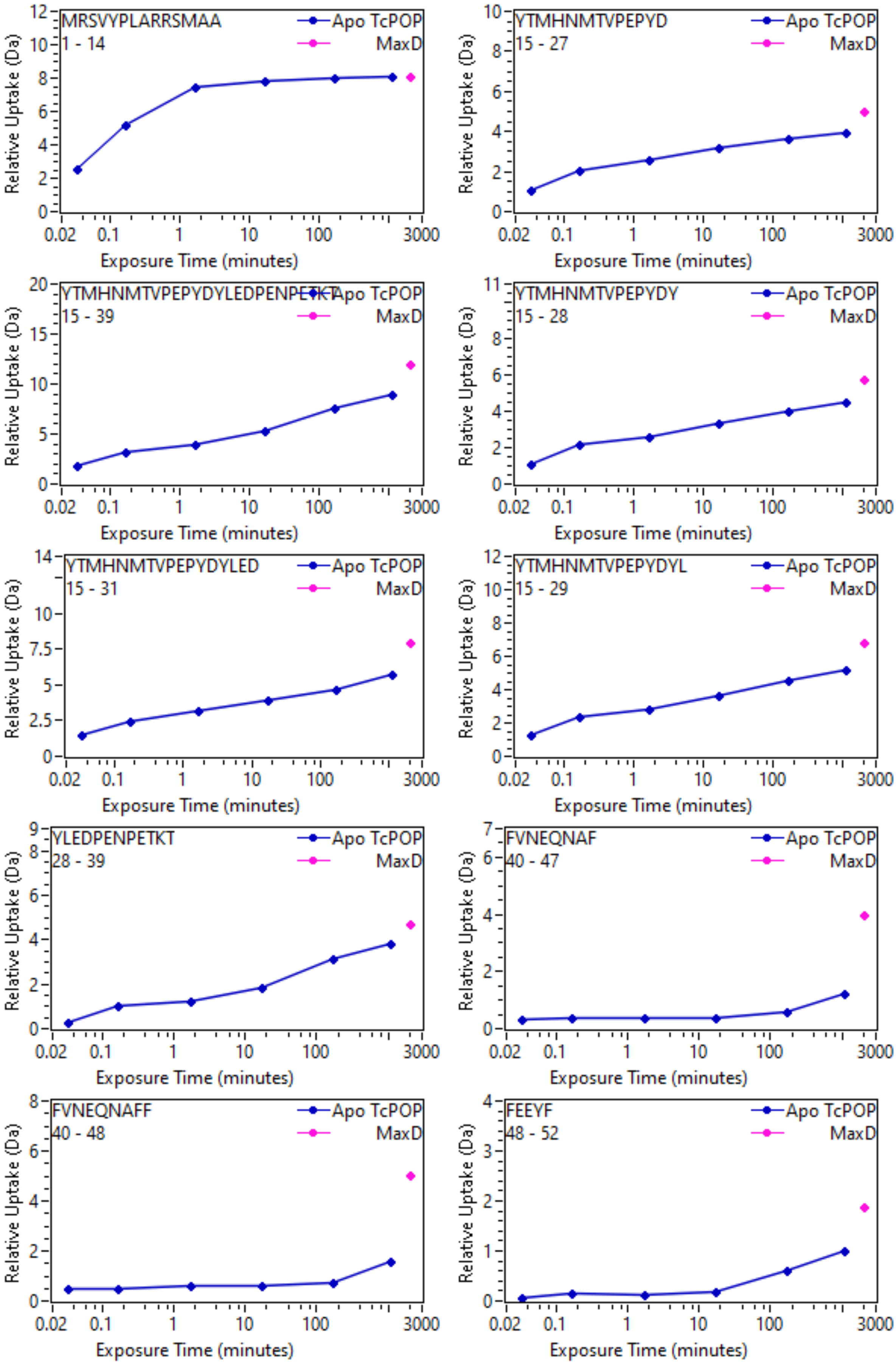
MRSVYPLARRSMAA
1 - 14
YTMHNMTVPEPYD
15 - 27
YTMHNMTVPEPYDYLEDPENPETKT
15 - 39
YTMHNMTVPEPYDY
15 - 28
YTMHNMTVPEPYDYLED
15 - 31
YTMHNMTVPEPYDYL
15 - 29
YLEDPENPETKT
28 - 39
FVNEQNAF
40 - 47
FVNEQNAFF
40 - 48
FEEYF
48 - 52
Apo TcPOP
MaxD
Relative Uptake (Da)
Exposure Time (minutes)

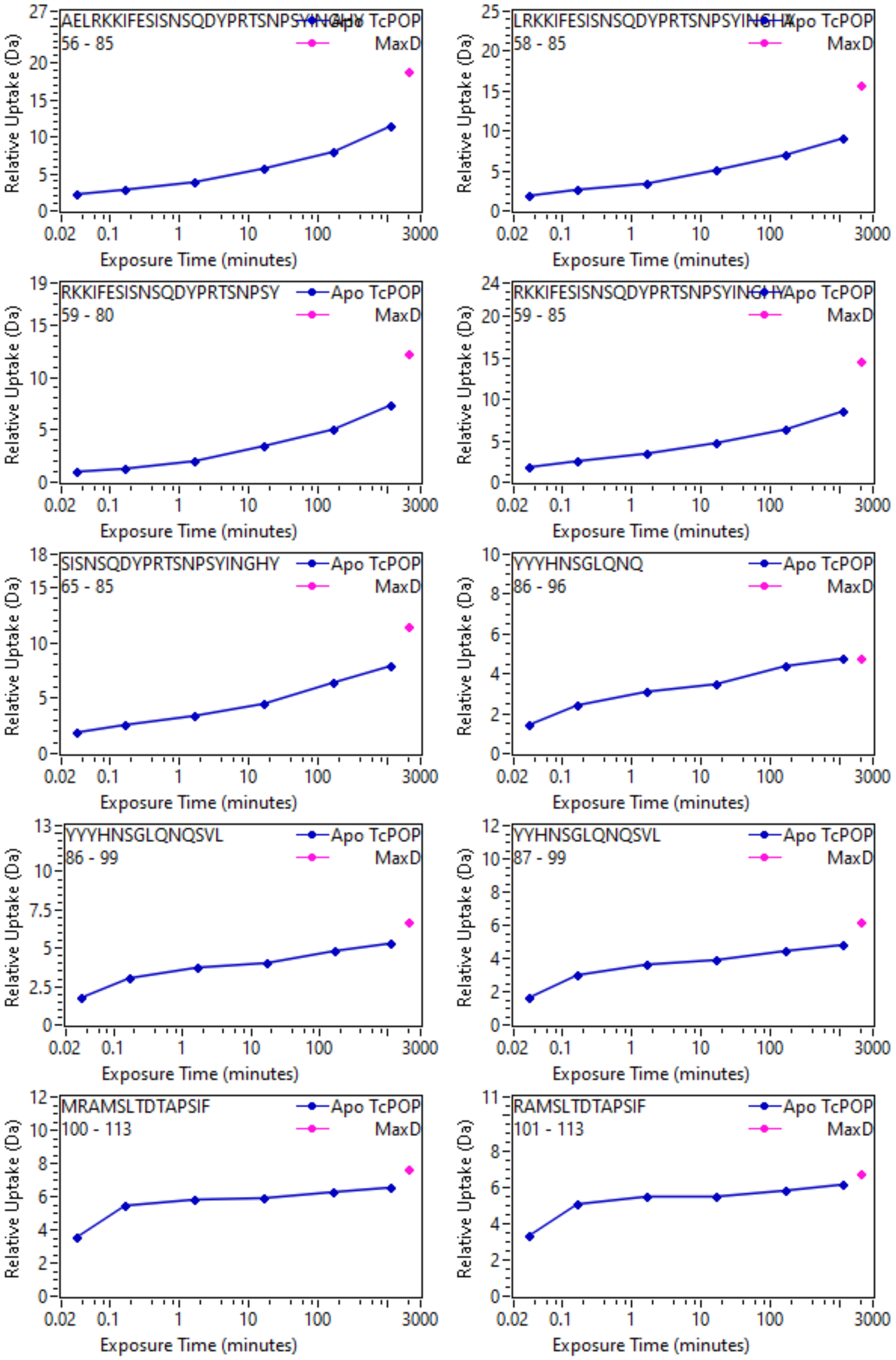
AELRKKIFESISNSQDYPRTSNPSY
56 - 85
Apo TcPOP
MaxD
LRKKIFESISNSQDYPRTSNPSYIN
58 - 85
RKKIFESISNSQDYPRTSNPSY
59 - 80
RKKIFESISNSQDYPRTSNPSYIN
59 - 85
SISNSQDYPRTSNPSYINGHY
65 - 85
YYYHNSGLQNQ
86 - 96
YYYHNSGLQNQSVL
86 - 99
YYHNSGLQNQSVL
87 - 99
MRAMSLTDTAPSIF
100 - 113
RAMSLTDTAPSIF
101 - 113
Relative Uptake (Da)
Exposure Time (minutes)
0.02
0.1
1
10
100
3000

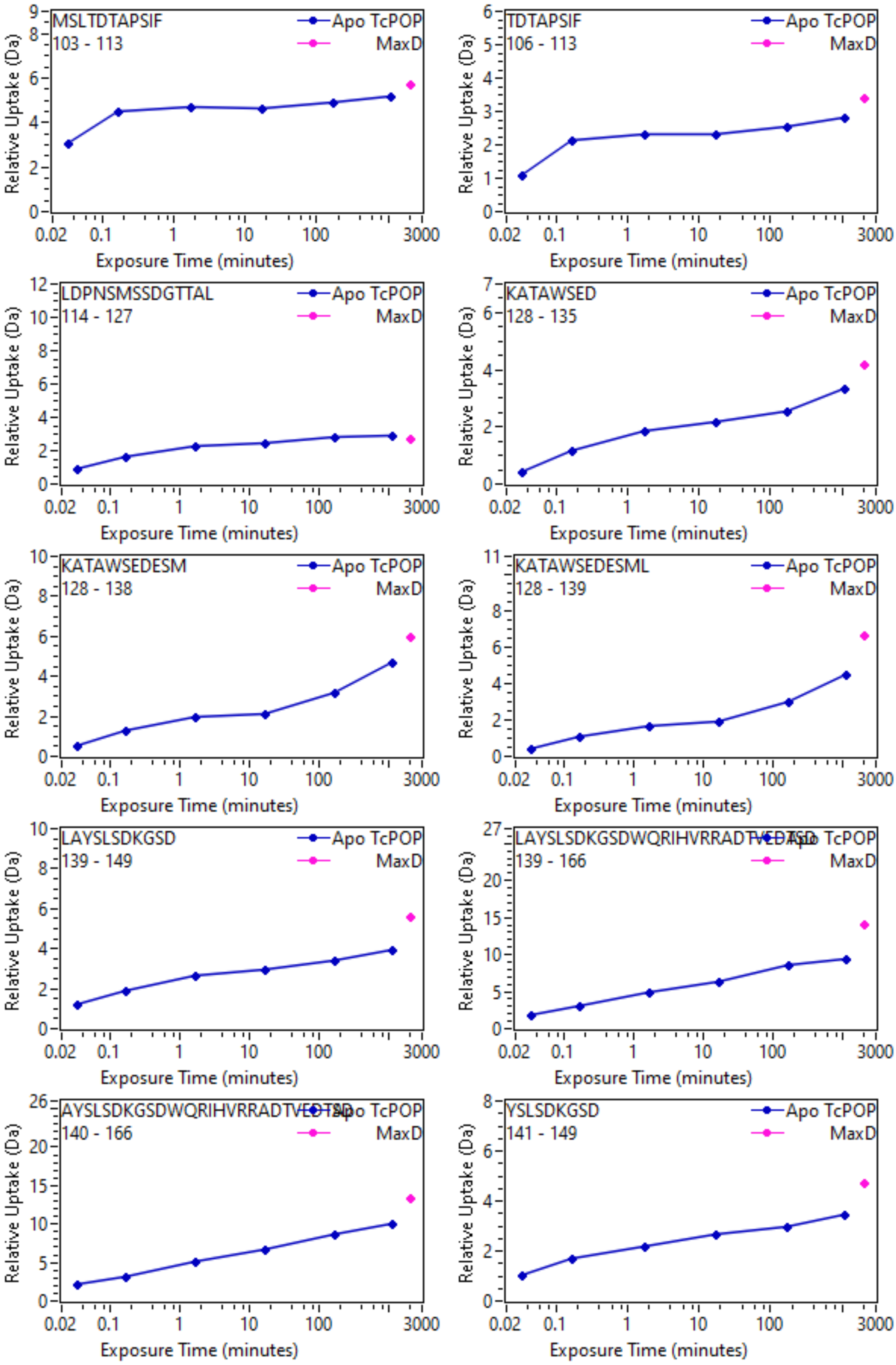
MSLTDTAPSIF
103 - 113
TDTAPSIF
106 - 113
LDPNSMSSDGTTAL
114 - 127
KATAWSED
128 - 135
KATAWSEDESM
128 - 138
KATAWSEDESML
128 - 139
LAYSLSDKGSD
139 - 149
139 - 166
140 - 166
YSLSDKGSD
141 - 149
Apo TcPOP
MaxD
Relative Uptake (Da)
Exposure Time (minutes)
0.02
0.1
1
10
100
3000

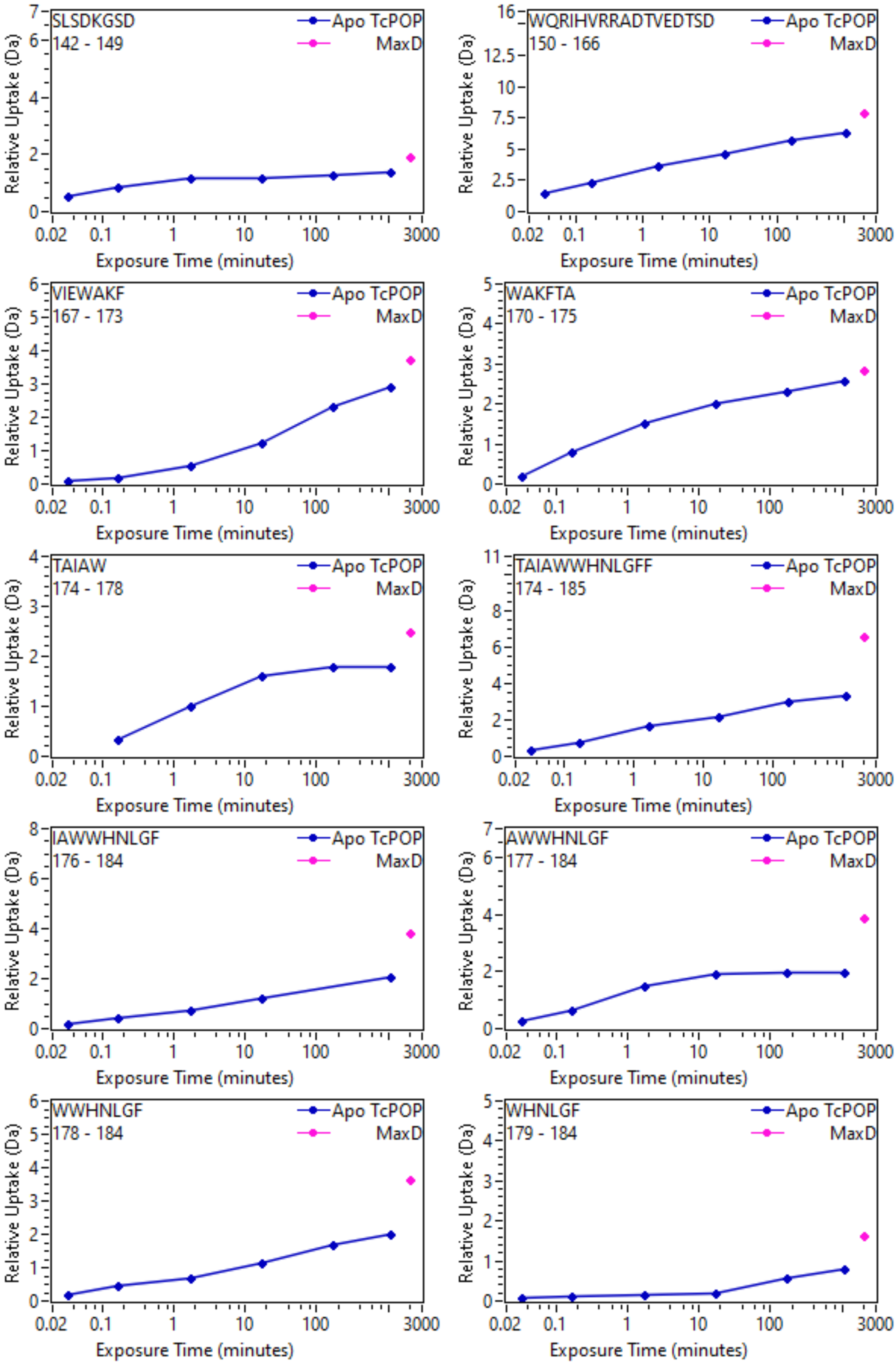
SLSDKGSD
142 - 149
WQRIHVRRADTVEDTSD
150 - 166
VIEWAKF
167 - 173
WAKFTA
170 - 175
TAIAW
174 - 178
TAIAWWHNLGFF
174 - 185
IAWWHNLGF
176 - 184
AWWHNLGF
177 - 184
WWHNLGF
178 - 184
WHNLGF
179 - 184
Apo TcPOP
MaxD
Relative Uptake (Da)
Exposure Time (minutes)
0.02
0.1
1
10
100
3000

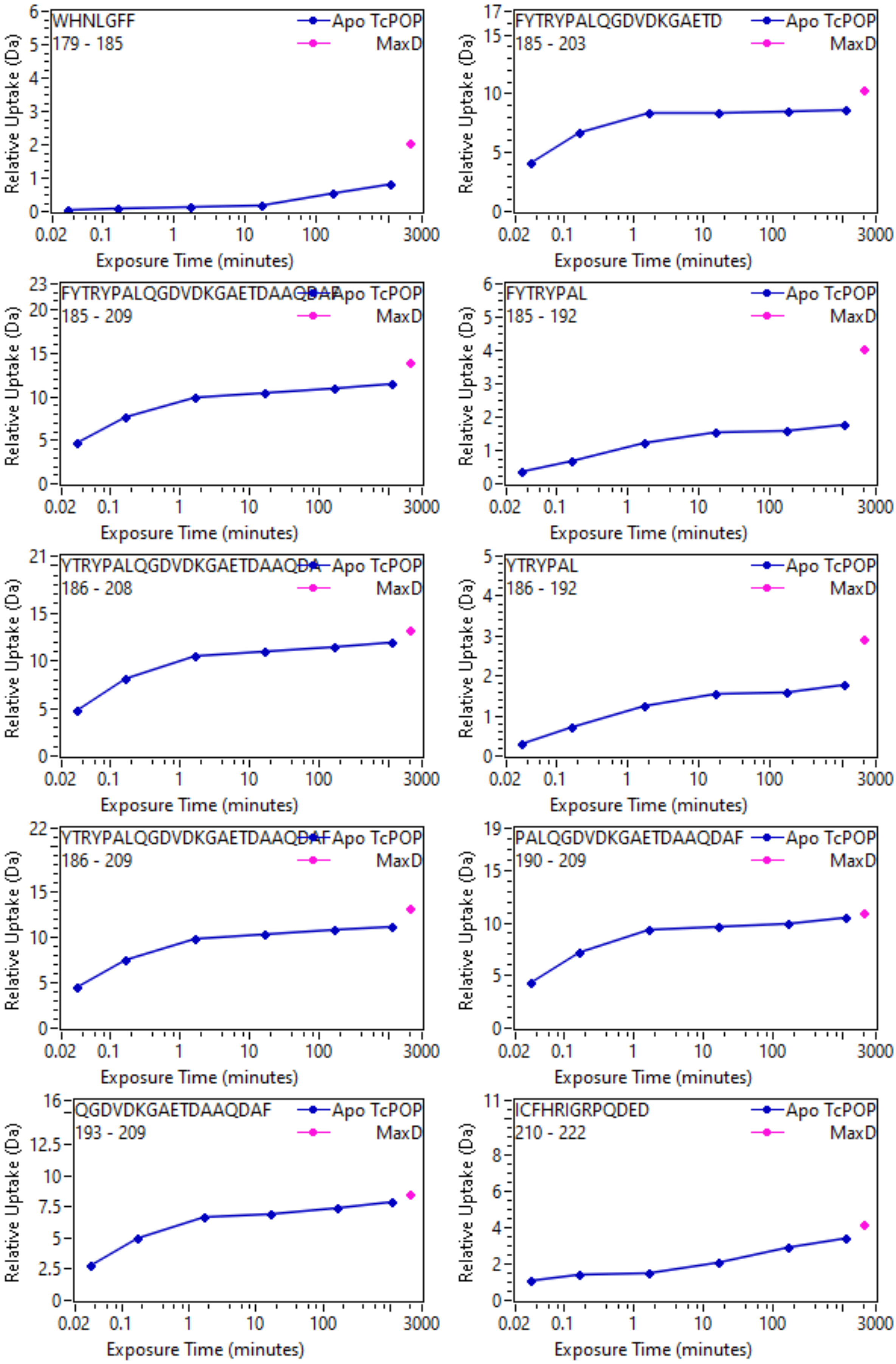

WHNLGFF
179 - 185
FYTRYPALQGDVDKGAETD
185 - 203
FYTRYPALQGDVDKGAETDAAQDAF
185 - 209
FYTRYPAL
185 - 192
YTRYPALQGDVDKGAETDAAQDA
186 - 208
YTRYPAL
186 - 192
YTRYPALQGDVDKGAETDAAQDAF
186 - 209
PALQGDVDKGAETDAAQDAF
190 - 209
QGDVDKGAETDAAQDAF
193 - 209
ICFHRIGRPQDED
210 - 222
Apo TcPOP
MaxD
Relative Uptake (Da)
Exposure Time (minutes)
0.02
0.1
1
10
100
3000

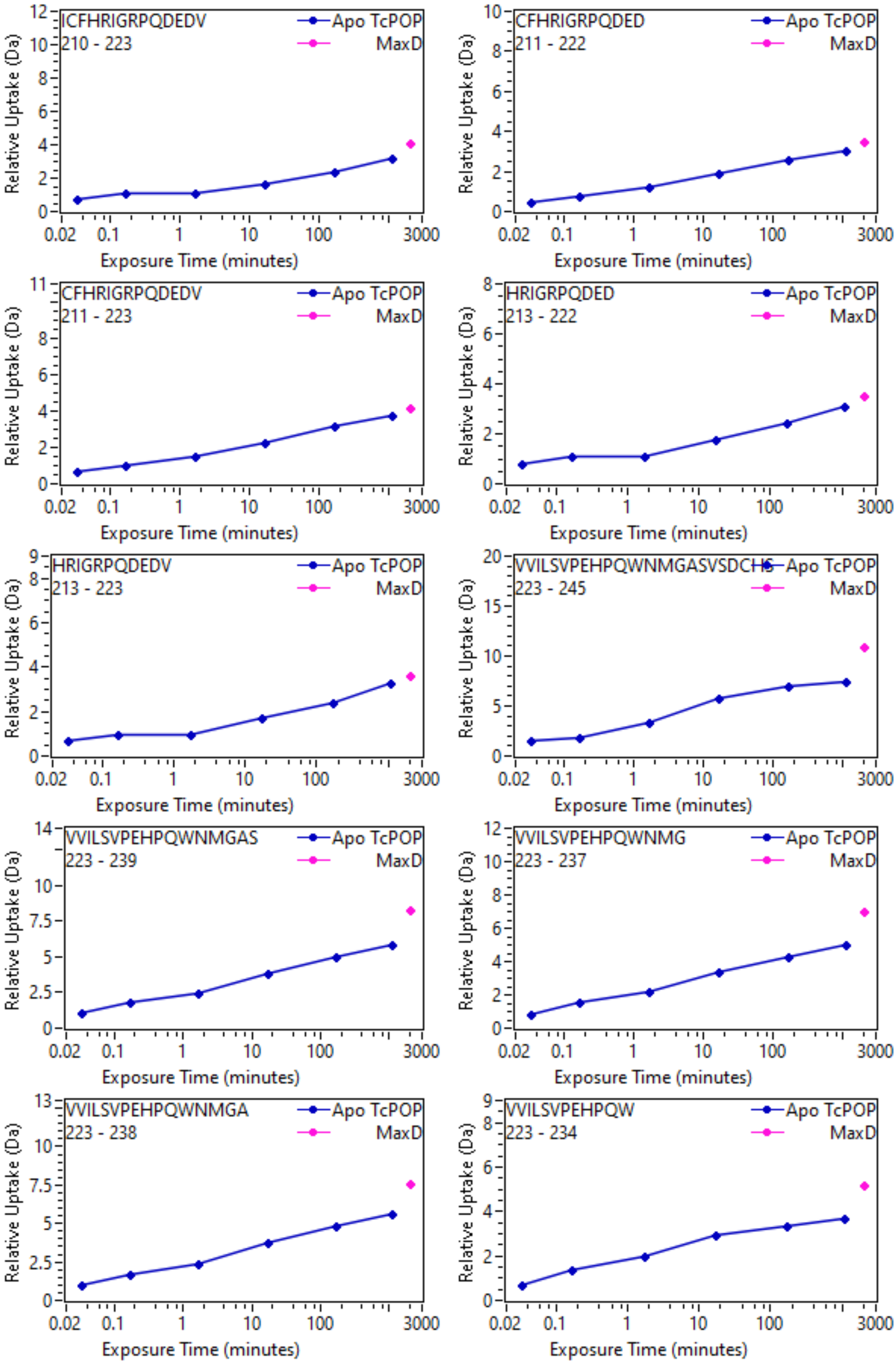
ICFHRIGRPQDEDV
210 - 223
CFHRIGRPQDED
211 - 222
CFHRIGRPQDEDV
211 - 223
HRIGRPQDED
213 - 222
HRIGRPQDEDV
213 - 223
VVILSVPEHPQWNMGASVSDCHS
223 - 245
VVILSVPEHPQWNMGAS
223 - 239
VVILSVPEHPQWNMG
223 - 237
VVILSVPEHPQWNMGA
223 - 238
VVILSVPEHPQW
223 - 234
Apo TcPOP
MaxD
Relative Uptake (Da)
Exposure Time (minutes)
0.02
0.1
1
10
100
3000

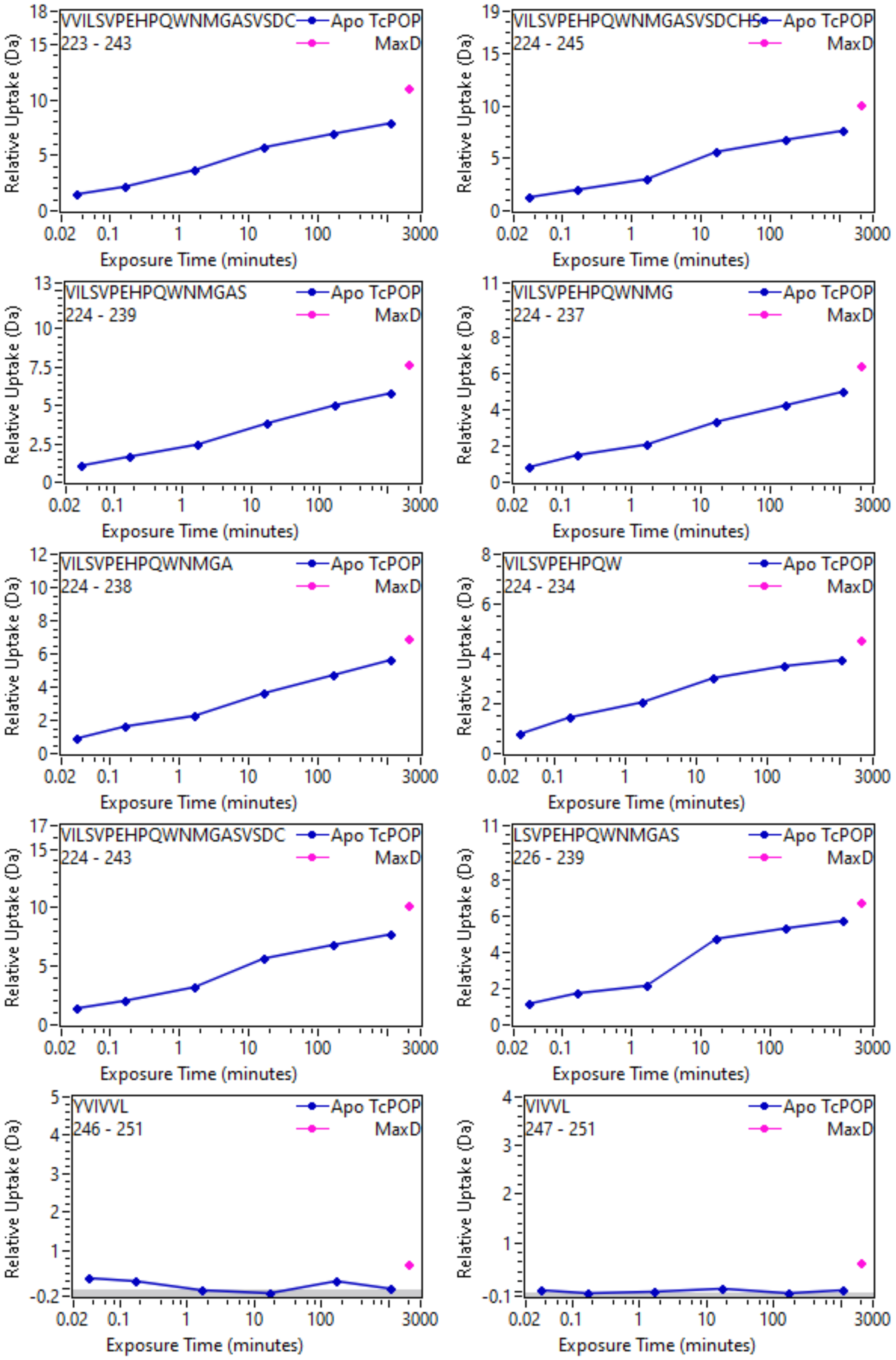

VVILSVPEHPQWNMGASVSDC
223 - 243
VILSVPEHPQWNMGASVSDCHS
224 - 245
VILSVPEHPQWNMGAS
224 - 239
VILSVPEHPQWNMG
224 - 237
VILSVPEHPQWNMGA
224 - 238
VILSVPEHPQW
224 - 234
VILSVPEHPQWNMGASVSDC
224 - 243
LSVPEHPQWNMGAS
226 - 239
YVIVVL
246 - 251
VIVVL
247 - 251
Apo TcPOP
MaxD
Relative Uptake (Da)
Exposure Time (minutes)
0.02
0.1
1
10
100
3000

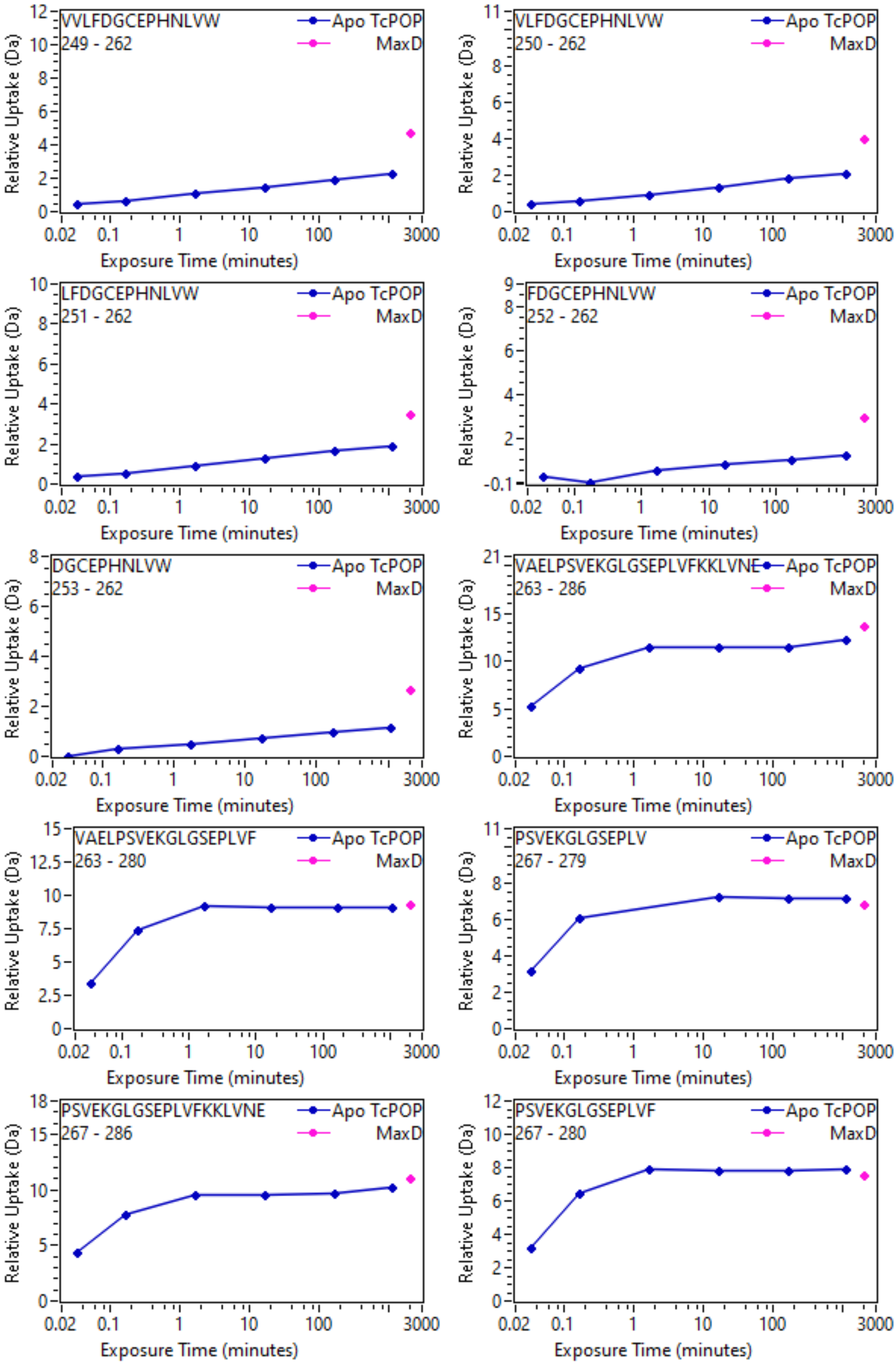
VVLFDGCEPHNLVW
249 - 262
VLFDGCEPHNLVW
250 - 262
LFDGCEPHNLVW
251 - 262
FDGCEPHNLVW
252 - 262
DGCEPHNLVW
253 - 262
VAELPSVEKGLGSEPLVFKKLVNE
263 - 286
VAELPSVEKGLGSEPLVF
263 - 280
PSVEKGLGSEPLV
267 - 279
PSVEKGLGSEPLVFKKLVNE
267 - 286
PSVEKGLGSEPLVF
267 - 280
Apo TcPOP
MaxD
Relative Uptake (Da)
Exposure Time (minutes)
0.02
0.1
1
10
100
3000

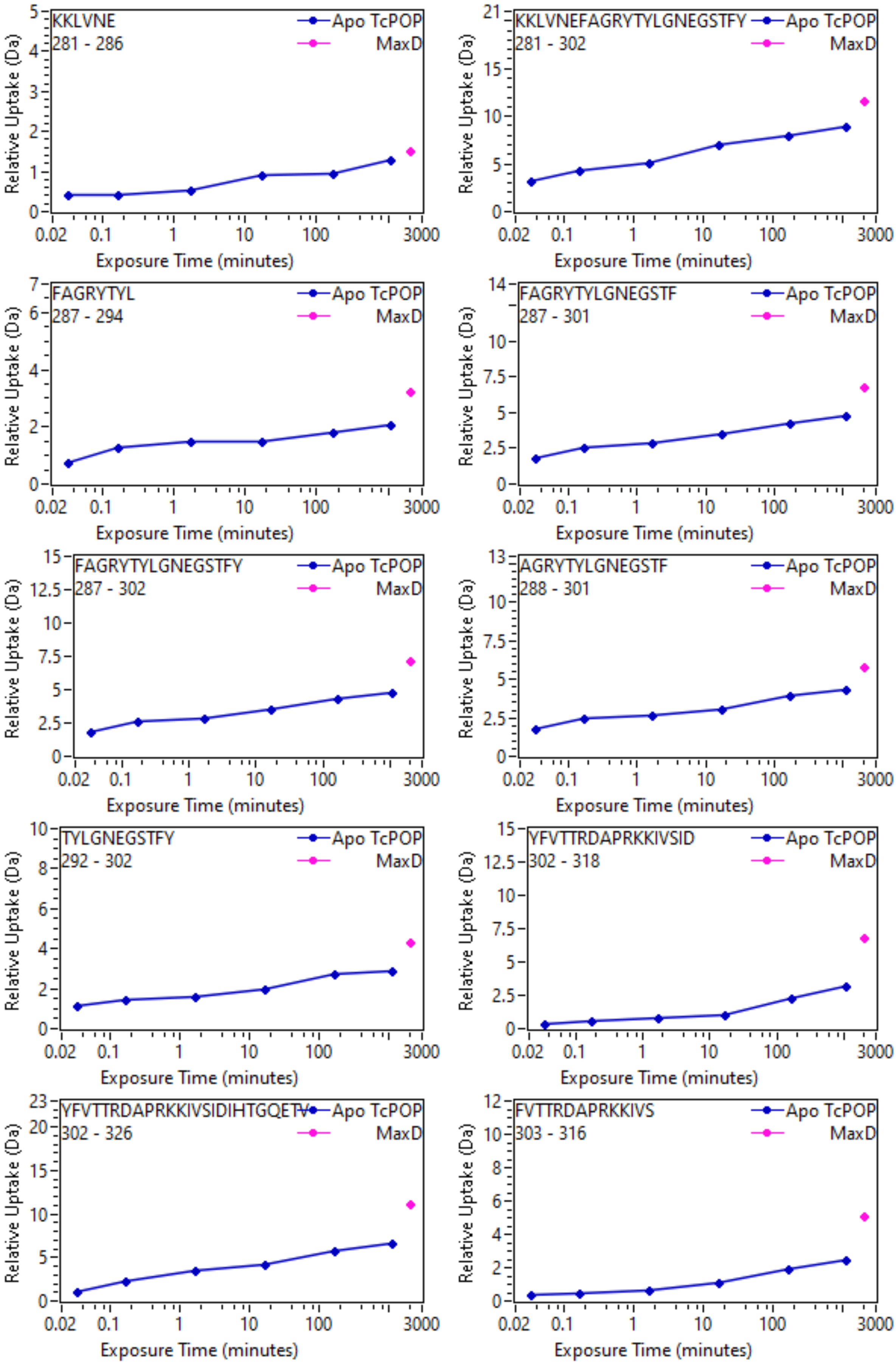
KKLVNE
281 - 286
KKLVNEFAGRYTYLGNEGSTFY
281 - 302
FAGRYTYL
287 - 294
FAGRYTYLGNEGSTF
287 - 301
FAGRYTYLGNEGSTFY
287 - 302
AGRYTYLGNEGSTF
288 - 301
TYLGNEGSTFY
292 - 302
YFVTTRDAPRKKIVSID
302 - 318
YFVTTRDAPRKKIVSIDIHTGQETV
302 - 326
FVTTRDAPRKKIVS
303 - 316
Apo TcPOP
MaxD
Relative Uptake (Da)
Exposure Time (minutes)
0.02
0.1
1
10
100
3000

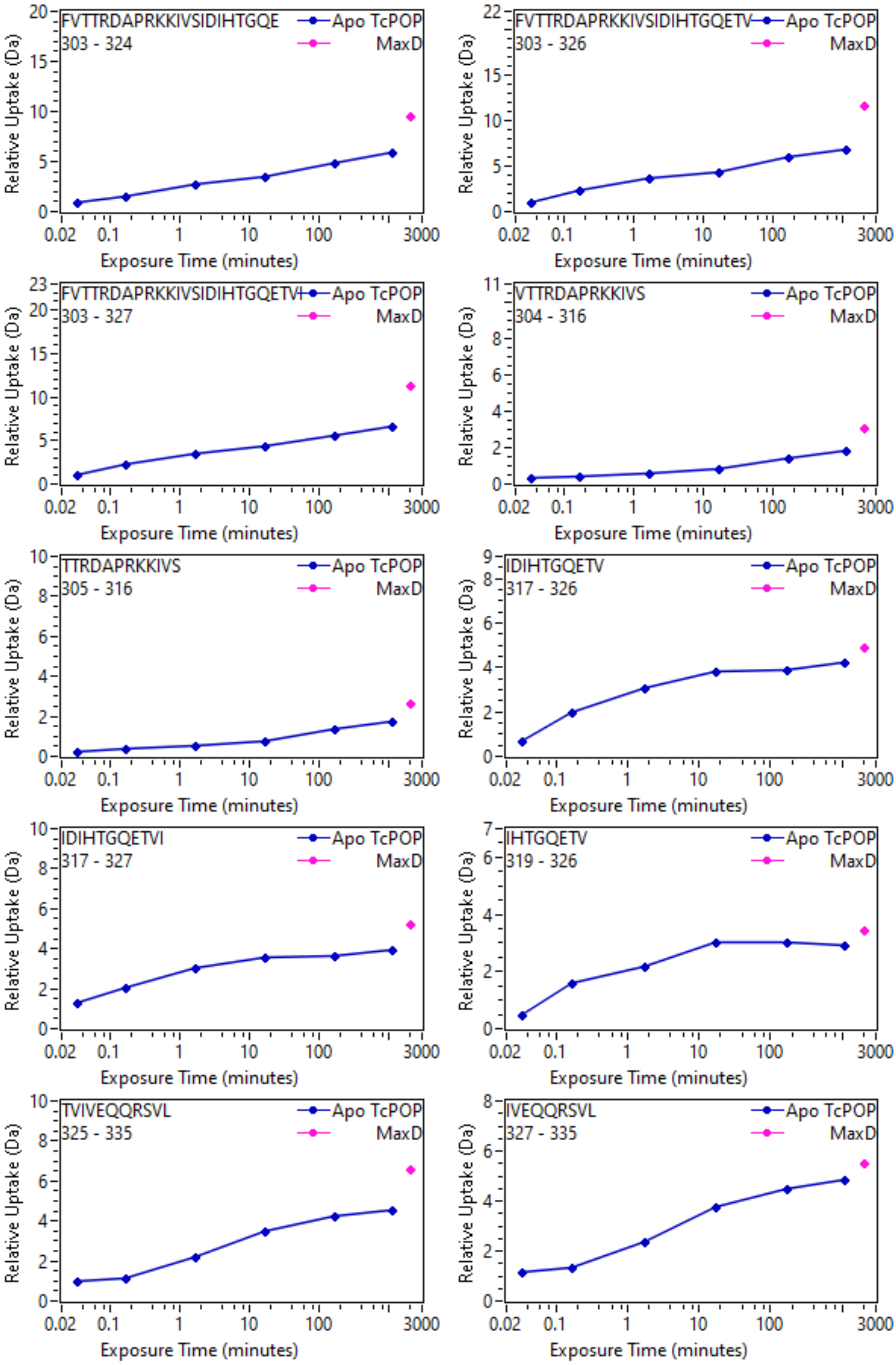
FVTTRDAPRKKIVSIDIHTGQE
303 - 324
FVTTRDAPRKKIVSIDIHTGQETV
303 - 326
FVTTRDAPRKKIVSIDIHTGQETVI
303 - 327
VTTRDAPRKKIVS
304 - 316
TTRDAPRKKIVS
305 - 316
IDIHTGQETV
317 - 326
IDIHTGQETVI
317 - 327
IHTGQETV
319 - 326
TVIVEQQRSVL
325 - 335
IVEQQRSVL
327 - 335
Apo TcPOP
MaxD
Relative Uptake (Da)
Exposure Time (minutes)
0.02
0.1
1
10
100
3000

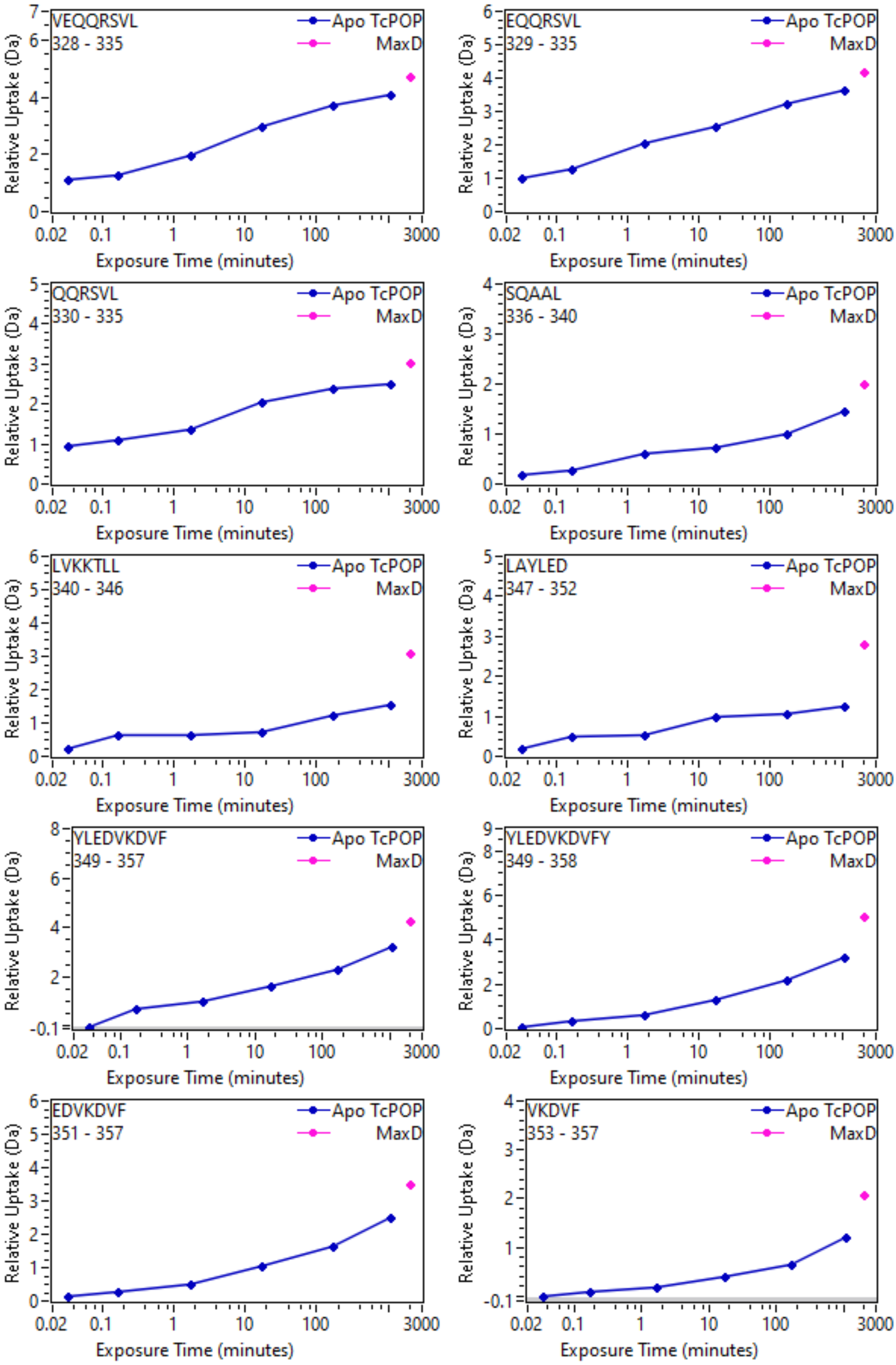
VEQQRSVL
328 - 335
EQQRSVL
329 - 335
QQRSVL
330 - 335
SQAAL
336 - 340
LVKKTLL
340 - 346
LAYLED
347 - 352
YLEDVKDVF
349 - 357
YLEDVKDVFY
349 - 358
EDVKDVF
351 - 357
VKDVF
353 - 357
Apo TcPOP
MaxD
Relative Uptake (Da)
Exposure Time (minutes)
0.02
0.1
1
10
100
3000

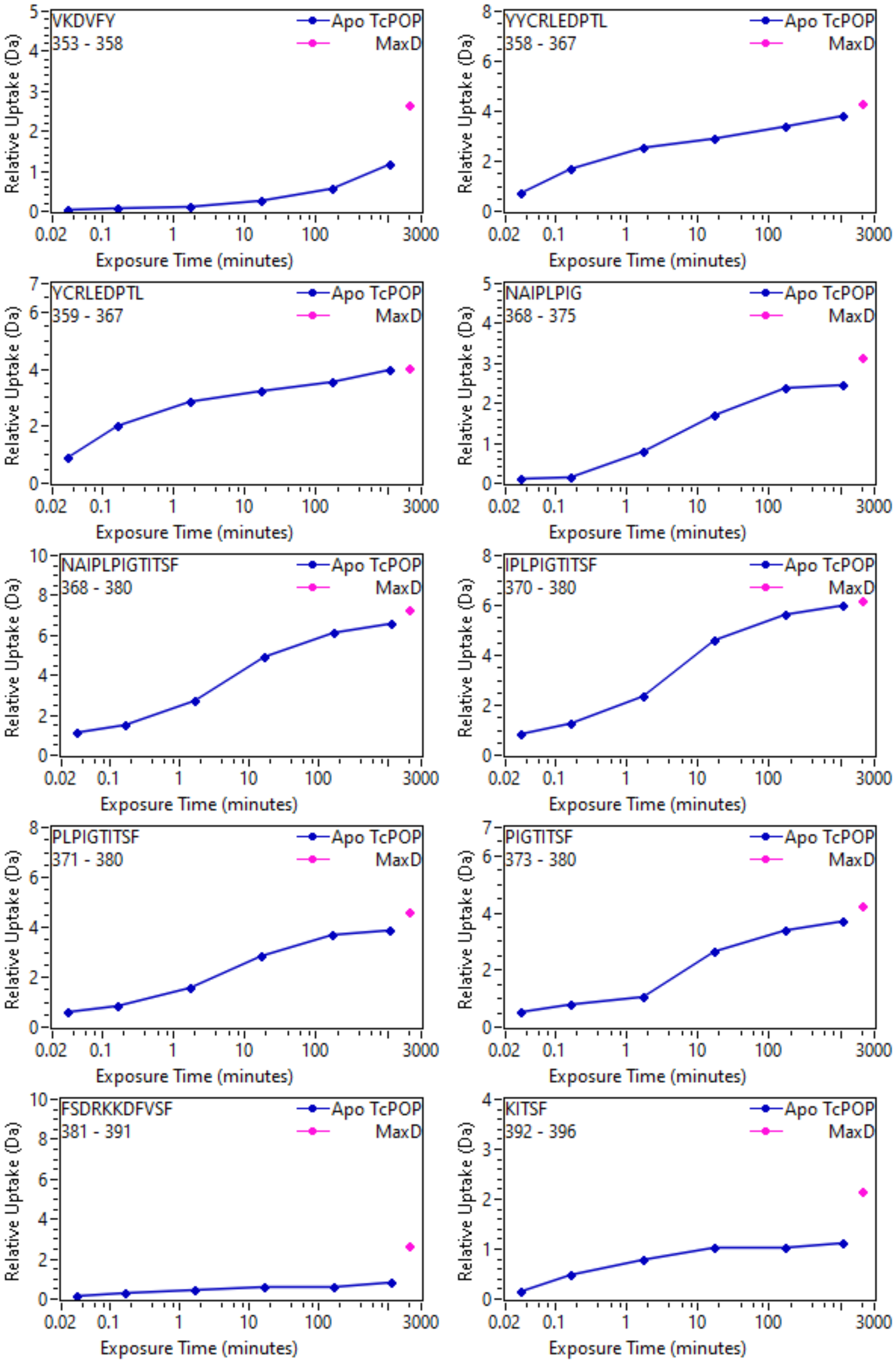

VKDVFY
353 - 358
Apo TcPOP
MaxD
Relative Uptake (Da)
Exposure Time (minutes)
YYCRLEDPTL
358 - 367
Apo TcPOP
MaxD
Relative Uptake (Da)
Exposure Time (minutes)
YCRLEDPTL
359 - 367
Apo TcPOP
MaxD
Relative Uptake (Da)
Exposure Time (minutes)
NAIPLPIG
368 - 375
Apo TcPOP
MaxD
Relative Uptake (Da)
Exposure Time (minutes)
NAIPLPIGTITSF
368 - 380
Apo TcPOP
MaxD
Relative Uptake (Da)
Exposure Time (minutes)
IPLPIGTITSF
370 - 380
Apo TcPOP
MaxD
Relative Uptake (Da)
Exposure Time (minutes)
PLPIGTITSF
371 - 380
Apo TcPOP
MaxD
Relative Uptake (Da)
Exposure Time (minutes)
PIGTITSF
373 - 380
Apo TcPOP
MaxD
Relative Uptake (Da)
Exposure Time (minutes)
FSDRKKDFVSF
381 - 391
Apo TcPOP
MaxD
Relative Uptake (Da)
Exposure Time (minutes)
KITSF
392 - 396
Apo TcPOP
MaxD
Relative Uptake (Da)
Exposure Time (minutes)

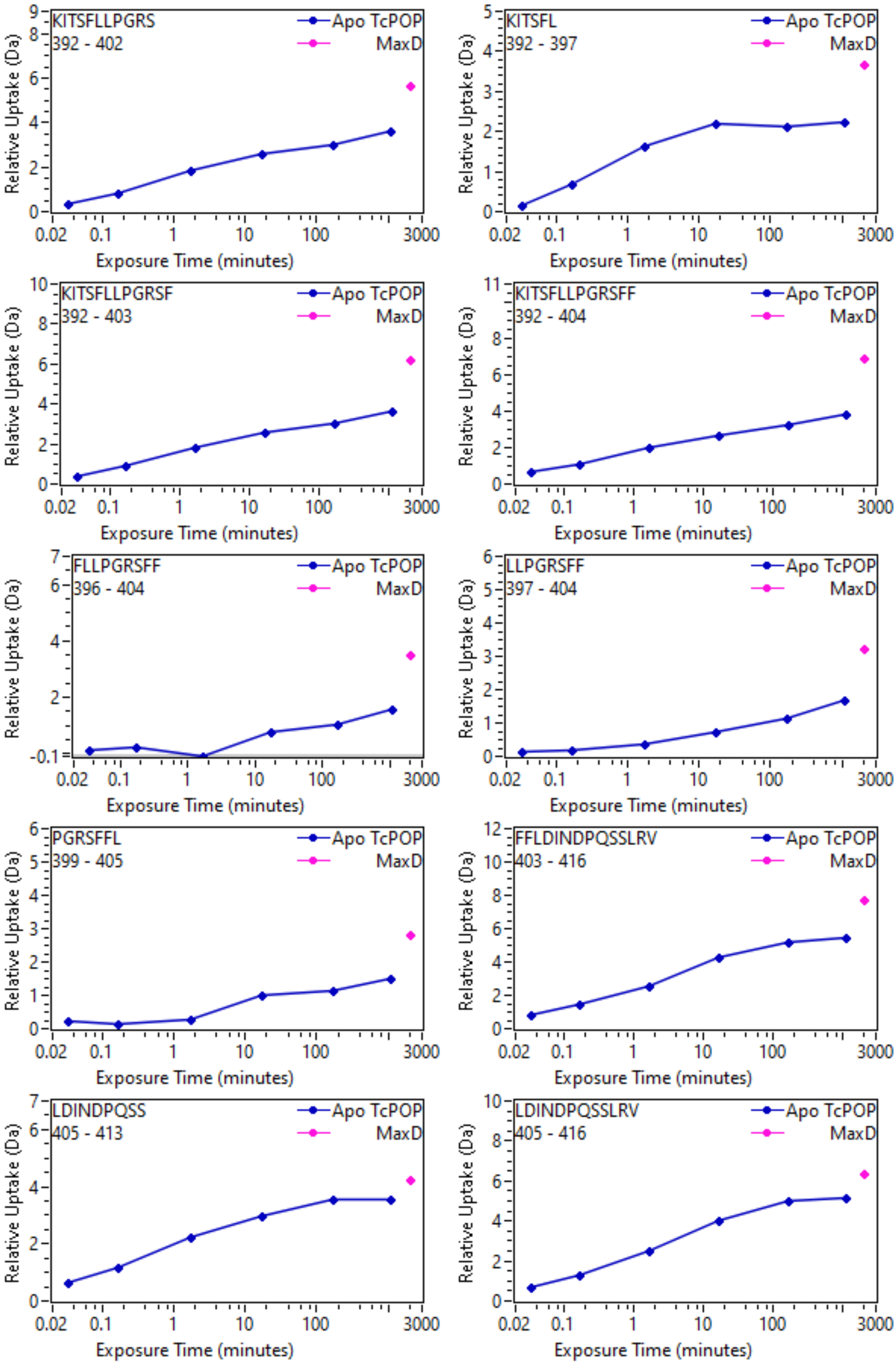
KITSFLLPGRS
392 - 402
KITSFL
392 - 397
KITSFLLPGRSF
392 - 403
KITSFLLPGRSFF
392 - 404
FLLPGRSFF
396 - 404
LLPGRSFF
397 - 404
PGRSFFL
399 - 405
FFLDINDPQSSLRV
403 - 416
LDINDPQSS
405 - 413
LDINDPQSSLRV
405 - 416
Apo TcPOP
MaxD
Relative Uptake (Da)
Exposure Time (minutes)
0.02
0.1
1
10
100
3000

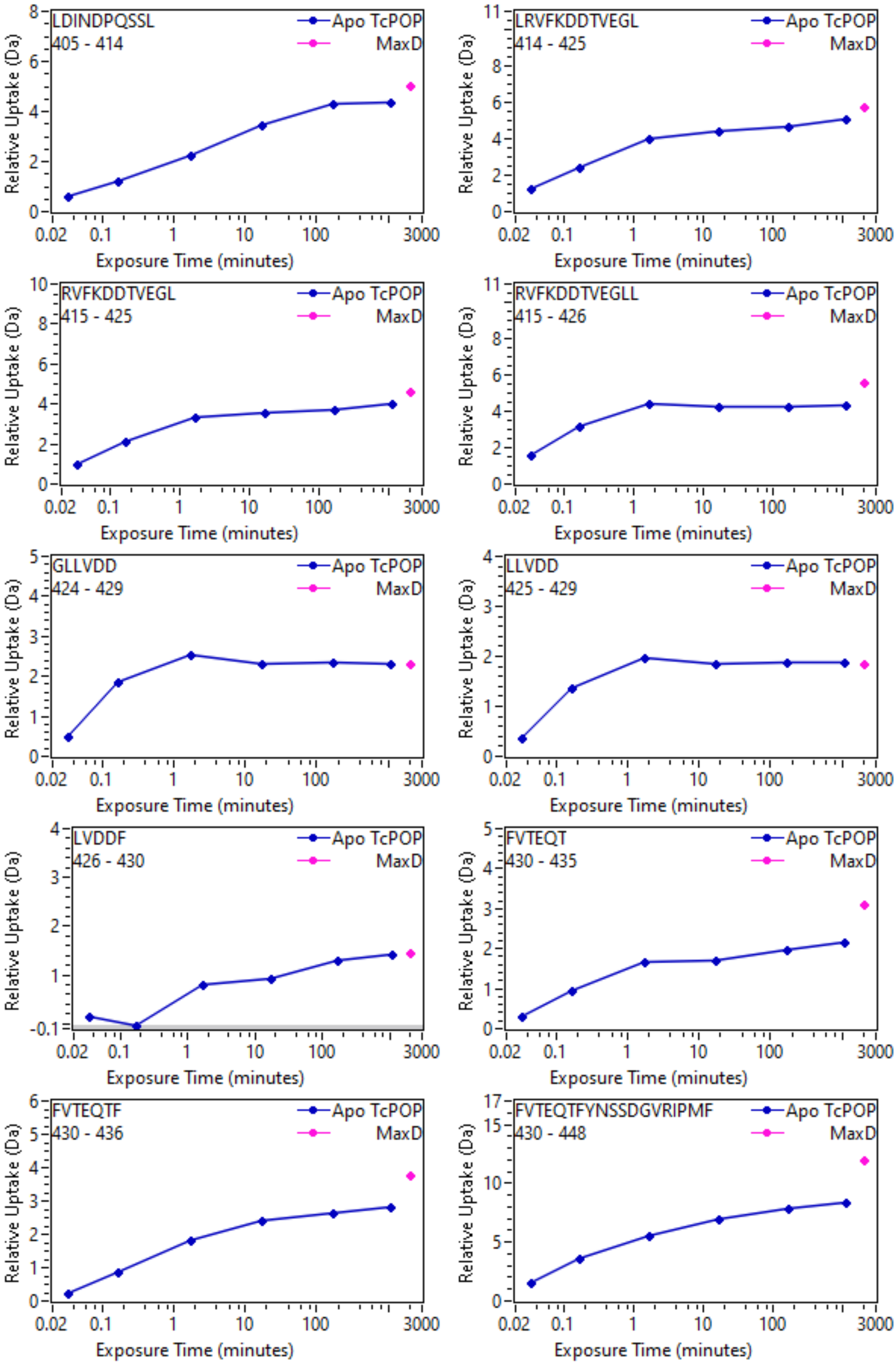
LDINDPQSSL
405 - 414
LRVFKDDTVEGL
414 - 425
RVFKDDTVEGL
415 - 425
RVFKDDTVEGLL
415 - 426
GLLVDD
424 - 429
LLVDD
425 - 429
LVDDF
426 - 430
FVTEQT
430 - 435
FVTEQTF
430 - 436
FVTEQTFYNSSDGVRIPMF
430 - 448
Apo TcPOP
MaxD
Relative Uptake (Da)
Exposure Time (minutes)
0.02
0.1
1
10
100
3000

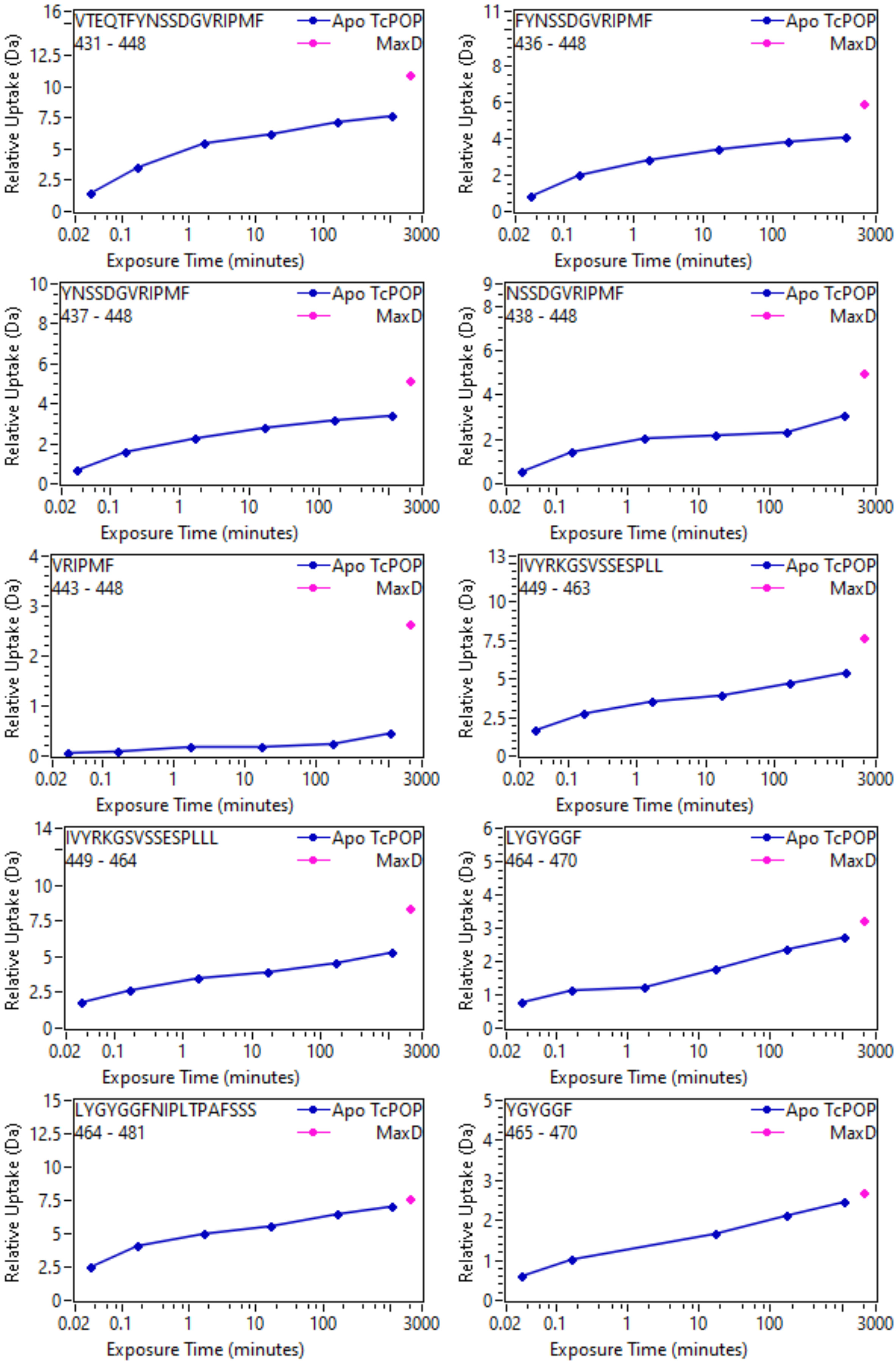
VTEQTFYNSSDGVRIPMF
431 - 448
FYNSSDGVRIPMF
436 - 448
YNSSDGVRIPMF
437 - 448
NSSDGVRIPMF
438 - 448
VRIPMF
443 - 448
IVYRKGSVSSESPLL
449 - 463
IVYRKGSVSSESPLLL
449 - 464
LYGYGGF
464 - 470
LYGYGGFNIPLTPAFSSS
464 - 481
YGYGGF
465 - 470
Apo TcPOP
MaxD
Relative Uptake (Da)
Exposure Time (minutes)
0.02
0.1
1
10
100
3000

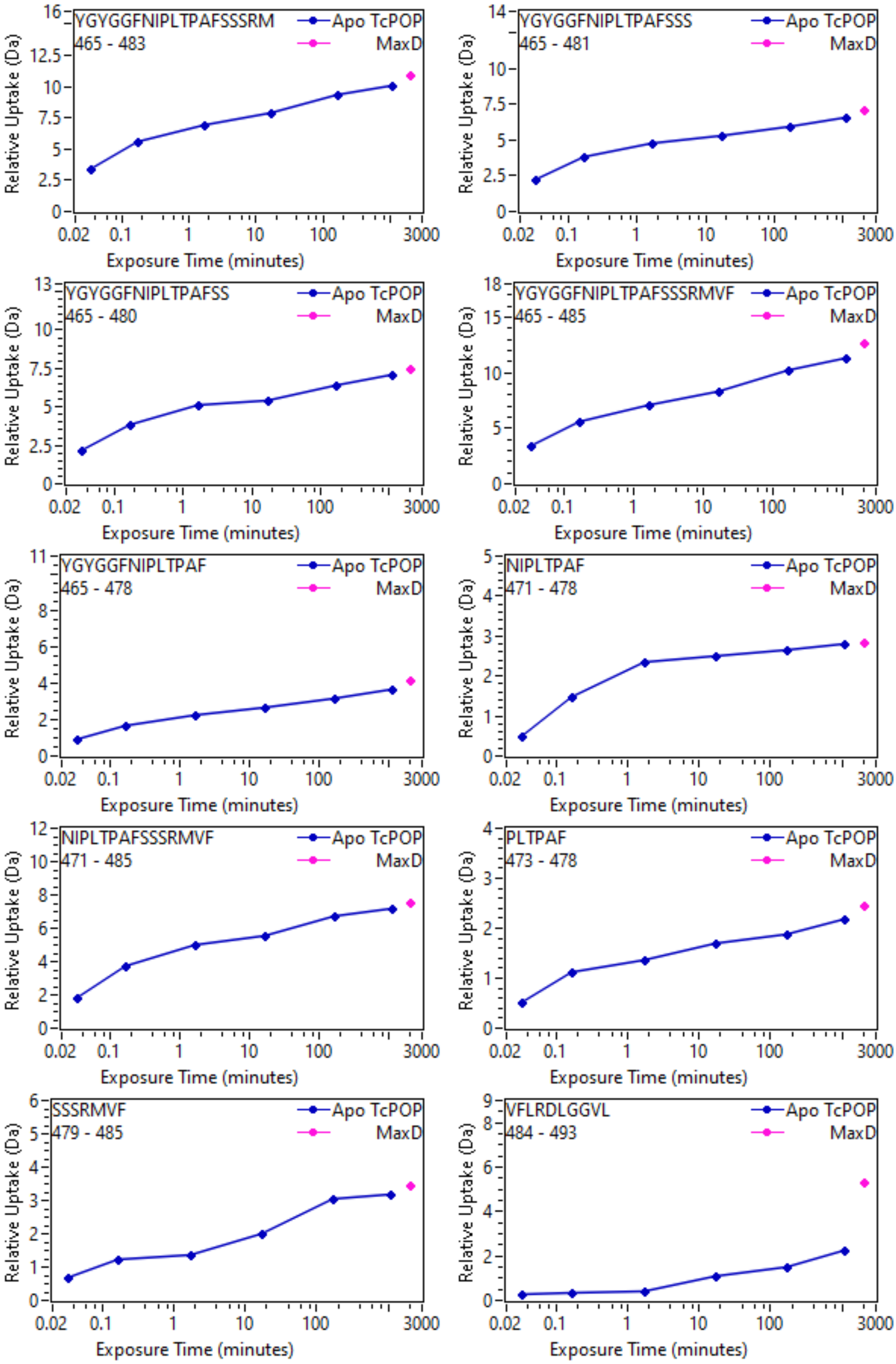
YGYGGFNIPLTPAFSSSRM
465 - 483
YGYGGFNIPLTPAFSSS
465 - 481
YGYGGFNIPLTPAFSS
465 - 480
YGYGGFNIPLTPAFSSSRMVF
465 - 485
YGYGGFNIPLTPAF
465 - 478
NIPLTPAF
471 - 478
NIPLTPAFSSSRMVF
471 - 485
PLTPAF
473 - 478
SSSRMVF
479 - 485
VFLRDLGGVL
484 - 493
Apo TcPOP
MaxD
Relative Uptake (Da)
Exposure Time (minutes)
0.02
0.1
1
10
100
3000

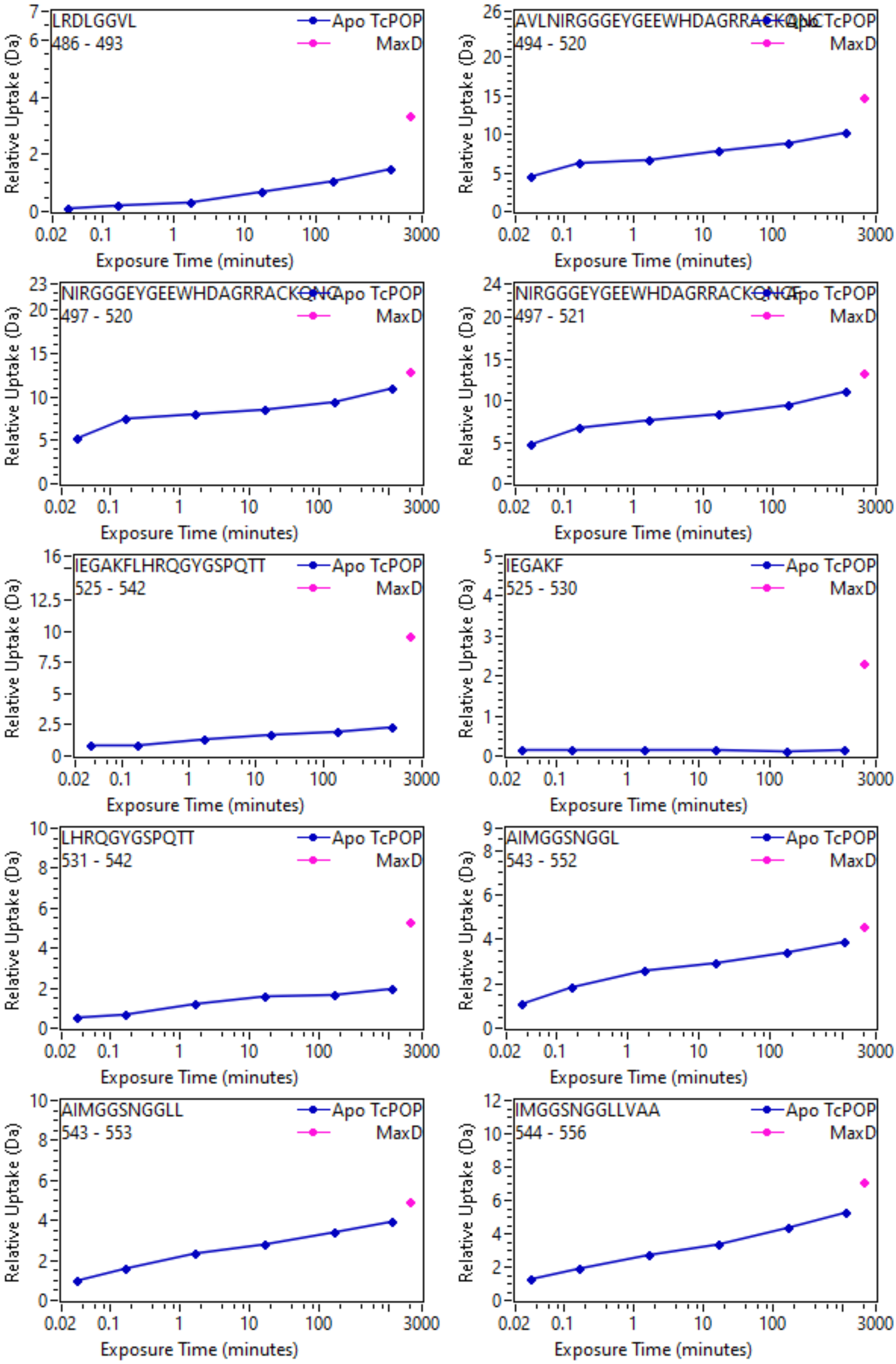

LRDLGGVL
486 - 493
AVLNIRGGGEYGEEWHDAGRRACK
494 - 520
NIRGGGEYGEEWHDAGRRACK
497 - 520
NIRGGGEYGEEWHDAGRRACK
497 - 521
IEGAKFLHRQGYGSPQTT
525 - 542
IEGAKF
525 - 530
LHRQGYGSPQTT
531 - 542
AIMGGSNGGL
543 - 552
AIMGGSNGGLL
543 - 553
IMGGSNGGLLVAA
544 - 556
Apo TcPOP
MaxD
Relative Uptake (Da)
Exposure Time (minutes)
0.02
0.1
1
10
100
3000

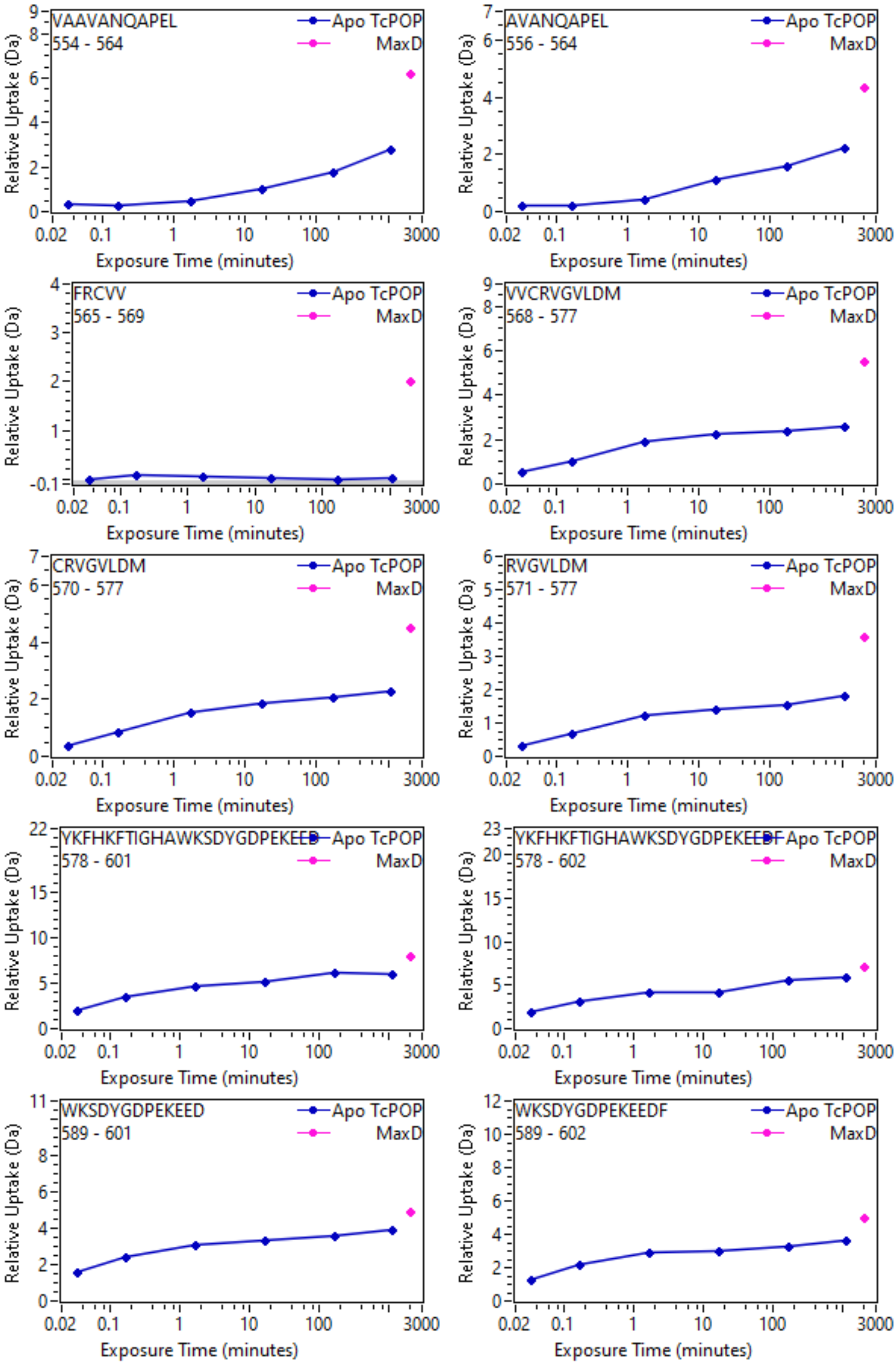

VAAVANQAPEL
554 - 564
AVANQAPEL
556 - 564
FRCVV
565 - 569
VVCRVGVLDM
568 - 577
CRVGVLDM
570 - 577
RVGVLDM
571 - 577
YKFHKFTIGHAWKSDYGDPEKEED
578 - 601
YKFHKFTIGHAWKSDYGDPEKEEDF
578 - 602
WKSDYGDPEKEED
589 - 601
WKSDYGDPEKEEDF
589 - 602
Apo TcPOP
MaxD
Relative Uptake (Da)
Exposure Time (minutes)

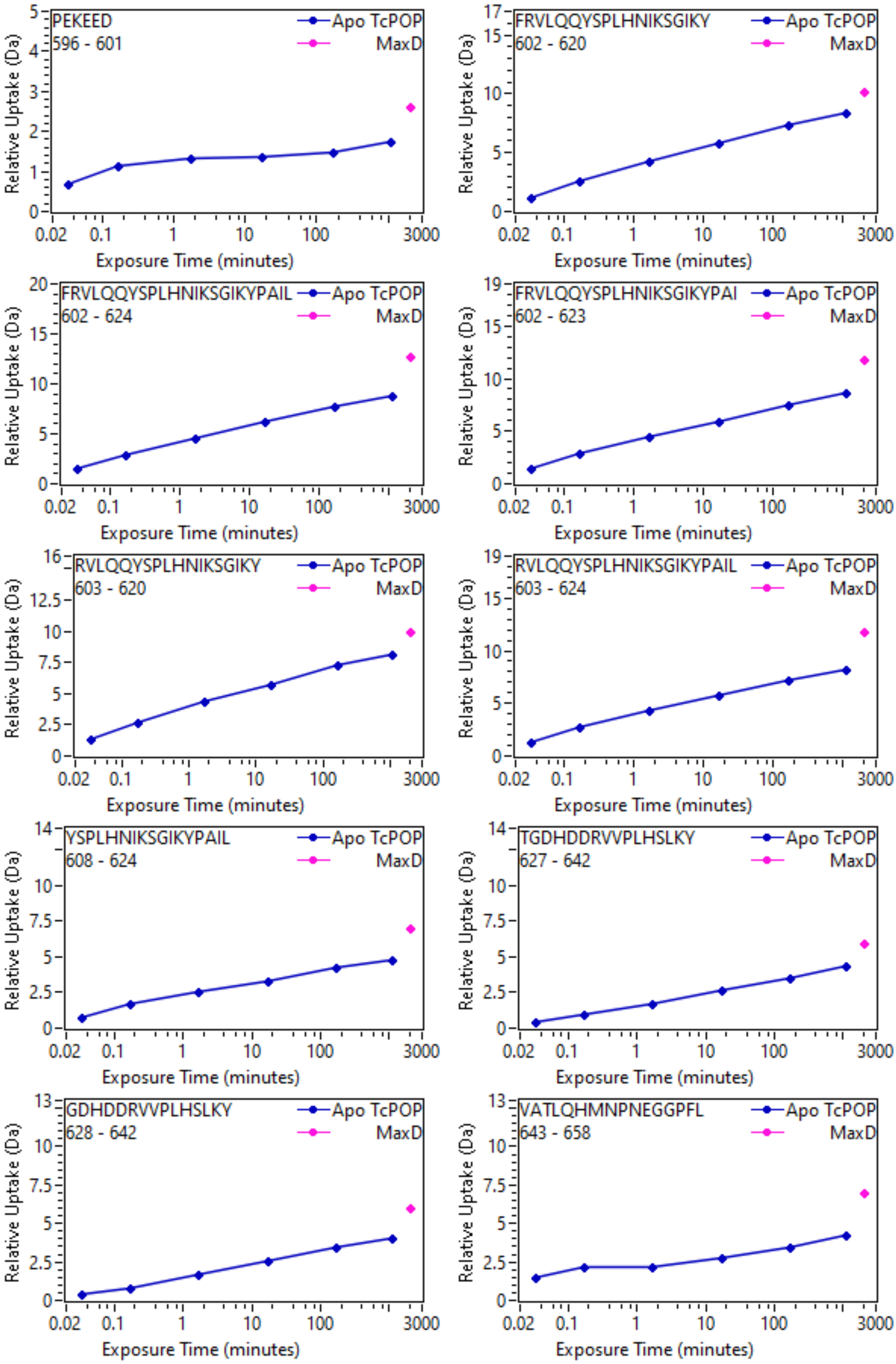
PEKEED
596 - 601
FRVLQQYSPLHNIKSGIKY
602 - 620
FRVLQQYSPLHNIKSGIKYPAIL
602 - 624
FRVLQQYSPLHNIKSGIKYPAI
602 - 623
RVLQQYSPLHNIKSGIKY
603 - 620
RVLQQYSPLHNIKSGIKYPAIL
603 - 624
YSPLHNIKSGIKYPAIL
608 - 624
TGDHDDRVVPLHSLKY
627 - 642
GDHDDRVVPLHSLKY
628 - 642
VATLQHMNPNEGGPFL
643 - 658
Apo TcPOP
MaxD
Relative Uptake (Da)
Exposure Time (minutes)
0.02
0.1
1
10
100
3000

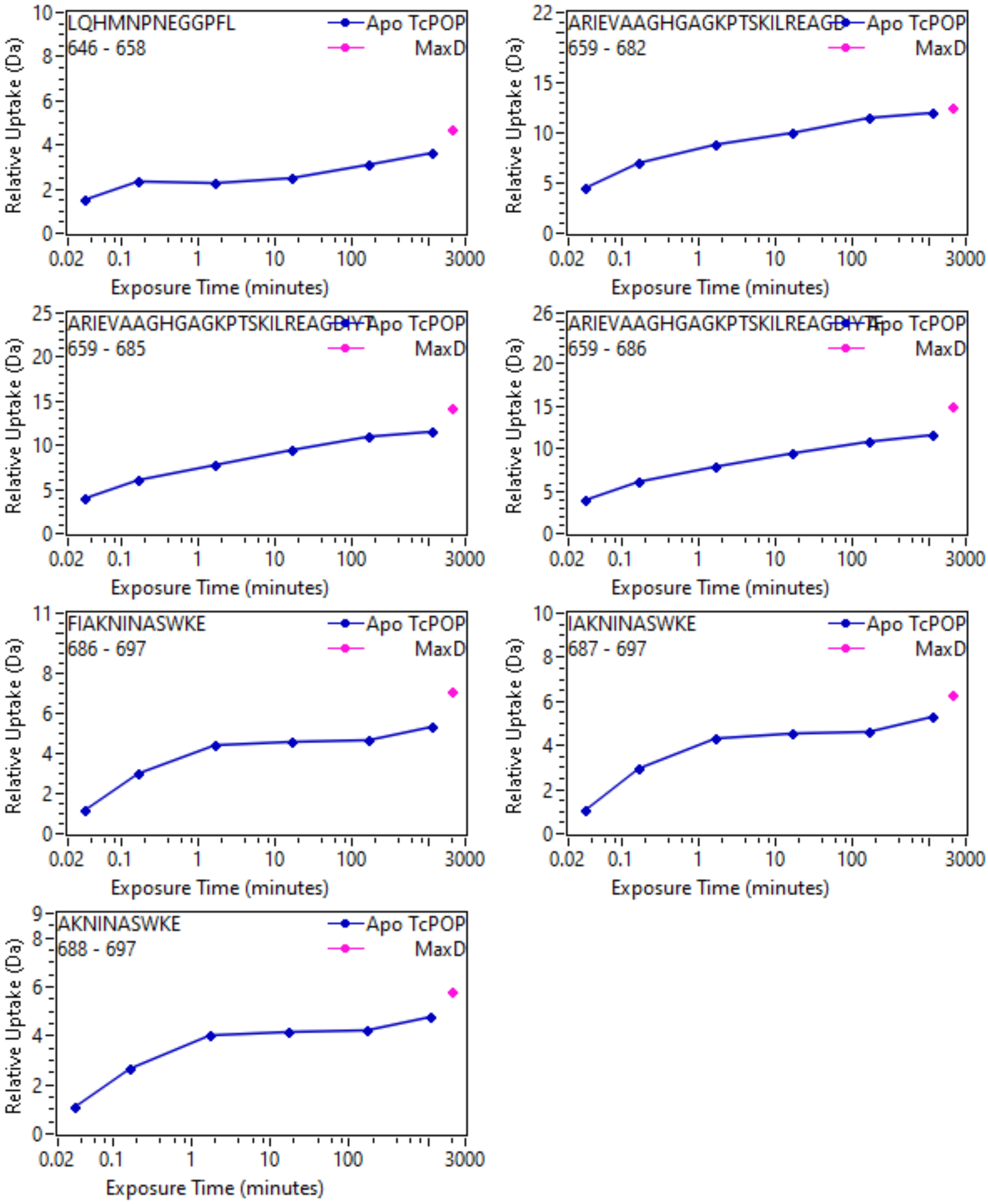

Supplementary Fig. S19 | Deuterium uptake plots of the 197 peptides whose HDX was followed. The isotopic envelope centroid mass was considered for calculating the deuterium uptake, also in the case of peptides manifesting EX1 kinetics. Source data are provided as Source Data File.

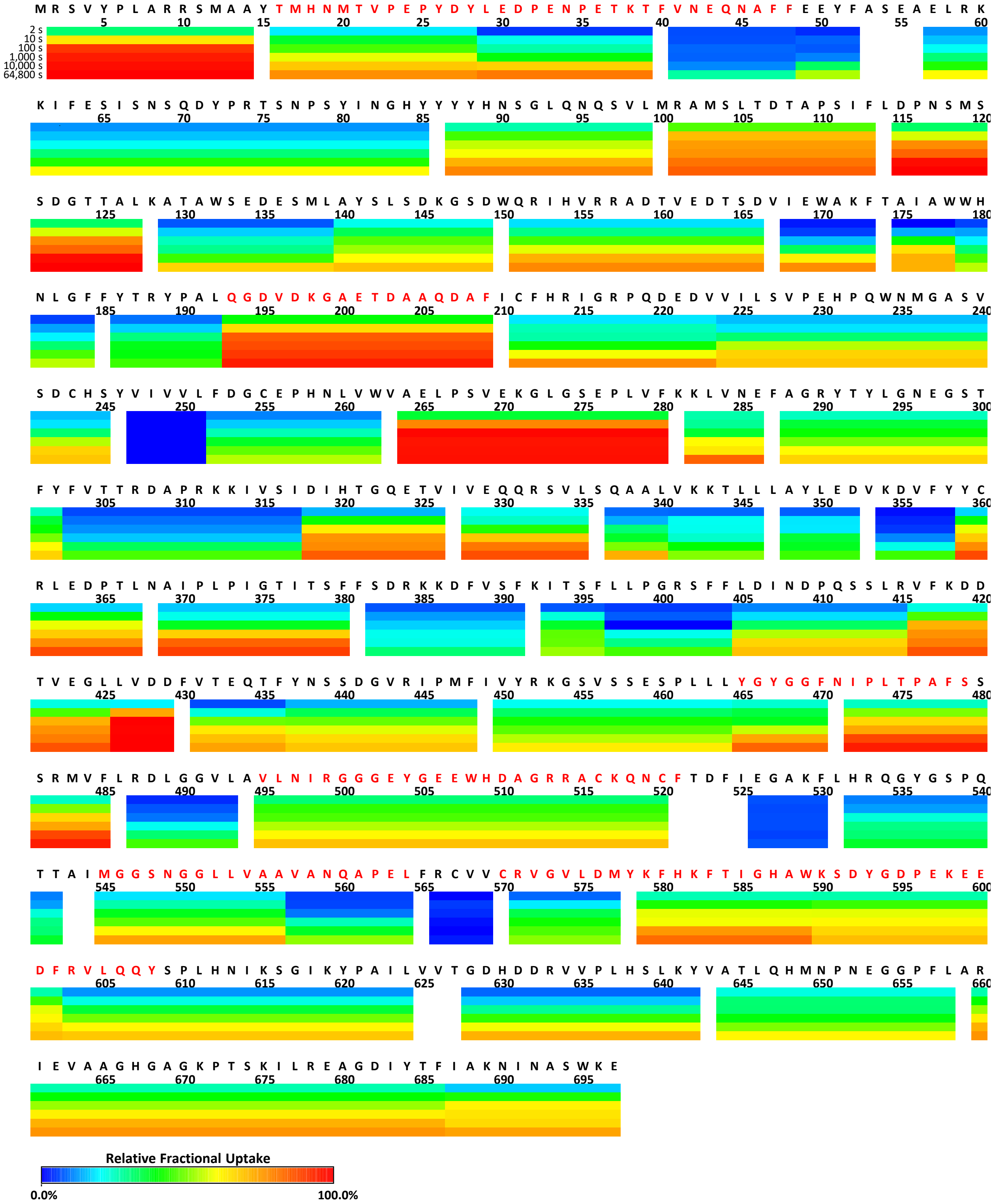

Supplementary Fig. S20 | Heat map of TcPOP local flexibility and structural dynamics. The relative fractional deuterium uptake at the time points studied (from 2 s to 18 h) for selected TcPOP peptides (normalized by MaxD control) is plotted as rainbow colour scale along the protein sequence of TcPOP. The isotopic envelope centroid mass was considered for calculating the deuterium uptake, also in the

case of peptides manifesting EX1 kinetics. Protein regions exhibiting EX1/EXx kinetics are coloured in red. Peptides selected for the analysis are: 1-14, 15-28, 28-39, 40-48, 48-52, 56-85, 86-99, 100-113, 114-127, 128-139, 139-149, 150-166, 167-173, 174-178, 178-184, 185-192, 190-209, 210-223, 223-245, 246-251, 251-262, 263-280, 281-286, 287-302, 302-318, 317-326, 327-335, 336-340, 340-346, 347-352, 353-358, 358-367, 368-380, 381-391, 392-396, 396-404, 403-416, 415-425, 425-429, 430-436, 436-448, 449-464, 464-470, 471-485, 486-493, 494-520, 525-530, 531-542, 544-556, 556-564, 565-569, 570-577, 578-602, 589-602, 602-624, 627-642, 643-658, 659-686, and 686-697. Source data are provided as Source Data File.

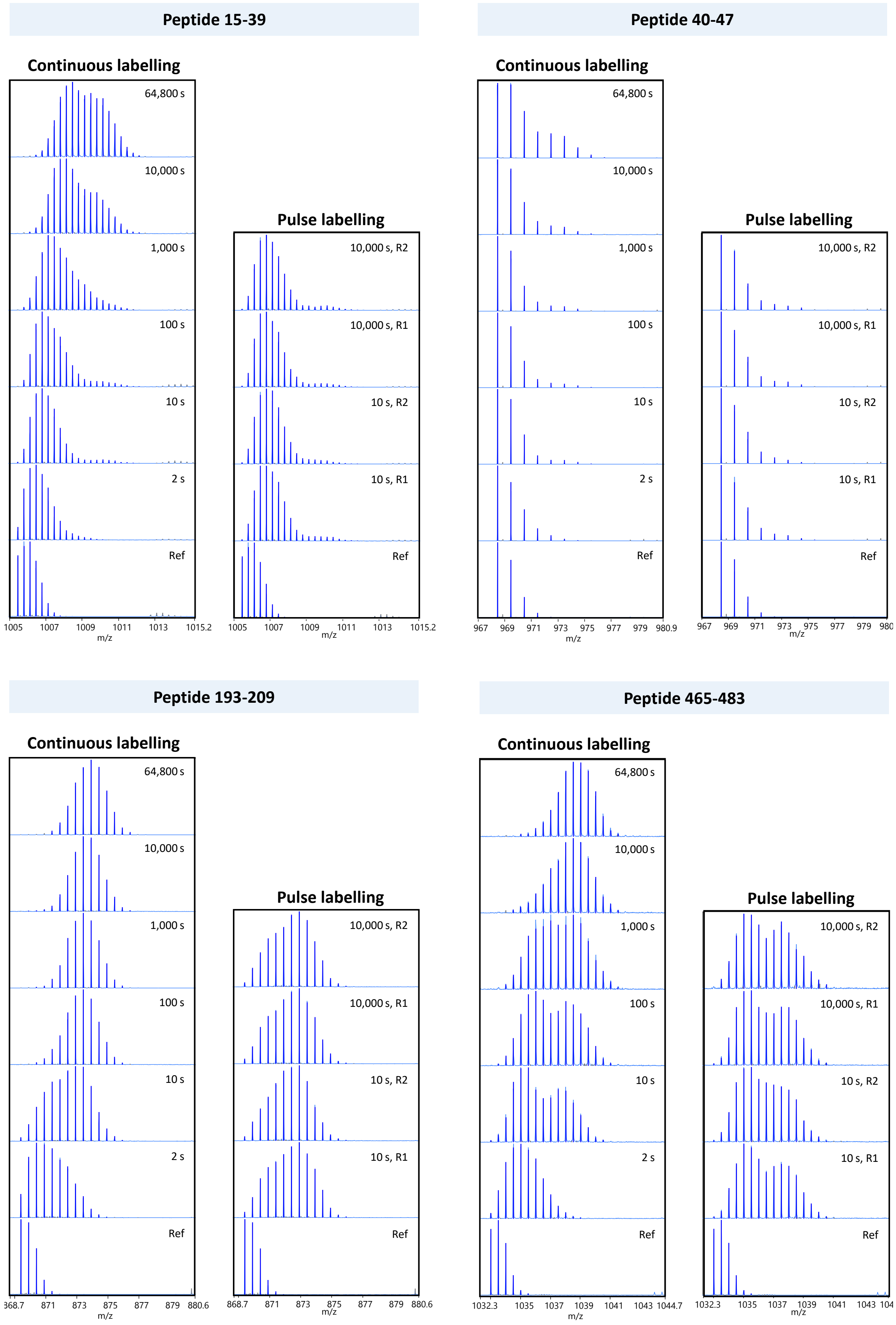
Peptide 15-39
Continuous labelling
64,800 s
10,000 s
1,000 s
100 s
10 s
2 s
Ref
1005 1007 1009 1011 1013 1015.2
m/z
Pulse labelling
10,000 s, R2
10,000 s, R1
10 s, R2
10 s, R1
Ref
1005 1007 1009 1011 1013 1015.2
m/z
Peptide 40-47
Continuous labelling
64,800 s
10,000 s
1,000 s
100 s
10 s
2 s
Ref
967 969 971 973 975 977 979 980.9
m/z
Pulse labelling
10,000 s, R2
10,000 s, R1
10 s, R2
10 s, R1
Ref
967 969 971 973 975 977 979 980
m/z
Peptide 193-209
Continuous labelling
64,800 s
10,000 s
1,000 s
100 s
10 s
2 s
Ref
368.7 871 873 875 877 879 880.6
m/z
Pulse labelling
10,000 s, R2
10,000 s, R1
10 s, R2
10 s, R1
Ref
868.7 871 873 875 877 879 880.6
m/z
Peptide 465-483
Continuous labelling
64,800 s
10,000 s
1,000 s
100 s
10 s
2 s
Ref
1032.3 1035 1037 1039 1041 1043 1044.7
m/z
Pulse labelling
10,000 s, R2
10,000 s, R1
10 s, R2
10 s, R1
Ref
1032.3 1035 1037 1039 1041 1043 104
m/z

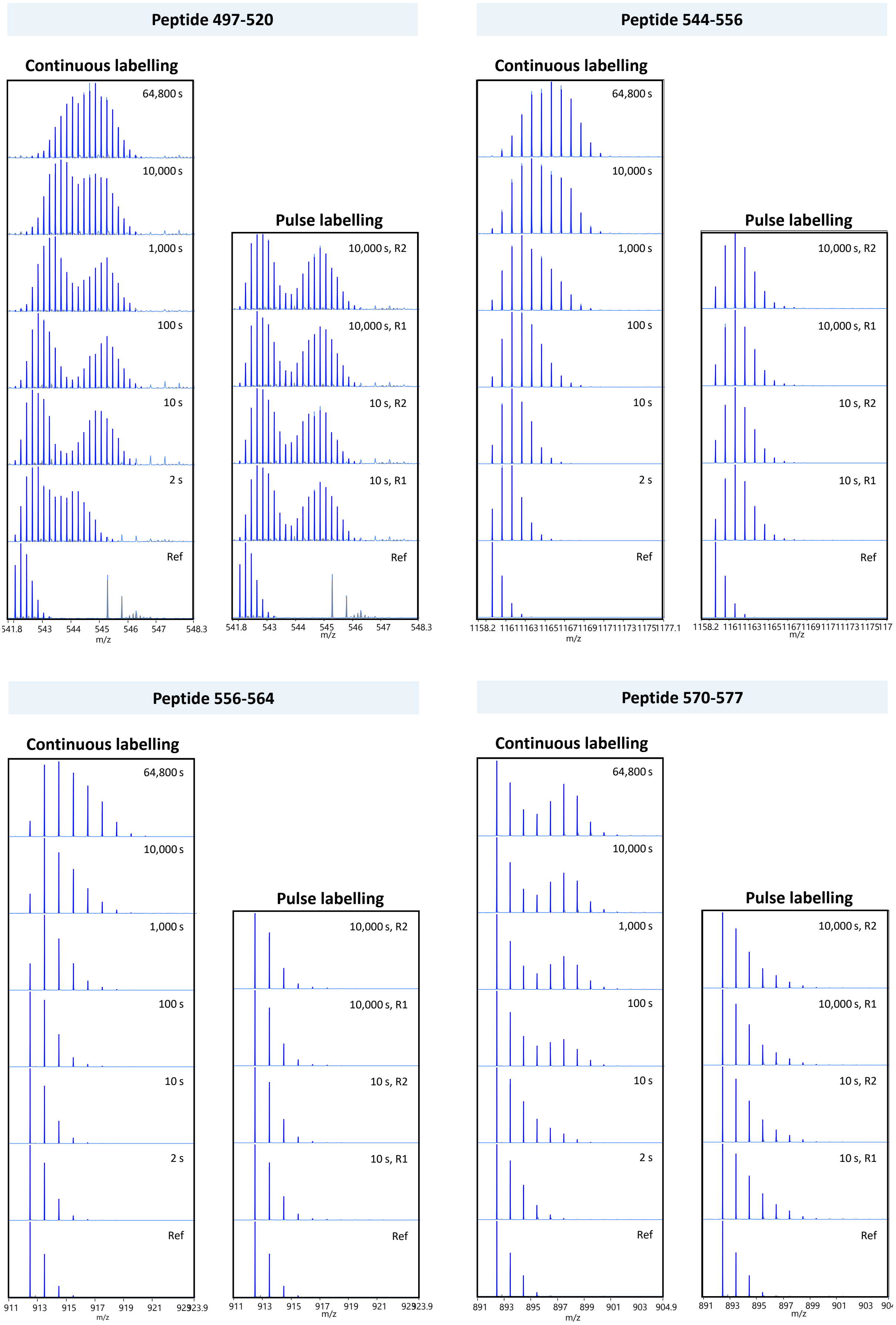
Peptide 497-520
Continuous labelling
64,800 s
10,000 s
1,000 s
100 s
10 s
2 s
Ref
541.8 543 544 545 546 547 548.3
m/z
Pulse labelling
10,000 s, R2
10,000 s, R1
10 s, R2
10 s, R1
Ref
541.8 543 544 545 546 547 548.3
m/z
Peptide 544-556
Continuous labelling
64,800 s
10,000 s
1,000 s
100 s
10 s
2 s
Ref
1158.2 1177.1
m/z
Pulse labelling
10,000 s, R2
10,000 s, R1
10 s, R2
10 s, R1
Ref
1158.2
m/z
Peptide 556-564
Continuous labelling
64,800 s
10,000 s
1,000 s
100 s
10 s
2 s
Ref
911 913 915 917 919 921 923.9
m/z
Pulse labelling
10,000 s, R2
10,000 s, R1
10 s, R2
10 s, R1
Ref
911 913 915 917 919 921 923.9
m/z
Peptide 570-577
Continuous labelling
64,800 s
10,000 s
1,000 s
100 s
10 s
2 s
Ref
891 893 895 897 899 901 903 904.9
m/z
Pulse labelling
10,000 s, R2
10,000 s, R1
10 s, R2
10 s, R1
Ref
891 893 895 897 899 901 903
m/z

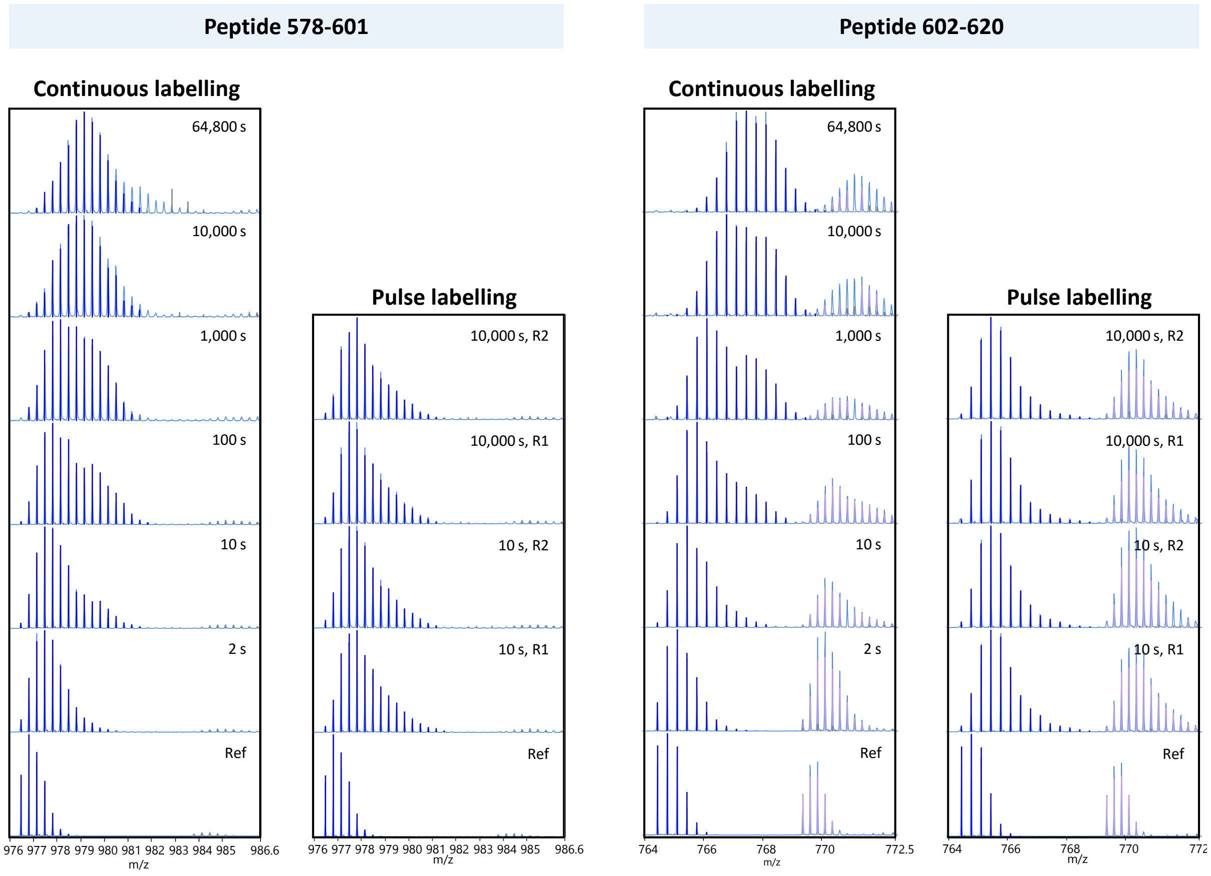

Supplementary Fig. S21 | Raw spectra (exported from DynamX software) of selected peptides exhibiting EX1 or EXx kinetics at the different time points studied (continuous labelling) and at different times of exposure to room temperature after sample unthawing followed by 10 s-pulse labelling. The continuous labelling experiment shows the interconversion of the low- and high-mass envelopes over time. The pulse labelling experiment show that the high-mass envelope is consistent in mass and size over time, therefore the protein is stable under the HDX condition studied (no irreversible unfolding), evidencing that the opening and closing events are in a dynamic equilibrium.

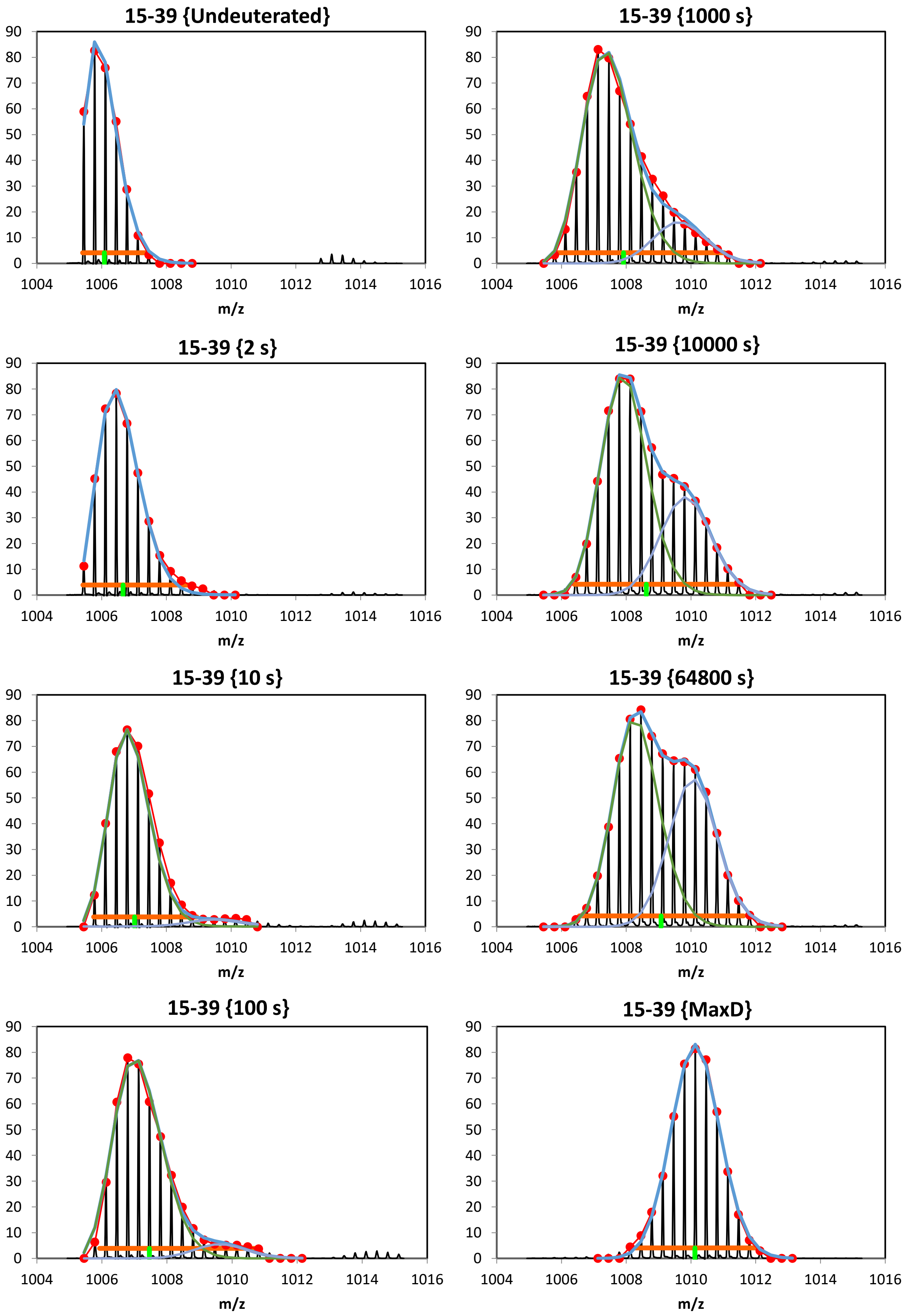

Supplementary Fig. S22a.

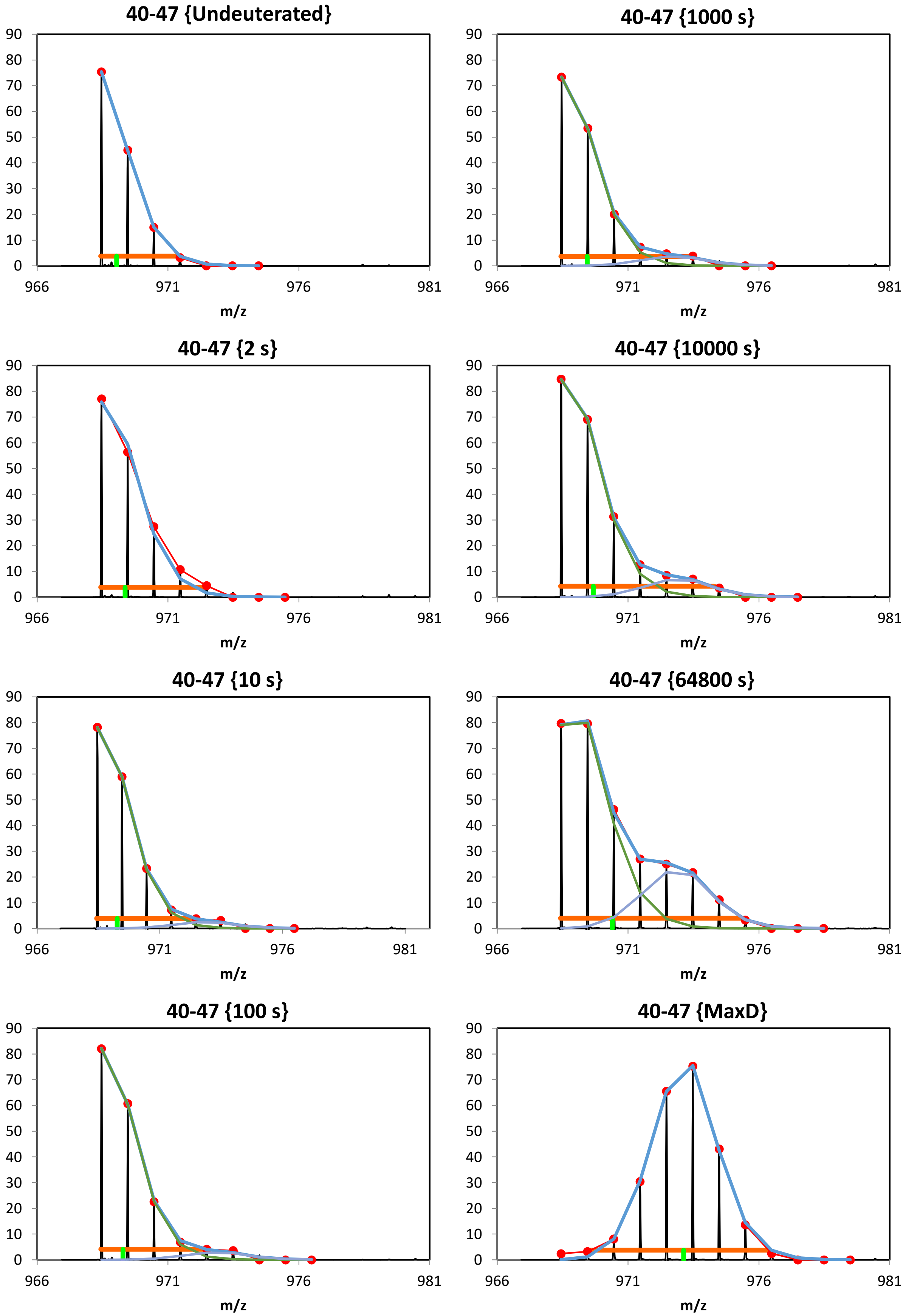

Supplementary Fig. S22b.

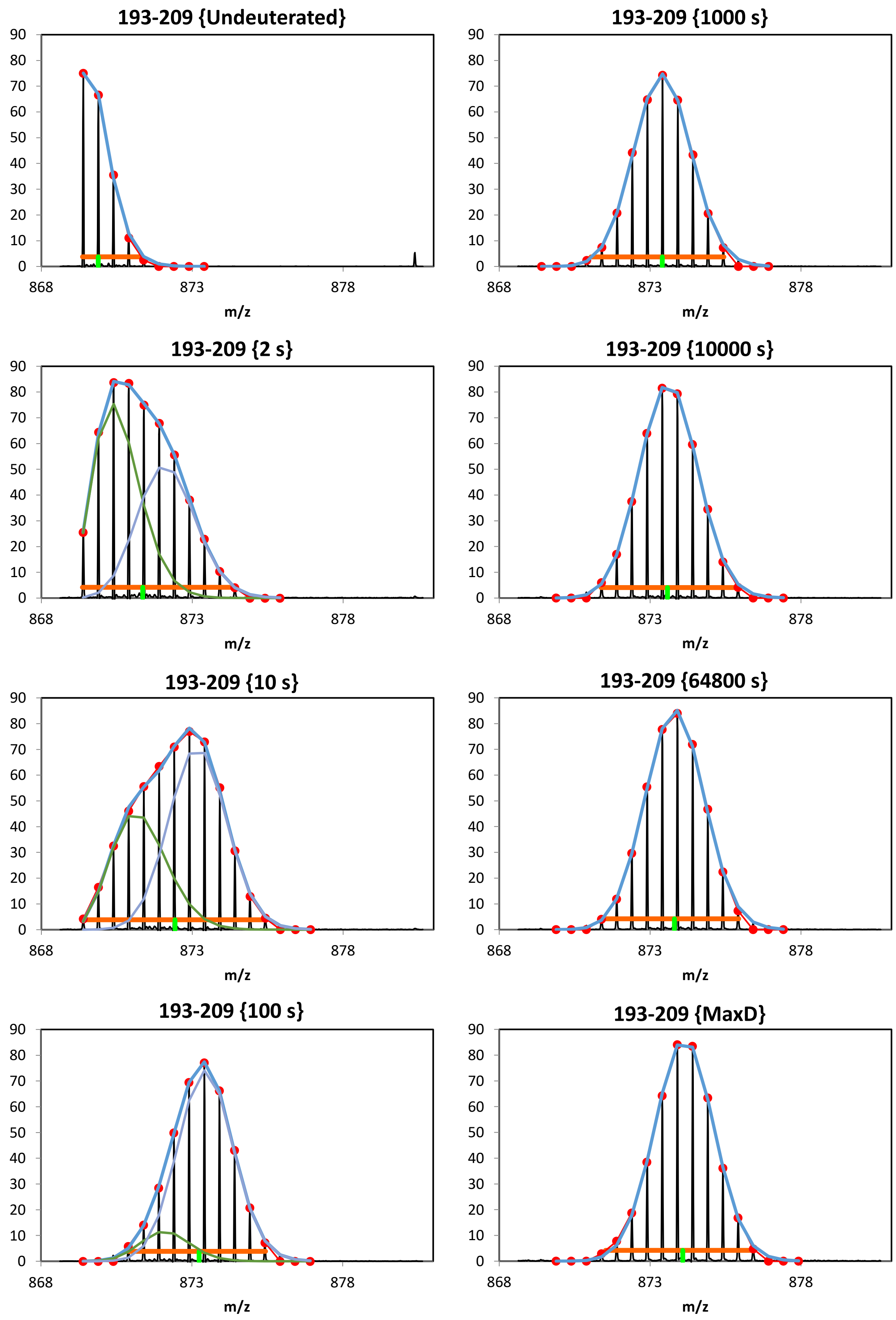

Supplementary Fig. S22c.

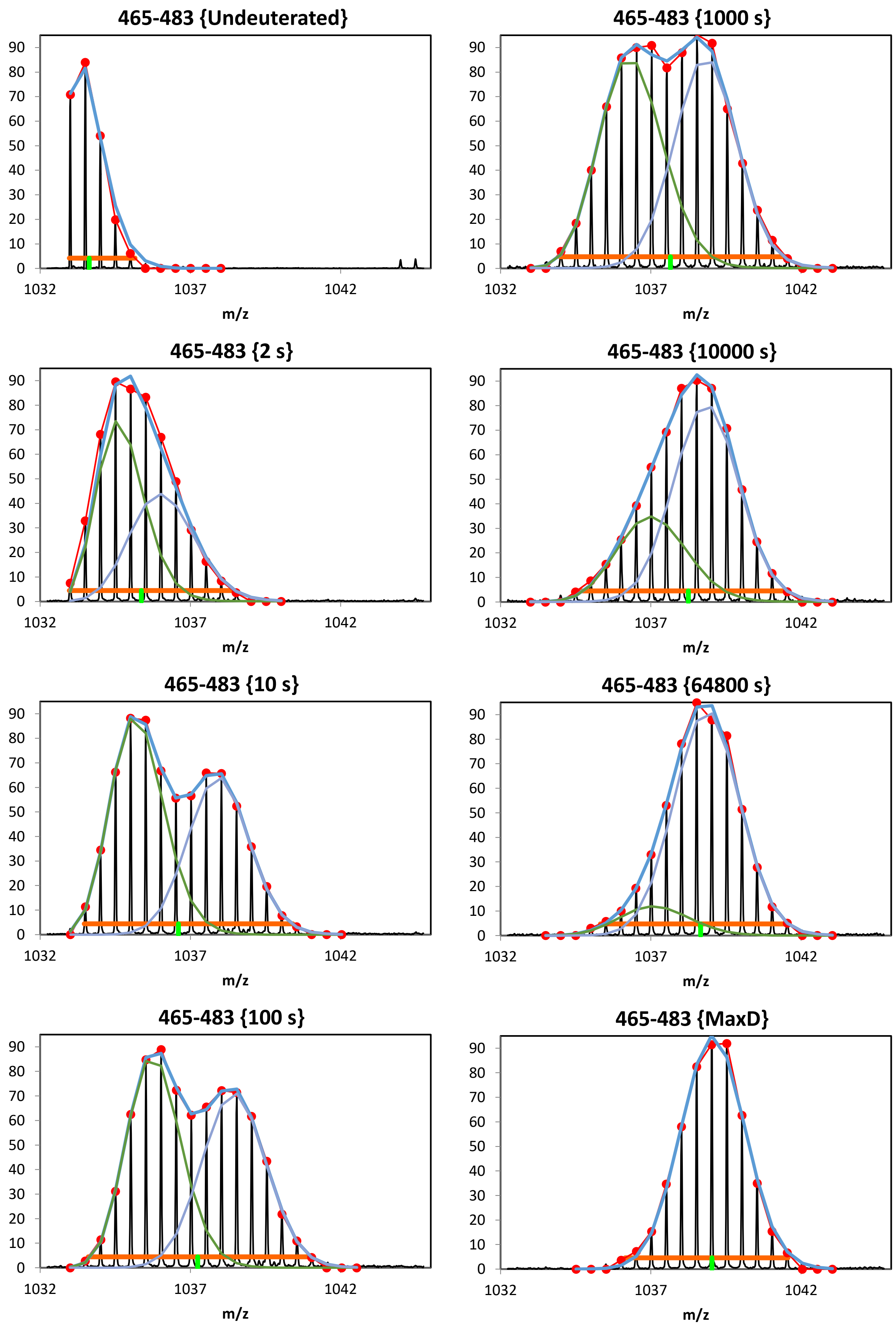

Supplementary Fig. S22d.

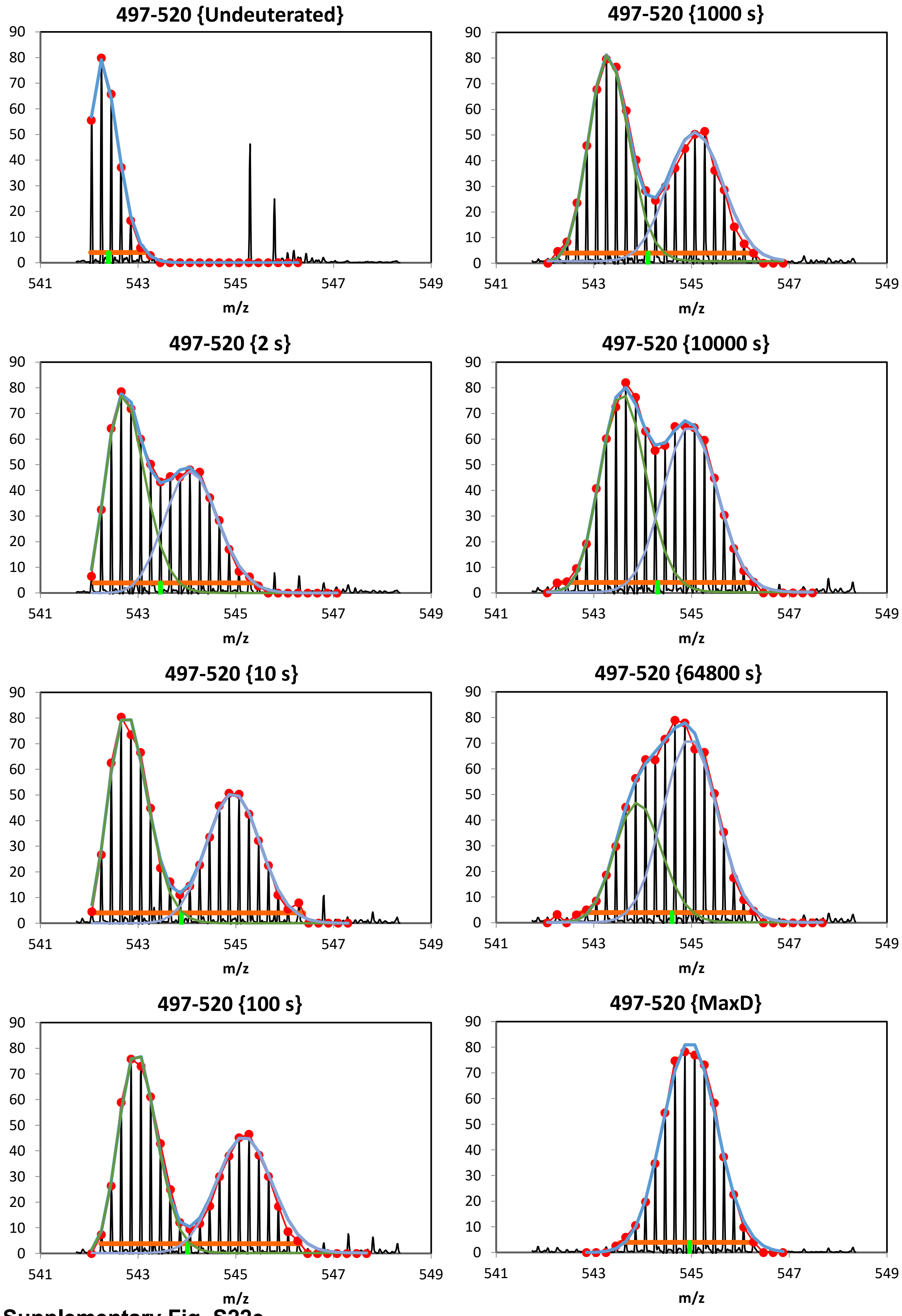

Supplementary Fig. S22e.

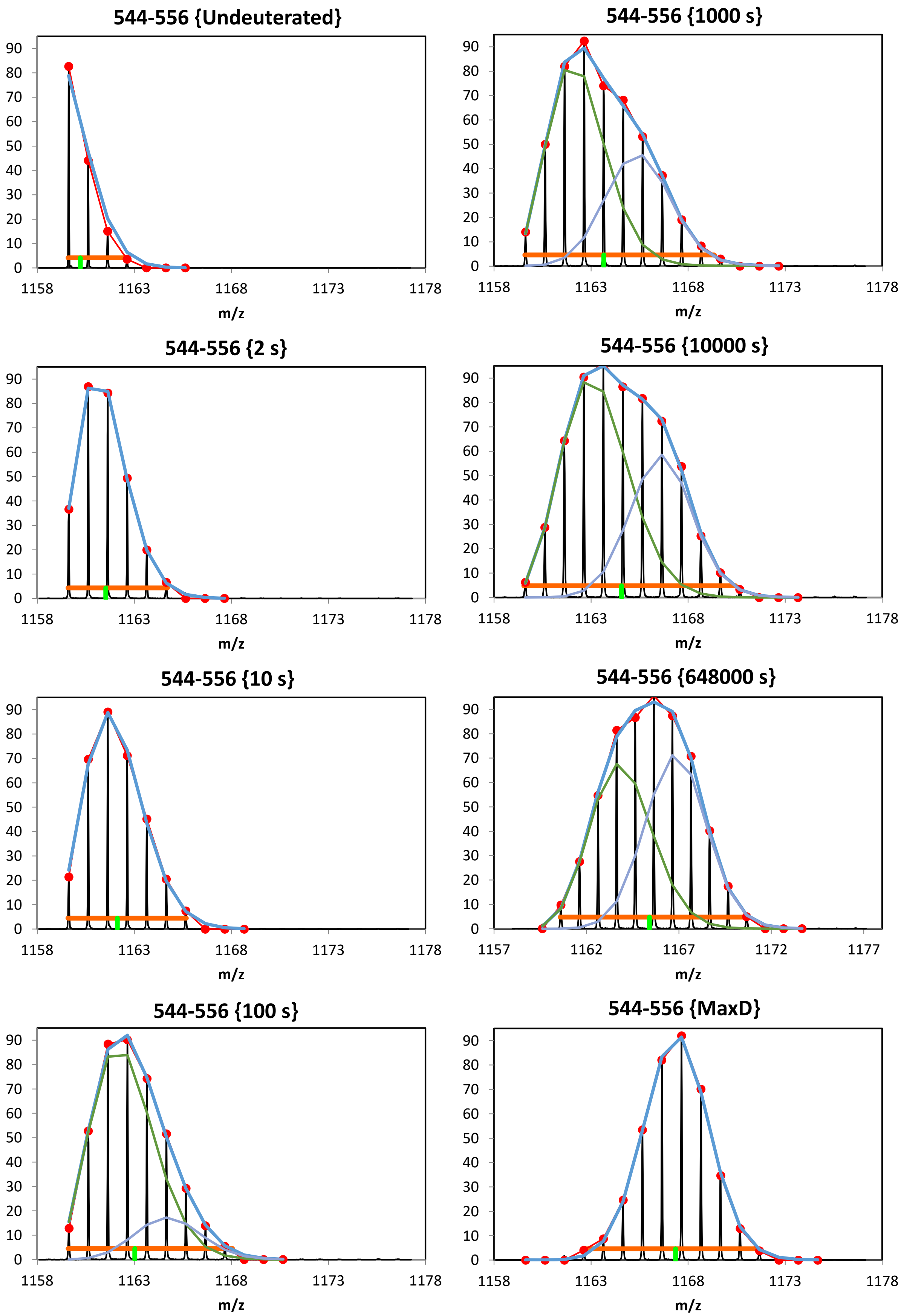

Supplementary Fig. S22f.

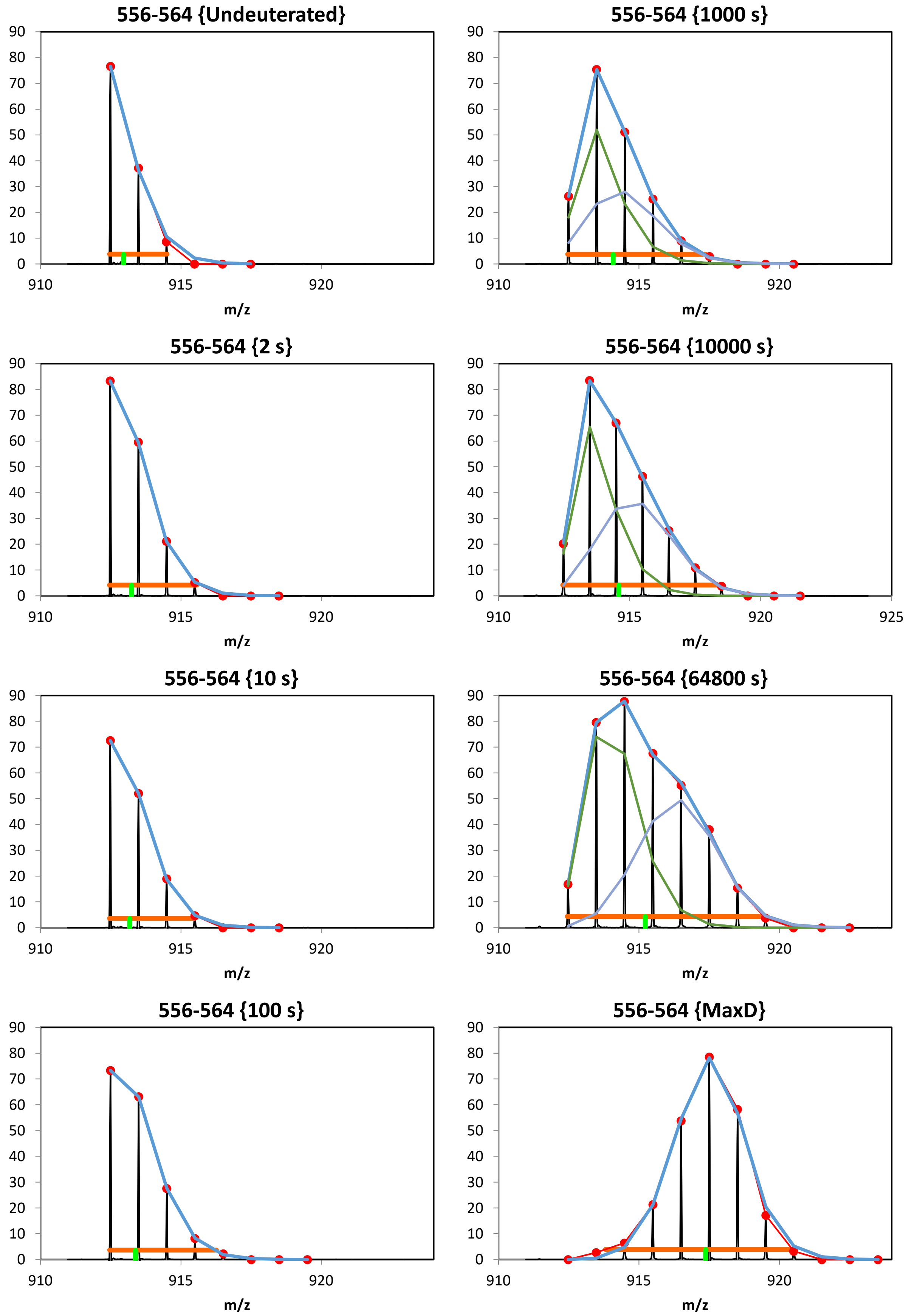

Supplementary Fig. S22g.

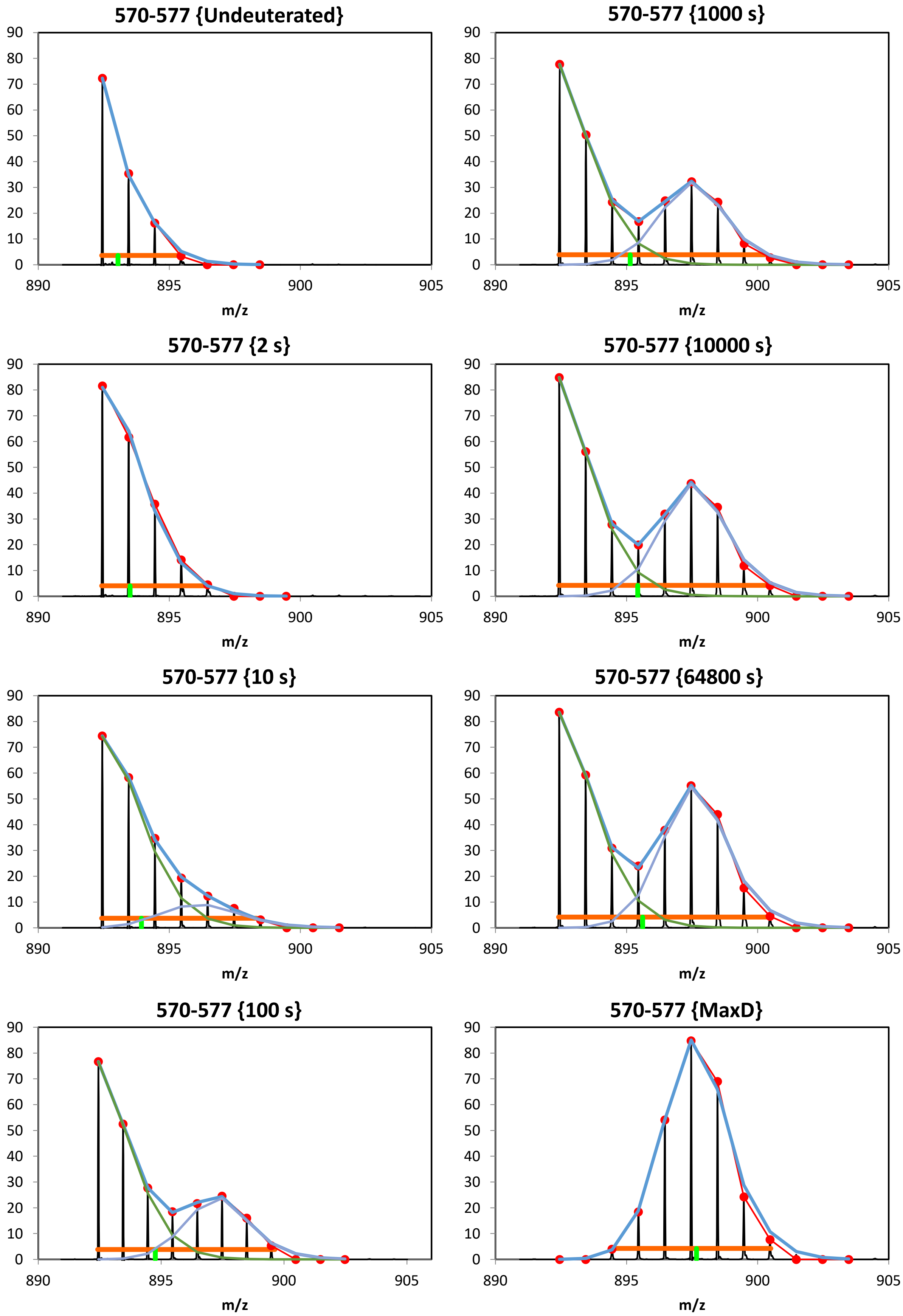

Supplementary Fig. S22h.

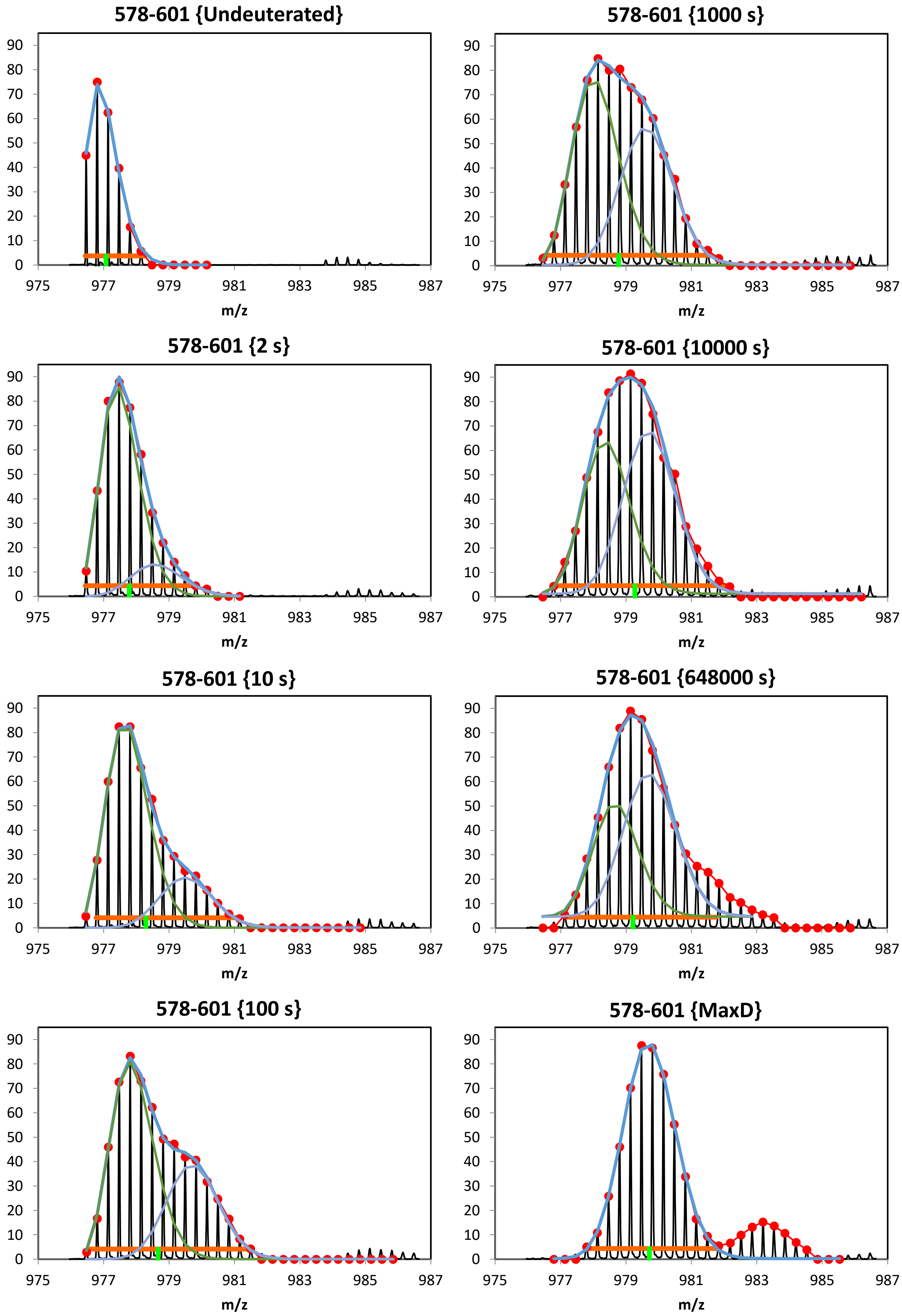

Supplementary Fig. S22i.

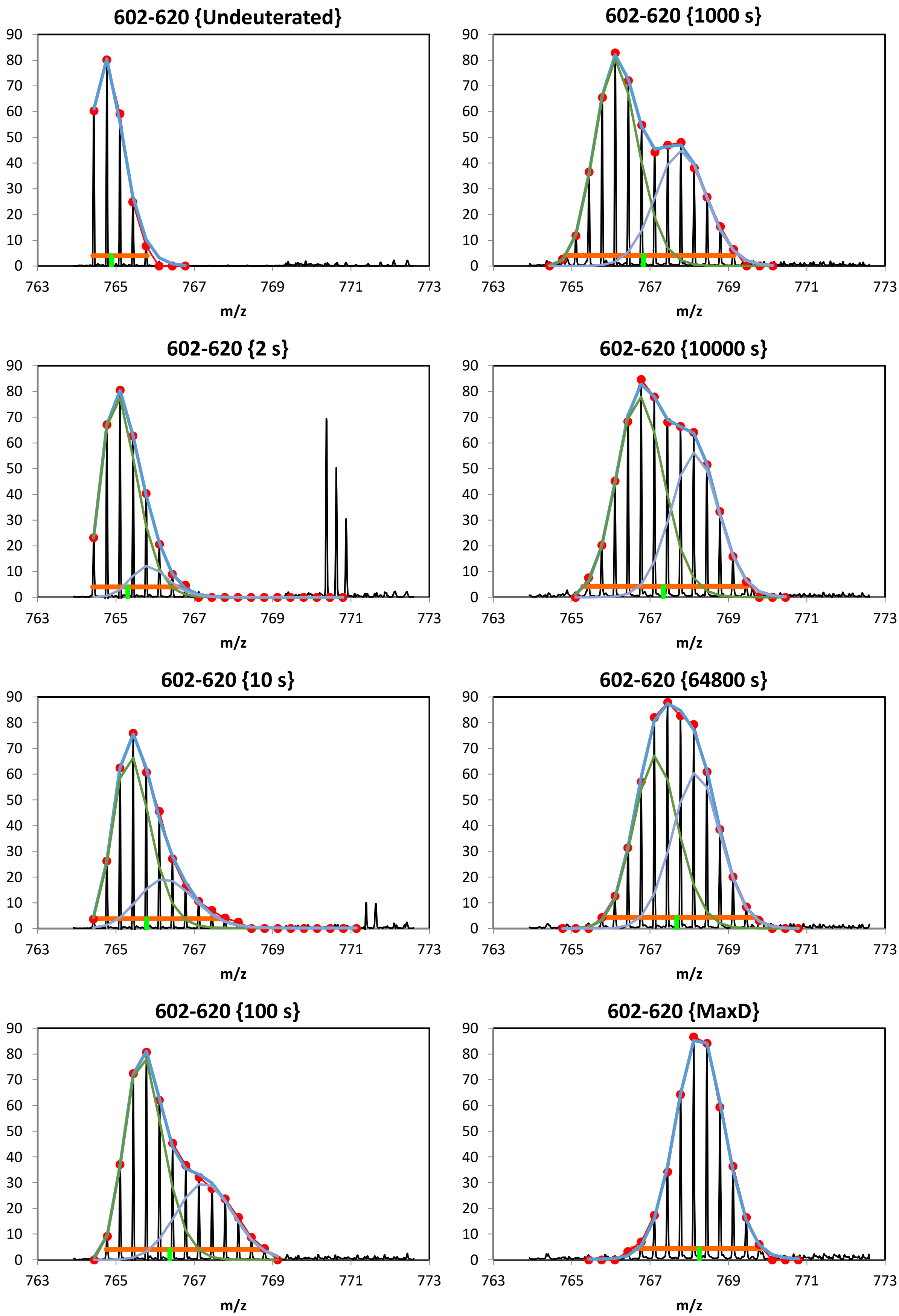

Supplementary Fig. S22j.

Supplementary Figs. S22a-j | Binomial fittings of the bimodal isotopic envelopes observed for peptides 15-39 (a), 40-47 (b), 193-209 (c), 465-483 (d), 497-520 (e), 544-556 (f), 556-564 (g), 570-577 (h), 578-601 (i), 602-620 (j) performed with HX-Express software. Blue line: fitting of the whole isotopic envelope; green line: fitting of the low-mass envelope; light blue line: fitting of the high-mass envelope; orange line: full-width at 5% BPI: bright green dash: centroid mass of the whole isotopic envelope.

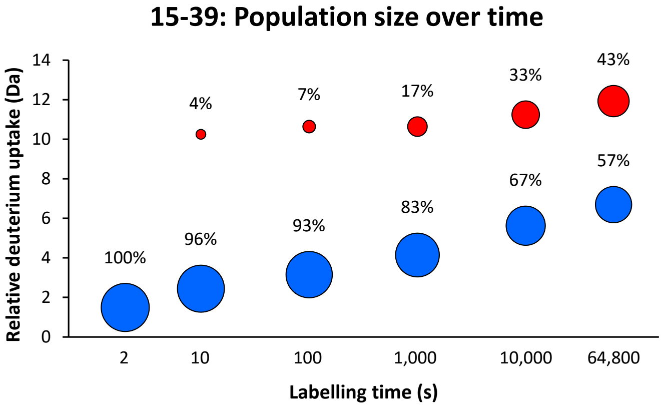
15-39: Population size over time
Relative deuterium uptake (Da)
0 2 4 6 8 10 12 14
4% 7% 17% 33% 43%
100% 96% 93% 83% 67% 57%
2 10 100 1,000 10,000 64,800
Labelling time (s)

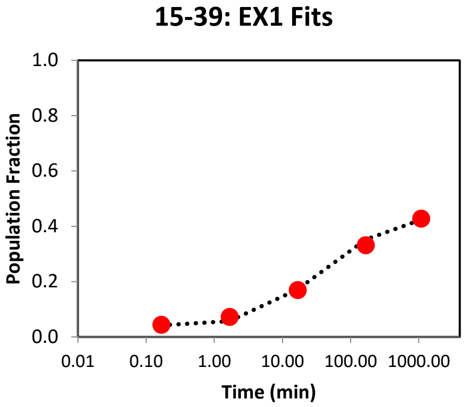
15-39: EX1 Fits
Population Fraction
0.0 0.2 0.4 0.6 0.8 1.0
0.01 0.10 1.00 10.00 100.00 1000.00
Time (min)

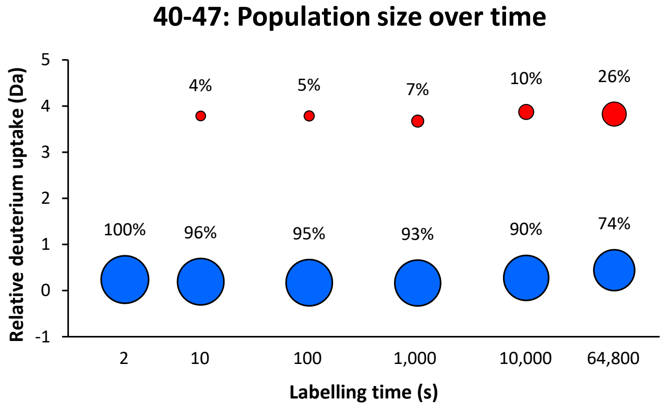
40-47: Population size over time
Relative deuterium uptake (Da)
-1 0 1 2 3 4 5
4% 5% 7% 10% 26%
100% 96% 95% 93% 90% 74%
2 10 100 1,000 10,000 64,800
Labelling time (s)

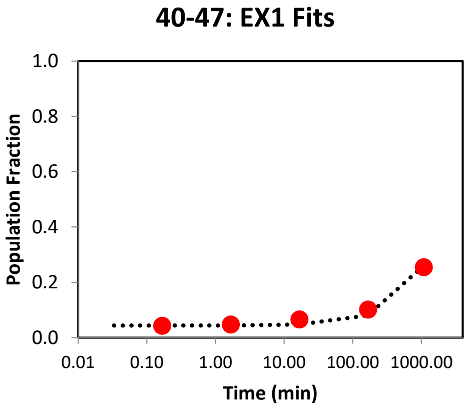
40-47: EX1 Fits
Population Fraction
0.0 0.2 0.4 0.6 0.8 1.0
0.01 0.10 1.00 10.00 100.00 1000.00
Time (min)

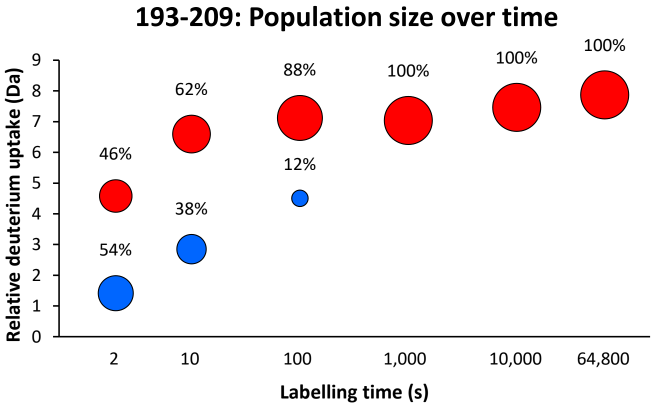
193-209: Population size over time
Relative deuterium uptake (Da)
0 1 2 3 4 5 6 7 8 9
46% 62% 88% 100% 100% 100%
54% 38% 12%
2 10 100 1,000 10,000 64,800
Labelling time (s)

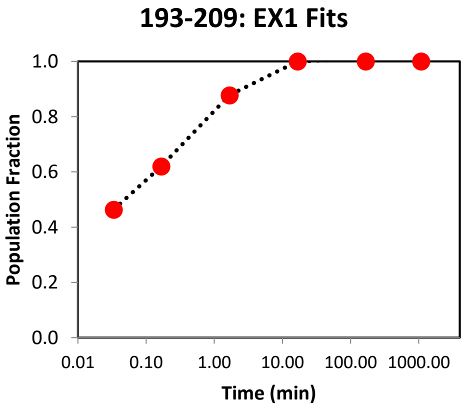
193-209: EX1 Fits
Population Fraction
0.0 0.2 0.4 0.6 0.8 1.0
0.01 0.10 1.00 10.00 100.00 1000.00
Time (min)

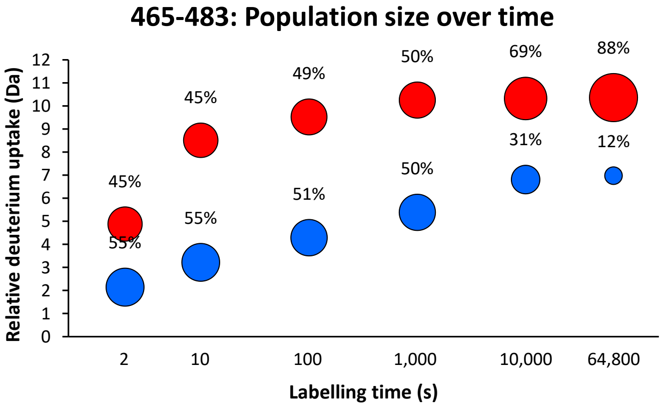
465-483: Population size over time
Relative deuterium uptake (Da)
0 1 2 3 4 5 6 7 8 9 10 11 12
45% 45% 49% 50% 69% 88%
55% 55% 51% 50% 31% 12%
2 10 100 1,000 10,000 64,800
Labelling time (s)

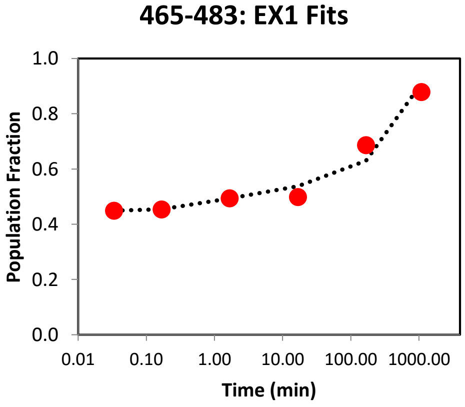
465-483: EX1 Fits
Population Fraction
0.0 0.2 0.4 0.6 0.8 1.0
0.01 0.10 1.00 10.00 100.00 1000.00
Time (min)

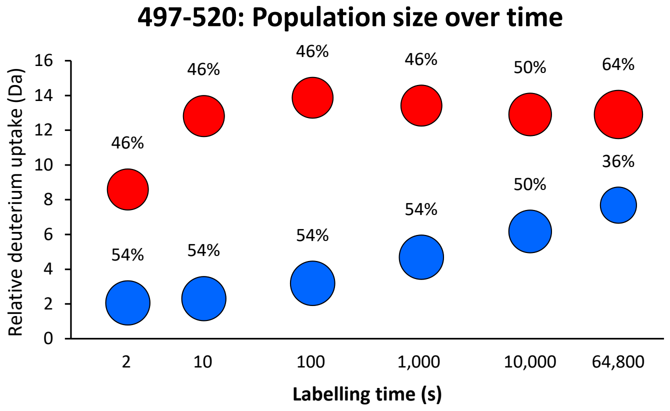
497-520: Population size over time
Relative deuterium uptake (Da)
0 2 4 6 8 10 12 14 16
46% 46% 46% 46% 50% 64%
54% 54% 54% 54% 50% 36%
2 10 100 1,000 10,000 64,800
Labelling time (s)

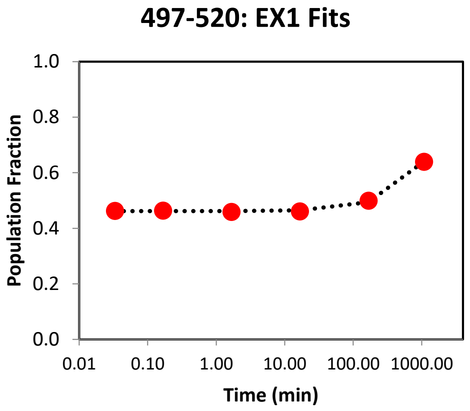
497-520: EX1 Fits
Population Fraction
0.0 0.2 0.4 0.6 0.8 1.0
0.01 0.10 1.00 10.00 100.00 1000.00
Time (min)

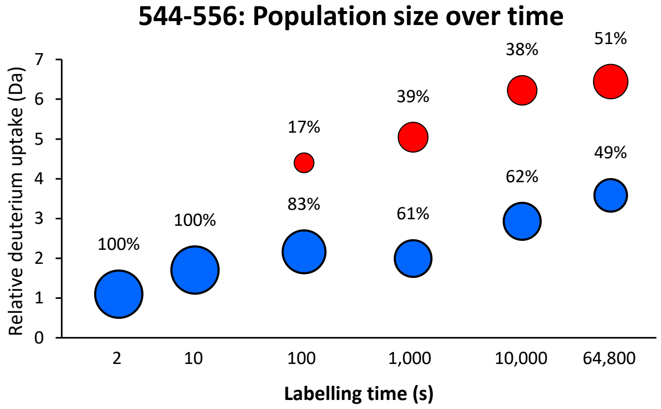
544-556: Population size over time
Relative deuterium uptake (Da)
0 1 2 3 4 5 6 7
17% 39% 38% 51%
100% 100% 83% 61% 62% 49%
2 10 100 1,000 10,000 64,800
Labelling time (s)

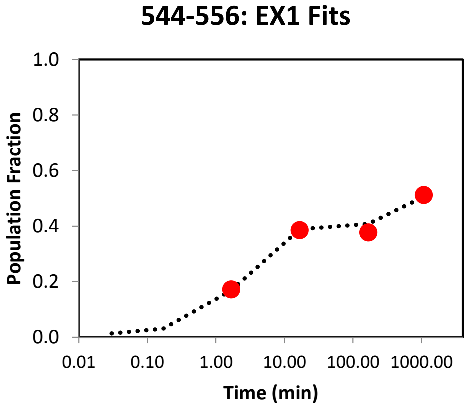
544-556: EX1 Fits
Population Fraction
0.0 0.2 0.4 0.6 0.8 1.0
0.01 0.10 1.00 10.00 100.00 1000.00
Time (min)

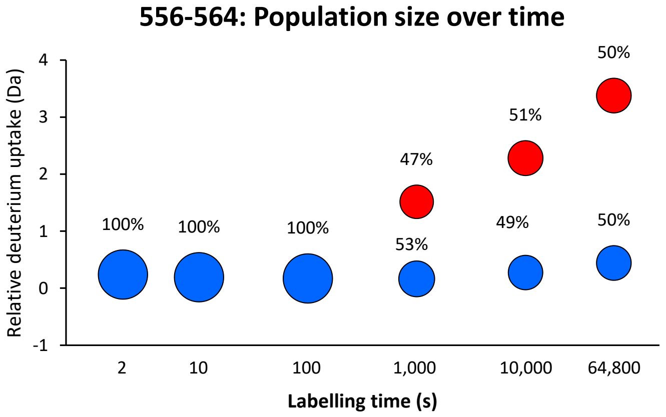
556-564: Population size over time
Relative deuterium uptake (Da)
-1 0 1 2 3 4
47% 51% 50%
100% 100% 100% 53% 49% 50%
2 10 100 1,000 10,000 64,800
Labelling time (s)

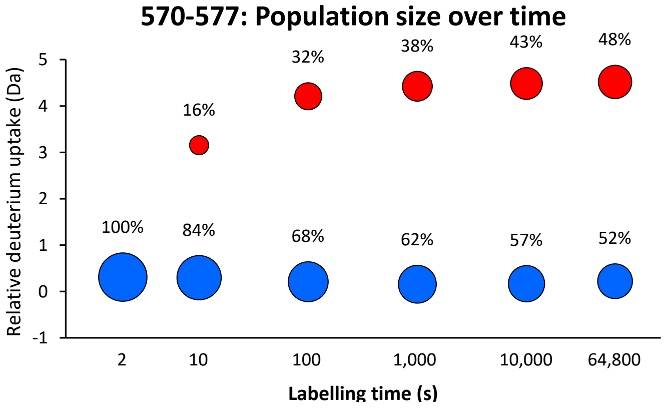
570-577: Population size over time
Relative deuterium uptake (Da)
-1 0 1 2 3 4 5
16% 32% 38% 43% 48%
100% 84% 68% 62% 57% 52%
2 10 100 1,000 10,000 64,800
Labelling time (s)

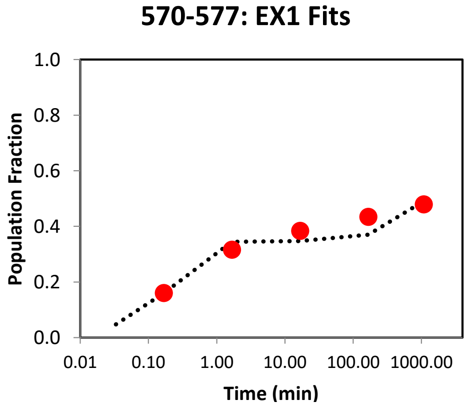
570-577: EX1 Fits
Population Fraction
0.0 0.2 0.4 0.6 0.8 1.0
0.01 0.10 1.00 10.00 100.00 1000.00
Time (min)

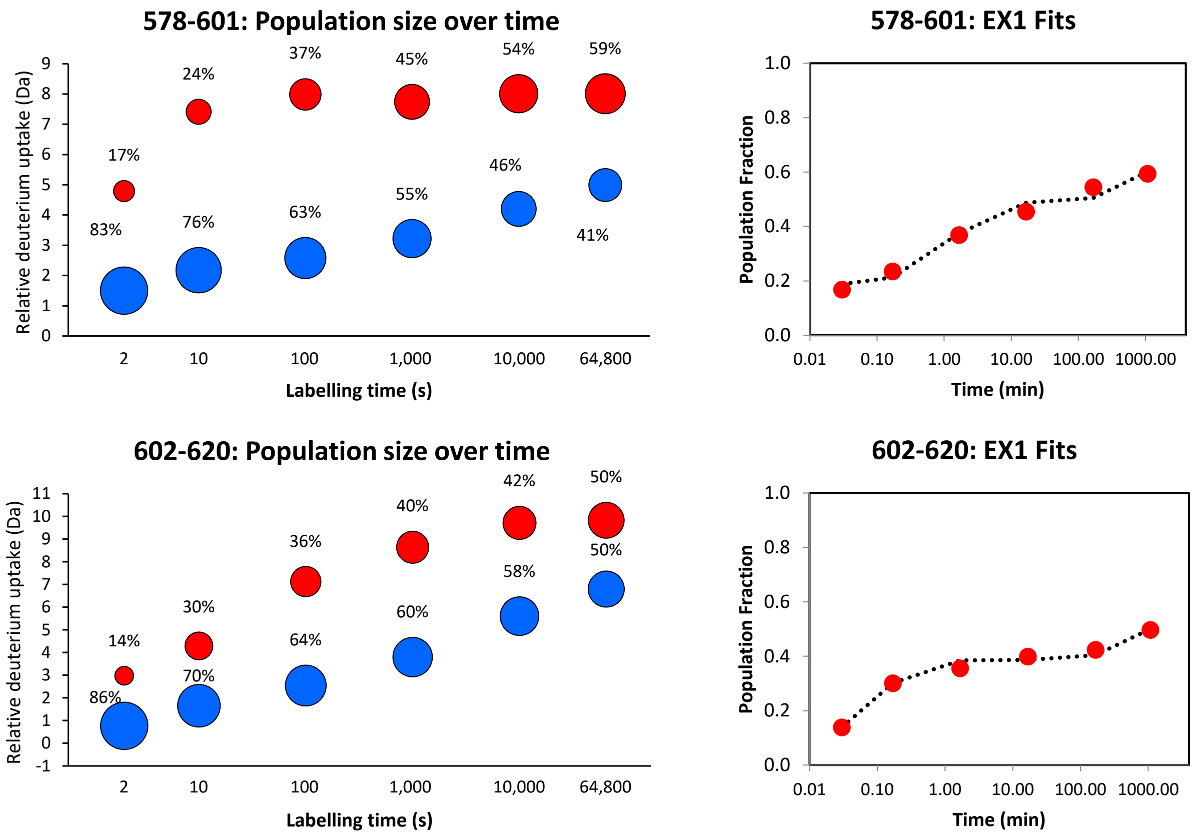

Supplementary Fig. S23 | Evolution of the low- and high-mass isotopic envelopes over the time points studied (left graphs) and EX1 fittings (right graphs) of the peptides fitted with EX-Express. Left graphs: blue bubbles: low-mass population; red bubbles: high-mass population; x-axis: time points studies; y-axis: deuterium uptake of the low- and high-mass populations; bubble size: relative fraction of the two populations. Right graphs: population fraction (y-axis) of the high-mass population over time (x-axis). Dashed line: EX1 fitting to an exponential decay function (see method session).

peptide	Max ΔHDX (Da)	MaxD (Da)	N	# NHs	start 1	final 1	kop 1 (min-1)	half-life 1 (min)	start 2	final 2	kop 2 (min-1)	half-life 2 (min)	Sum Chi^2	average half-life (min)
15-39	7.81	12.15	20	**12.16**	0.0303	0.2992	**0.0341**	20.339	0.01	0.6704	**0.0001**	5574.8	0.0007	**2797.6**
40-47	3.60	4.10	7	**5.81**	0.0439	0.9561	**0.0002**	2920.2					0.0009	**2920.2**
193-209	3.75	7.87	16	**7.21**	0.3998	0.4249	**3.9576**	0.1751	0.01	0.1753	**0.1718**	4.0340	0.0002	**2.1046**
465-483	5.29	10.77	16	**7.43**	0.4379	0.0782	**0.5021**	1.3804	0.01	0.4839	**0.0014**	479.13	0.0057	**240.26**
497-520	10.68	12.91	23	**17.99**	0.4519	0.0000	**2.0328**	0.3410	0.01	0.5481	**0.0004**	1896.1	0.0001	**948.24**
544-556	3.29	6.99	12	**5.34**	0.0000	0.3783	**0.3225**	2.1493	0.01	0.6217	**0.0002**	3570.4	0.0019	**1786.3**
556-564	2.94	4.70	7	**4.14**	0.4665									
570-577	4.30	4.54	7	**6.27**	0.0004	0.3344	**3.5260**	0.1966	0.01	0.6652	**0.0002**	3025.0	0.0064	**1512.6**
578-601	5.41	8.01	22	**14.05**	0.1728	0.3022	**0.6003**	1.1547	0.01	0.5250	**0.0002**	2984.1	0.0036	**1492.6**
602-620	4.84	10.09	17	**7.71**	0.0647	0.3099	**7.7176**	0.0898	0.01	0.6254	**0.0002**	3655.8	0.0014	**1827.9**

Supplementary Table S1 | Kinetic parameters for the correlated exchange calculated from bimodal envelope fitting shown in Fig. S15.

Data Set	Continuous labelling	Pulse labelling
HDX reaction details	120 mM HEPES, 150 mM NaCl, pH_{read} = 7.0, room temperature, final D_2O fraction 94.57%	
HDX time course	2 s, 10 s, 100 s, 1,000 s, 10,000 s and 18 h	10 s-pulse right after thawing and 10,000 s
HDX control samples	Maximally-labelled control	
Back-exchange (mean / IQR)	42.88% / 13.25%	
# of peptides followed	197	
Effective sequence coverage	98.9%	
Average peptide length / Redundancy	13.35 / 3.82	
Replicates (biological or technical)	Singlets	Duplicates (technical)
Repeatability	Not calculated (N/A)	

Supplementary Table S2 | Summary of the technical details of the HDX-MS data sets.
