# Supplementary material for: Cryo-EM led analysis of open and closed conformations of Chagas vaccine candidate TcPOP
Source: Nat Commun. 2025 Aug 5;16:7164. doi: 10.1038/s41467-025-62068-3 (PMC12325989; doi:10.1038/s41467-025-62068-3)
Supplement: Supplementary file 3 — Description of Additional Supplementary Files [file 41467_2025_62068_MOESM3_ESM.pdf]

Description of Additional Supplementary Files

File Name: Supplementary Data 1
Description: Cryo-EM data collection, refinement and validation statistics.

File Name: Supplementary Movie 1
Description: AlphaFold3 movie of poses of collagen bound to TcPOP.

File Name: Supplementary Movie 2
Description: 3D projection of immune serum from mouse 1 (IM1S) binding to amastigotes and trypomastigotes.

File Name: Supplementary Movie 3
Description: 3D projection of monoclonal antibody 1 (IM1-mAb1) binding to amastigotes and trypomastigotes.
