# Supplementary material for: Cryo-EM led analysis of open and closed conformations of Chagas vaccine candidate TcPOP
Source: Nat Commun. 2025 Aug 5;16:7164. doi: 10.1038/s41467-025-62068-3 (PMC12325989; doi:10.1038/s41467-025-62068-3)
Supplement: Supplementary file 4 — Supplementary Data 1 [file 41467_2025_62068_MOESM4_ESM.pdf]

Supplementary Data Table 1 Cryo-EM data collection, refinement and validation statistics

	#1 Closed (EMDB-52215) (PDB 9HJI)	#2 Open (EMDB-52216) (PDB 9HJJ)
**Data collection and processing**		
Magnification	130,000	130,000
Voltage (kV)	300	300
Electron exposure (e–/Å^2)	76.43	76.43
Defocus range (μm)	-2.3 to -0.6	-2.3 to -0.6
Pixel size (Å)	0.656	0.656
Symmetry imposed	C1	C1
Initial particle images (no.)	45,400,101	45,400,101
Final particle images (no.)	847,556	518,841
Map resolution (Å)	3.57	3.82
FSC threshold	0.143	0.143
Map resolution range (Å)	2.8 to 6	2.9 to 6
**Refinement**		
Initial model used (PDB code)	*Abinitio*	*Abinitio*
Model resolution (Å)	3.57	3.82
FSC threshold	0.143	0.143
Model resolution range (Å)	3.5 to 6	3.8 to 4
Map sharpening *B* factor (Å^2)	-85.1887	-91.3478
Model composition		
Non-hydrogen atoms	5532	5532
Protein residues	697	697
Ligands	0	0
B factors (Å^2)		
Protein	66	126
Ligand	0	0
R.m.s. deviations		
Bond lengths (Å)	0.27	0.28
Bond angles (°)	0.56	0.57
Validation		
MolProbity score	2.42	2.54
Clashscore	13.95	14.79
Poor rotamers (%)	3.51	2.84
Ramachandran plot		
Favored (%)	94.39	90.79
Allowed (%)	100	100
Disallowed (%)	0	0
