# Supplementary material for: Cryo-EM led analysis of open and closed conformations of Chagas vaccine candidate TcPOP
Source: Nat Commun. 2025 Aug 5;16:7164. doi: 10.1038/s41467-025-62068-3 (PMC12325989; doi:10.1038/s41467-025-62068-3)
Supplement: Supplementary file 8 — Reporting Summary [file 41467_2025_62068_MOESM8_ESM.pdf]

nature portfolio

Corresponding author(s): Dr Ivan Campeotto

Last updated by author(s): Dec 18, 2024

Reporting Summary

Nature Portfolio wishes to improve the reproducibility of the work that we publish. This form provides structure for consistency and transparency in reporting. For further information on Nature Portfolio policies, see our Editorial Policies and the Editorial Policy Checklist.

Statistics

For all statistical analyses, confirm that the following items are present in the figure legend, table legend, main text, or Methods section.

n/a	Confirmed	
☐	☒	The exact sample size (*n*) for each experimental group/condition, given as a discrete number and unit of measurement
☐	☒	A statement on whether measurements were taken from distinct samples or whether the same sample was measured repeatedly
☐	☒	The statistical test(s) used AND whether they are one- or two-sided<br>*Only common tests should be described solely by name; describe more complex techniques in the Methods section.*
☒	☐	A description of all covariates tested
☒	☐	A description of any assumptions or corrections, such as tests of normality and adjustment for multiple comparisons
☐	☒	A full description of the statistical parameters including central tendency (e.g. means) or other basic estimates (e.g. regression coefficient) AND variation (e.g. standard deviation) or associated estimates of uncertainty (e.g. confidence intervals)
☐	☒	For null hypothesis testing, the test statistic (e.g. *F*, *t*, *r*) with confidence intervals, effect sizes, degrees of freedom and *P* value noted<br>*Give P values as exact values whenever suitable.*
☒	☐	For Bayesian analysis, information on the choice of priors and Markov chain Monte Carlo settings
☒	☐	For hierarchical and complex designs, identification of the appropriate level for tests and full reporting of outcomes
☒	☐	Estimates of effect sizes (e.g. Cohen's *d*, Pearson's *r*), indicating how they were calculated

Our web collection on statistics for biologists contains articles on many of the points above.

Software and code

Policy information about availability of computer code

Data collection	Data collection and processing software used are described in the methods section and are commercially available or openly accessible. Software used for CryoEM data collection using EPU version 3.4.0.5704REL and 3.6 (ThermoFisher). HDX-MS data were collected via MassLynX (Waters). NIS-Elements software was used for microscopy images acquisition. Attune Cytometric Software was used for flow cytometry data collection.
Data analysis	Data analysis was performed as described in the methods section using openly accessible software. Software used for data processing and structure determination is the standard in the field and is all freely available to academic users. RELION v5.0 beta was used for CryoEM data processing and analysis, followed by COOT v0.9.8.91 and PHENIX-1.21-5207 for model refinement. ASTRA6 was used to collect and analyse the SEC-MALS data, Octet BLI Discovery 12.2.2.20 was used for BLI data processing and analysis. GROMACS 2021.2-fosscuda-2020b was used to perform molecular dynamics simulation and ATSAS 3.2.1 software packages were used for SEC-SAXS data analysis. GraphPad Prism version 9 was used to generate graphs, PyMOL version 2.5 for structure visualization. HDX-MS data analysis was performed with Protein Lynx Global Server (PLGS) 3.0 (Waters), DynamX 3.0 (Waters) and open access HX-Express 3.0 (https://www.hxms.com/HXExpress/). NIS-Elements for microscopy images and videos processing. FlowJo 10.10.O for data analysis in Flow cytometry assays.

For manuscripts utilizing custom algorithms or software that are central to the research but not yet described in published literature, software must be made available to editors and reviewers. We strongly encourage code deposition in a community repository (e.g. GitHub). See the Nature Portfolio guidelines for submitting code & software for further information.

April 2023

Data

Policy information about availability of data

All manuscripts must include a data availability statement. This statement should provide the following information, where applicable:

- Accession codes, unique identifiers, or web links for publicly available datasets
- A description of any restrictions on data availability
- For clinical datasets or third party data, please ensure that the statement adheres to our policy

Coordinates and structure factors derived in this study have been deposited in the Protein Data Bank under accession codes 9HJI and 9HJJ

Research involving human participants, their data, or biological material

Policy information about studies with human participants or human data. See also policy information about sex, gender (identity/presentation), and sexual orientation and race, ethnicity and racism.

Reporting on sex and gender	N/A
Reporting on race, ethnicity, or other socially relevant groupings	N/A
Population characteristics	N/A
Recruitment	N/A
Ethics oversight	N/A

Note that full information on the approval of the study protocol must also be provided in the manuscript.

Field-specific reporting

Please select the one below that is the best fit for your research. If you are not sure, read the appropriate sections before making your selection.

- [x] Life sciences
- [ ] Behavioural & social sciences
- [ ] Ecological, evolutionary & environmental sciences

For a reference copy of the document with all sections, see nature.com/documents/nr-reporting-summary-flat.pdf

Life sciences study design

All studies must disclose on these points even when the disclosure is negative.

Sample size	For immunisation studies, no sample size calculation was performed and group sizes were chosen as to minimise animal use whilst still provide sufficient sample size to see significant differences based on previous experiments.
Data exclusions	As a standard in cryoEM workflow, particles not contributing to the appropriate class were excluded from the final reconstruction.
Replication	The number of repeats for each relevant experiment were mentioned in the legends and method sections. Typically, all the experiments were conducted in triplicates (unless stated in the method section), however DSF measurements were performed in quadruplicates. All the attempts shows similar outcomes to the data shown.
Randomization	No randomisation was conducted as no decisions about inclusion or exclusion of data were taken and experiments were designed such that single parameters were varied during the experiment. All data was included in analysis.
Blinding	No blinding was conducted as no subjective decisions about data inclusion were involved in data collection.

Reporting for specific materials, systems and methods

We require information from authors about some types of materials, experimental systems and methods used in many studies. Here, indicate whether each material, system or method listed is relevant to your study. If you are not sure if a list item applies to your research, read the appropriate section before selecting a response.

Materials & experimental systems

n/a	Involved in the study
☐	☒ Antibodies
☐	☒ Eukaryotic cell lines
☒	☐ Palaeontology and archaeology
☐	☒ Animals and other organisms
☒	☐ Clinical data
☒	☐ Dual use research of concern
☒	☐ Plants

Methods

n/a	Involved in the study
☒	☐ ChIP-seq
☐	☒ Flow cytometry
☒	☐ MRI-based neuroimaging

Antibodies

Antibodies used	Monoclonal antibodies mAb1, mAb2, mAb3 are not commercially available and were produced and validated as described in the current publication that were used at concentrates as described in the text. Moreover, Hexa-His HRP antibody was used for detection of the protein samples/lysates using western blot, followed by rabbit anti-mouse IgG during the ELISA experiments. Goat anti-mouse AF488 used for microscopy assays.
Validation	All antibodies have been validated through Western blotting, ELISA and BLI in this publication.

Eukaryotic cell lines

Policy information about cell lines and Sex and Gender in Research

Cell line source(s)	- Hybridomas were only used to produce protein.<br>- Trypanosoma cruzi CLBr (DTU VI) genetically modified with pTREX2-LucRe9h-mScarlet (GenBank accession #PP333636)<br>- COLO-N680 cell line (RRID:CVCL_1131) from Squamous cell carcinoma of the esophagus in Homo sapiens (female).
Authentication	- For Hybridomas no authentication was conducted as cells were only used to produce protein for analysis and the protein was validated instead of the cells.<br>- Cells and protozoa are clearly labelled since they were acquired in the collection bank of the lab and they do correspond with the identification provided.
Mycoplasma contamination	Tested
Commonly misidentified lines (See ICLAC register)	None were used

Animals and other research organisms

Policy information about studies involving animals; ARRIVE guidelines recommended for reporting animal research, and Sex and Gender in Research

Laboratory animals	Six weeks old female BALB/c ByJR mice were used for antibody production.
Wild animals	None
Reporting on sex	The mice used were female
Field-collected samples	None
Ethics oversight	The conducted animal research strictly conformed to the standards delineated by the Federation of European Laboratory Animal Science Associations (FELASA). Ethical clearance for the experimental methodologies was granted by the Danish Animal Experiment Inspectorate, as indicated by their approval number 2018-15-0201-01541.

Note that full information on the approval of the study protocol must also be provided in the manuscript.

Plants

Seed stocks	N/A
Novel plant genotypes	N/A
Authentication	N/A

Flow Cytometry

Plots

Confirm that:

- [x] The axis labels state the marker and fluorochrome used (e.g. CD4-FITC).
- [x] The axis scales are clearly visible. Include numbers along axes only for bottom left plot of group (a 'group' is an analysis of identical markers).
- [x] All plots are contour plots with outliers or pseudocolor plots.
- [x] A numerical value for number of cells or percentage (with statistics) is provided.

Methodology

Sample preparation	After trypsinization cells were fixed with PFA, washed with PBS and resuspended in Flow cytometry staining buffer as described in the methods section.
Instrument	Thermo Fisher Scientific Attune NxT
Software	For data collection Attune Cytometric Software was used and data analysis has been performed with FlowJo 10.10.0
Cell population abundance	> 35,000 events (events) as an average were counted per sample.
Gating strategy	1. FSC/SSC was used to identify the cells from the debris or extracellular parasites.<br>2. FSC-A/FSC-H was used to identify single cells.<br>3. Violet laser 1-A/FSC-A was used to confirm the Hoechst stained cells.<br>4. Yellow laser 1-A/FSC-A was used to distinguish between non infected and infected cells with the parasites expressing mScarlet fluorescent protein. (A non infected culture was used as control to adjust the non infected gate).

- [x] Tick this box to confirm that a figure exemplifying the gating strategy is provided in the Supplementary Information.
